# From Farm to Flavor: Carbon and Biodiversity Footprint of the Global Spice Market

**DOI:** 10.1021/acs.est.5c04846

**Published:** 2026-04-07

**Authors:** Corinna Bolliger, René Itten, Matthias Stucki

**Affiliations:** Institute of Natural Resource Sciences, Life Cycle Assessment Research Group, Zurich University of Applied Sciences (ZHAW), Grüentalstrasse 14, 8820 Wädenswil, Switzerland

**Keywords:** life cycle assessment, biodiversity, spices, climate impact, land use change, LCA, food systems, agriculture

## Abstract

Agriculture generates roughly one-third of global greenhouse gas (GHG) emissions and is the primary driver of biodiversity loss, underscoring the need to assess the environmental impact of all crops. However, the spice sector has received little attention, with only a few life cycle assessment (LCA) studies available. This study evaluates the carbon and biodiversity footprint of the global spice sector and situates the sector within the broader agricultural system. Climate impacts were quantified using the IPCC GWP100a method, while biodiversity impacts were assessed using LC-IMPACT and a land-use-induced biodiversity assessment method (the high-resolution land use intensities and fragmentation method, LUIF). Indirect drivers of species loss were found to be significant, highlighting the importance of biodiversity assessment beyond only land-use-induced impacts. To capture these effects more comprehensively, the LUIF method was integrated into the LC-IMPACT framework. Results indicate that the global spice sector emits between 41.4 and 64.8 Mt CO_2_-eq annually and causes approximately 7.2 × 10^–4^ PDF·yr (potentially disappeared fraction of species), including land plants and vertebrates. Although spices account for only 0.18% of agri-food sector turnover and 0.06% of global crop production volume, they contribute 0.33% of agricultural GHG emissions.

## Graphical abstract

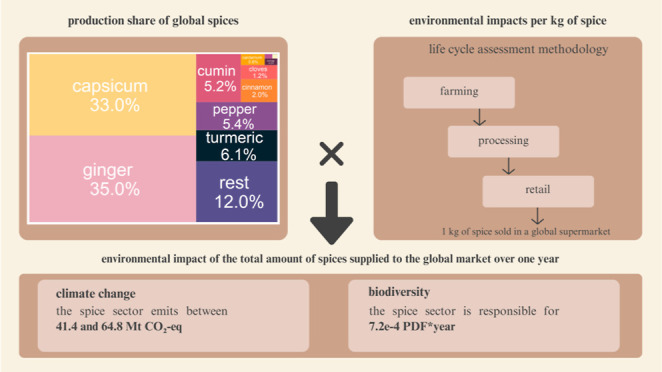

## 1 Introduction

The agricultural sector is the leading driver of global biodiversity loss^1^ and is responsible for approximately one-third of the global greenhouse gas (GHG) emissions.^2^ Spatially explicit MRIO modeling demonstrates that agri-food imports are key drivers of biodiversity loss linked to recent land-use change,^3–6^ and that impacts are strongly associated with tropical export crops.^3^ In an increasingly globalized world, environmental impacts often occur in one region while consumption takes place elsewhere.^7,8^ Spice cultivation often takes place in these tropical high-biodiversity regions, where large tracts of land are dedicated to export-oriented crop production.^7,9^

Although spices account for only 0.06% of global food crop production,^10^ they hold major cultural and culinary importance worldwide,^11^ with demand steadily rising due to globalization and increasingly diverse food preferences.^12^ The global spice market, valued at $15.5 billion in 2022, is projected to reach $35.42 billion by 2029.^12^ Globally, spice consumption in 2019 reached 1.58 kg per person annually.^13^ The rapid growth of the spice sector also presents significant environmental challenges.^14^ Increasing global and domestic demand has intensified spice cultivation, thereby amplifying pressure on ecosystems.^15^ Spice cultivation, especially conventional methods, contributes to biodiversity loss through land use change (LUC), reduced crop genetic diversity, heavy reliance on pesticides and synthetic fertilizers, unregulated water extraction, and insufficient management of invasive species.^15^ Despite land-use change being a major driver of biodiversity loss, the EU’s deforestation regulation (EUDR), which restricts imports of goods linked to post-2020 deforestation, currently excludes the spice sector. This exemption allows unsustainable practices to persist, which further accelerates biodiversity loss.^16^

Existing LCAs are typically limited to single-spice case studies, geographically restricted, and primarily focused on climate change, without providing a comprehensive sector-level assessment. Across some available studies on saffron,^17,18^ pepper,^19^ and vanilla,^20^ the cultivation phase consistently emerges as the hotspot, primarily driven by fertilizer production and associated emissions. In contrast, other globally traded spices are scarcely represented in peer-reviewed LCA literature.

Quantified biodiversity impacts are increasingly required to support the implementation of the global biodiversity framework where biodiversity is valued, conserved, and restored,^21^ and for achieving UN sustainable development goal 12 on responsible consumption and production.^22^ Such assessment is also central to the Farm to Fork strategy under the European Green Deal which aims to sustainably transition food systems toward neutral or positive environmental impacts, halt biodiversity loss, and mitigated climate change.^23^

Despite the policy priorities, the spice sector remains under-researched, with only limited life cycle assessment (LCA) research and no comprehensive estimates of its environmental impact. As highlighted by Song et al.,^24^ expanding biodiversity assessment coverage to specialty crops is necessary. To address this gap, the present study provides a global, comprehensive environmental assessment of the spice sector, focusing on climate and biodiversity impacts. Initially, all spices are analyzed on a per-kilogram basis to identify environmental hotspots and to compare organic and conventional production systems. These results are then scaled up using global production volumes to estimate sector-wide impacts. The results therefore offer new insights into the sector’s global contribution to climate change and biodiversity loss.

## 2 Methods

In this study, an LCA was employed to evaluate the environmental impacts of the global spice sector from raw material extraction to disposal (i.e., cradle to grave) in accordance with ISO 14040 and 14,044 standards.^25,26^ The analysis was performed using SimaPro,^27^ a professional LCA software designed to model and quantify environmental impacts across a product’s life cycle. SimaPro structures inventory data into interconnected product systems, linking foreground data on inputs (materials, energy) and outputs (emissions, waste) with background data from databases such as ecoinvent. It also automates impact calculations and supports Monte Carlo simulations to address uncertainties.^27^ SimaPro was used to compile and organize the input data, connect foreground processes to background data from the ecoinvent database, and perform the impact assessment calculations for both the LC-IMPACT_original_ and EF 3.1 methods.

### 2.1 Goal and Scope

This study aims to assess the environmental impacts of the global spice sector, with a particular focus on biodiversity impacts and climate change. The central objective is to conduct a sector-wide LCA of the most relevant spices and scale the results using global production volumes. Accordingly, the functional unit of this study is defined as the total amount of spices supplied to the global market over one year, encompassing spices used in both food and nonfood applications.

A case-specific modeling approach was applied to 12 spices: capsicum, cardamom, cinnamon, cloves, cumin, ginger, nutmeg, mace, pepper, saffron, turmeric, and vanilla (SI1 Figure 17). This selection was based on (i) each spice’s share of the fresh production mass, (ii) economic value contribution,^10^ (iii) classification by the harmonized system convention,^28^ and (iv) the spice categories defined by the European Spice Association.^29^ All 12 spices were modeled under conventional cultivation systems, and five spices (cinnamon, cloves, ginger, turmeric, and vanilla) were additionally modeled under an organic cultivation system. For individual case studies, a reporting unit of 1 kg of spice sold in global supermarkets in 2024 was used. The results per kilogram were subsequently upscaled by the global production volume of each spice to estimate the total environmental impact of the global spice market. The overarching goal is therefore to quantify both the carbon and biodiversity footprint of the global spice sector.

Where primary data was not available, production was modeled using literature-based data from major producing countries. All assessments include the stages of cultivation, processing, and retail, following the general production system for the 1 kg reporting unit of spices (Figure 1). Detailed production systems are provided in the Supporting Information (SI1 Figures 1–11).

**Figure 1:**
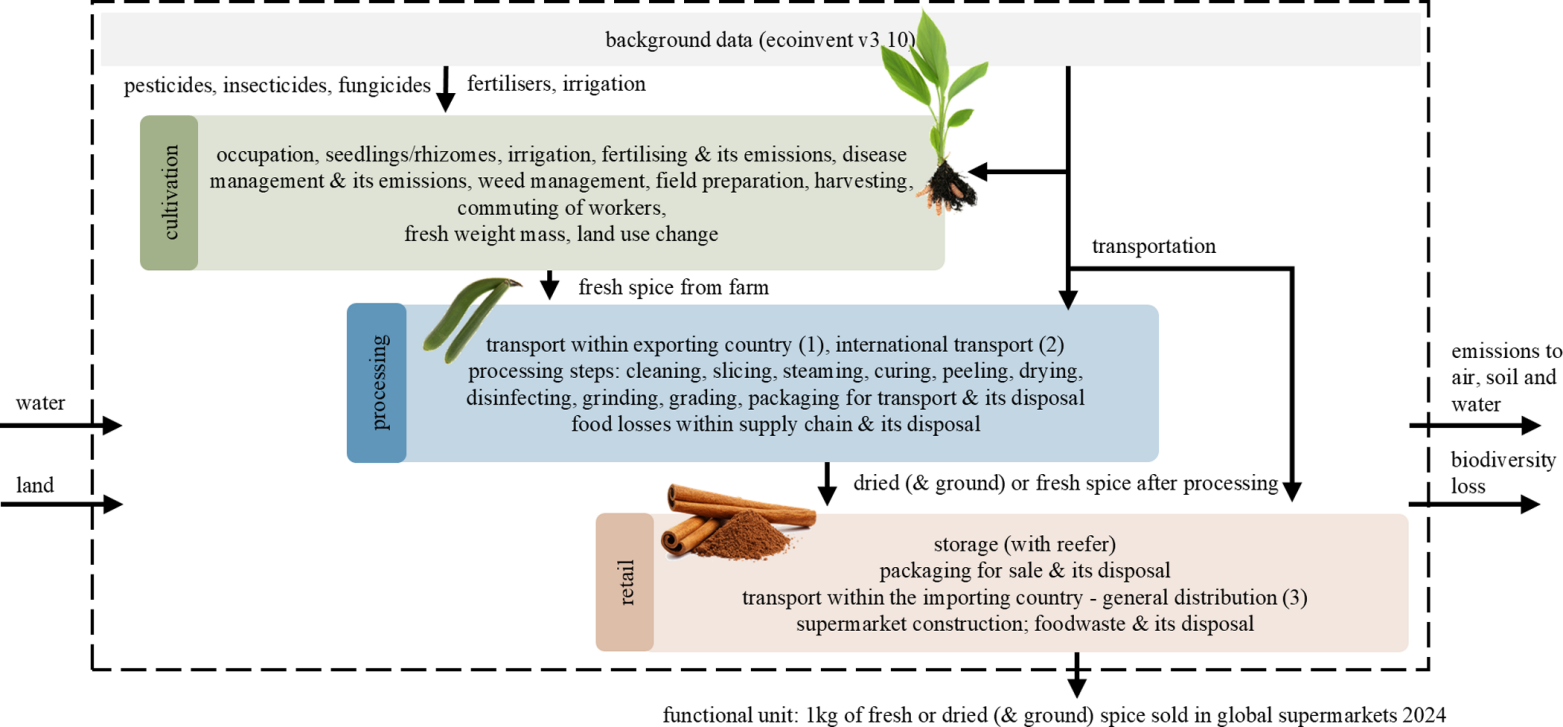
General production system of spices consists of three stages: cultivation, processing, and retail. The reporting unit is 1 kg of fresh or dried (& ground) spice, sold in global supermarkets in 2024.

### 2.2 Inventory Analysis and Uncertainty Assessment

To develop the life cycle inventory (LCI), detailed production data for each assessed spice was required. Cultivation processes were modeled primarily using data reported in the scientific literature,^30–39^ and were complemented with newly acquired primary data from a Swiss-based spice trader supplying organically produced vanilla, cinnamon, turmeric, cloves, and ginger from Madagascar. Additionally, background data from the ecoinvent v3.10 database, based on the “allocation, cut-off” system model, was incorporated.^20^

The inventory analysis followed a structured approach encompassing three main lifecycle stages: cultivation (fresh spice from the farm), processing (processed, often dried spice), and the retail stage (packaged spice sold to consumers). Although each spice was modeled using case-specific data, a set of general assumptions and consistent methodological rules was applied across all spice systems:

For the cultivation stage, all emissions relating to fertilizers, pesticides, and irrigation were calculated according to the World Food LCA Database (WFLDB) methodological guidelines for the life cycle inventory.^40^ It was assumed that 100% of the heavy metals enter the soil, as these metals only become mobile under high pH conditions. It is explicitly stated if high pH conditions are present in any individual case study. Modeling pesticide impacts requires data on both active ingredients and total application quantities of herbicides, fungicides, and insecticides. It was assumed that 100% of the pesticides remain in the soil (SI3). Direct LUC emissions were quantified using the 2021 Direct Land Use Change Assessment Tool developed by Blonk Consultants.^41^ This method accounts for carbon stocks in soil, vegetation, and dead organic matter, comparing the carbon stored prior to land conversion (reference situation) with that stored in existing plantations. Emissions are distributed evenly over a 20 year period following land transformation.^42^ To assess the influence of time span variability, an additional analysis over a 50 year period was conducted (SI3).

Transport was modeled using three stages: (1) transportation within the production countries, (2) international transport from the exporting to the importing country, and (3) transportation within the importing region. Transport types 1 and 2 are included in the processing stage, while transport type 3 is part of the retail stage. Trade flows were identified using the International Trade Center^43^ and FAOSTAT data,^10^ and transport distances were calculated using the SeaRates Cargo Calculator.^44^ Detailed assumptions are provided in SI1 Chapter 13.

Generation of food waste was included in all models based on the UN Food Waste Index Report 2024.^45^ Globally, 19% of food waste occurs in retail, food service, and households (allocated to the retail stage), and 13% arises along the supply chain (allocated to the processing stage) from postharvest to the point of sale.^45^ Disposal of this waste was modeled within the same production stage in which it was generated.

A distinction was made between two types of packaging (SI1, Chapter 12): transport packaging, which is integrated into the processing stage, and retail packaging, which is accounted for in the retail stage. Transportation packaging materials were determined based on a case-specific basis, while retail packaging was consistently modeled as glass jars, except for saffron and cinnamon. Case-specific data from the literature were used for modeling fresh turmeric and ginger, which are sold in thin plastic packaging. For modeling the glass packaging, primary data were obtained through a supermarket analysis in which spices in glass jars were weighed. These measured quantities, varying by spice type and form (whole or ground), were then integrated into the LCA modeling.

An economic allocation was applied to organic cinnamon, organic turmeric, and organic ginger intercropped with cassava, banana, and pineapple, respectively. An economic allocation was also performed for nutmeg and mace, which originate from the same tree. Where prewashing occurred for any spices, water usage was estimated at 2 L/kg based on a comparative study on vegetable production.^46^ For additional boiling, an extra 0.5 L/kg was assumed.

The following assumptions were made for organic models based on primary data. Some organic spices undergo pressure disinfection, steam sterilization, and grinding. For modeling the steam sterilization process, specific energy consumption was considered. A study by Mohd Nadzim et al.^47^ simulated energy use during sterilization, reporting 5259 MJ/h inputs for 14,580 kg, equating to 0.36 MJ/kg of product. This aligns with the findings of Ladha-Sabur et al.,^48^ who reported an average electricity consumption of 1.12 MJ/kg for processed fruits and vegetables, including steps like sterilization and storage, and reflecting higher energy requirements. Since the load percentage strongly influences energy demand in sterilization processes,^49^ a conservative value of 1.12 MJ/kg of spice was assumed for the entire processing stage of the organic spices.

The Supporting Information (SI1–SI3) provides data sets, calculations, assumptions, and estimations used in the LCI. Where process or material data were unavailable, assumptions and estimates were applied.

To quantify uncertainty in the life cycle inventory and model structure, a Monte Carlo simulation was conducted. Monte Carlo analysis repeatedly samples from the probability distributions assigned to each input parameter and recalculates the model outputs, producing a distribution of possible results. In this study, 10,000 iterations were performed to ensure the stability of the results. Uncertainties appearing from the characterization factors (CFs) were not included.

All input data were assigned log–normal uncertainty distributions, consistent with the uncertainty representation in the ecoinvent database, where exchange values are modeled as log–normal due to their strictly positive nature and the multiplicative structure of pedigree-based uncertainty factors.^50–53^ Lognormal distribution is characterized by a standard deviation which was estimated using the pedigree matrix approach originally developed by Weidema & Wesnaes.^50^ This method combines (i) a basic uncertainty factor, determined by the type of data, with (ii) scores for six data-quality indicators: reliability, completeness, temporal correlation, geographical correlation, technological correlation, and sample size. The corresponding pedigree scores for background processes were taken directly from the ecoinvent database, where each data set includes a predefined sequence of six pedigree values and their associated uncertainty factors.^51,54^

Thus, for all background data, the existing ecoinvent uncertainty distributions were used, while foreground data were assigned uncertainty based on their evaluated pedigree scores. After defining the distribution parameters for all inventory inputs, the Monte Carlo simulation propagated these uncertainties through the model to quantify their influence on the final LCA results.

### 2.3 Impact Assessment

The impact assessment focuses on climate and biodiversity impacts. The environmental footprint method (EF 3.1), recommended by the European Commission,^55^ was used to assess the environmental impacts at the midpoint level and was applied using SimaPro. This method also includes the latest global warming potential (GWP) method from IPCC 2021.^56^ The analysis concentrated on GWP with a 100 year time horizon.^56^ Results for all other midpoint impact categories are provided in SI1 Chapter 16.

Biodiversity impacts were assessed at two levels. First, land-use-induced biodiversity loss is quantified using the newest Land Use Intensities and Fragmentation method of Scherer et al. (2023), referred to here as LUIF.^57^ Second, LC-IMPACT is used to estimate (total) biodiversity damage at the level of the area of protection ecosystem quality by aggregating characterization factors across several impact categories, including biodiversity loss induced by climate change and land use. Due to methodological uncertainties related to land transformation, only characterization factors for land occupation were applied.

LUIF (2023) is an updated method for assessing biodiversity impacts arising from land use intensities and fragmentation,^57^ building on earlier versions of the method.^42,58^ Its final unit, the potentially disappeared fraction of species (PDF), reflects impacts attributable solely to land use, excluding factors like climate change, acidification, eutrophication, or pollution. While the characterization factors (CFs) account for both local and global biodiversity losses, this study focused exclusively on global species loss, representing irreversible extinction, whereas regional losses may be reversible.^57^ Given the global scope and large-scale focus, average CFs were applied as recommended.^57^ The application was conducted using CFs specific for different ecoregions and taxonomic groups (SI5). For the calculation, animals (only terrestrial vertebrates) and plants were weighed equally, and within the animal kingdom, amphibians, birds, mammals, and reptiles were also assigned equal weight. Spices grown in agroforestry or intercropping systems were classified as cropland under minimal intensity. Monoculture spices with moderate fertilizer and pesticide inputs were assigned to light-intensity cropland. Tree-grown spices, such as cloves, were categorized as plantations with minimal or light-intensity management. Since the newest version of LUIF is not yet implemented in SimaPro, the regionalized assessment was carried out in Excel (SI5).

LC-IMPACT_original_ (2020) provides an LCA methodology for assessing four areas of protection, including ecosystem quality which covers terrestrial, aquatic, and marine environments and is expressed in PDF·yr.^59^ For this assessment, CFs with the average modeling choice, a 100 year time horizon, and all impacts (certain and uncertain impacts) were used to assess the species.

LC-IMPACT_updated:_ the LC-IMPACT_original_ method incorporates land-use characterization factors from Chaudhary et al. (2015), which are regionalized only at the country level in SimaPro. In this study, LC-IMPACT_original_ results were first calculated in SimaPro.^58^ The land-use contribution was then subtracted in Excel and replaced with the corresponding regionalized LUIF land use contribution, producing LC-IMPACT_updated_ results. These incorporate the most recent, ecoregion-specific land-use characterization factors from Scherer et al. 2023 (see Chapter [Sec sec4.3] for discussion).^57^

Both the LC-Impact and LUIF methods express biodiversity loss in PDF·yr, which quantify the relative fraction of global species committed to extinction due to anthropogenic pressures such as land use.^59^

## 3 Results and Interpretation

### 3.1 Carbon Footprint per kg of Spice

Saffron shows the highest climate intensity with 121 kg CO_2_-eq/kg, followed by vanilla (43.1 kg CO_2_-eq/kg), cardamom (26 kg CO_2_-eq/kg), pepper (23 kg CO_2_-eq/kg), and capsicum (16.8 kg CO_2_-eq/kg), assuming all are produced conventionally (Figure 2). Organic spices generally exhibit lower GHG emissions than their conventional counterparts (except for turmeric and ginger), with delta differences of 4.8 kg for cloves, 5.6 kg for cinnamon, and 26.3 kg for vanilla. For most conventional spices, cultivation is the primary source of GHG emissions, driven primarily by mineral fertilizer and pesticide application and by LUC emissions. LUC contributes significantly to the total GHG emissions of saffron (75.9%), cumin (47.6%), cinnamon (42.7%), ginger (17.2%), and cloves (13.9%). These results align with FAO and IPCC reports estimating LUC to account for approximately 20% of global GHG emissions^2^ and 13–21% of anthropogenic emissions between 2010 and 2019.^56^ Beyond emissions from LUC and fertilizer, fossil fuel consumption and energy–intensive processes are vital contributors to food product emissions.^60^ In organic spice production, lower impacts from the cultivation stage shift the dominant hotspot to the retail stage, particularly glass jar packaging, except for vanilla, where air transport is the largest contributor (Figure 2, bottom). LCA studies on vegetables support this pattern, with packaging contributing between 7% and 54% of total emissions.^61^ Spices sold unpackaged, such as fresh turmeric and ginger, or in minimal packaging like plastic bags (e.g., saffron, cinnamon quills), have negligible or no packaging-related impact. Overall, postharvest stages such as packaging and transport can offset or even outweigh some of the GHG emissions savings achieved through organic cultivation.^60^

**Figure 2:**
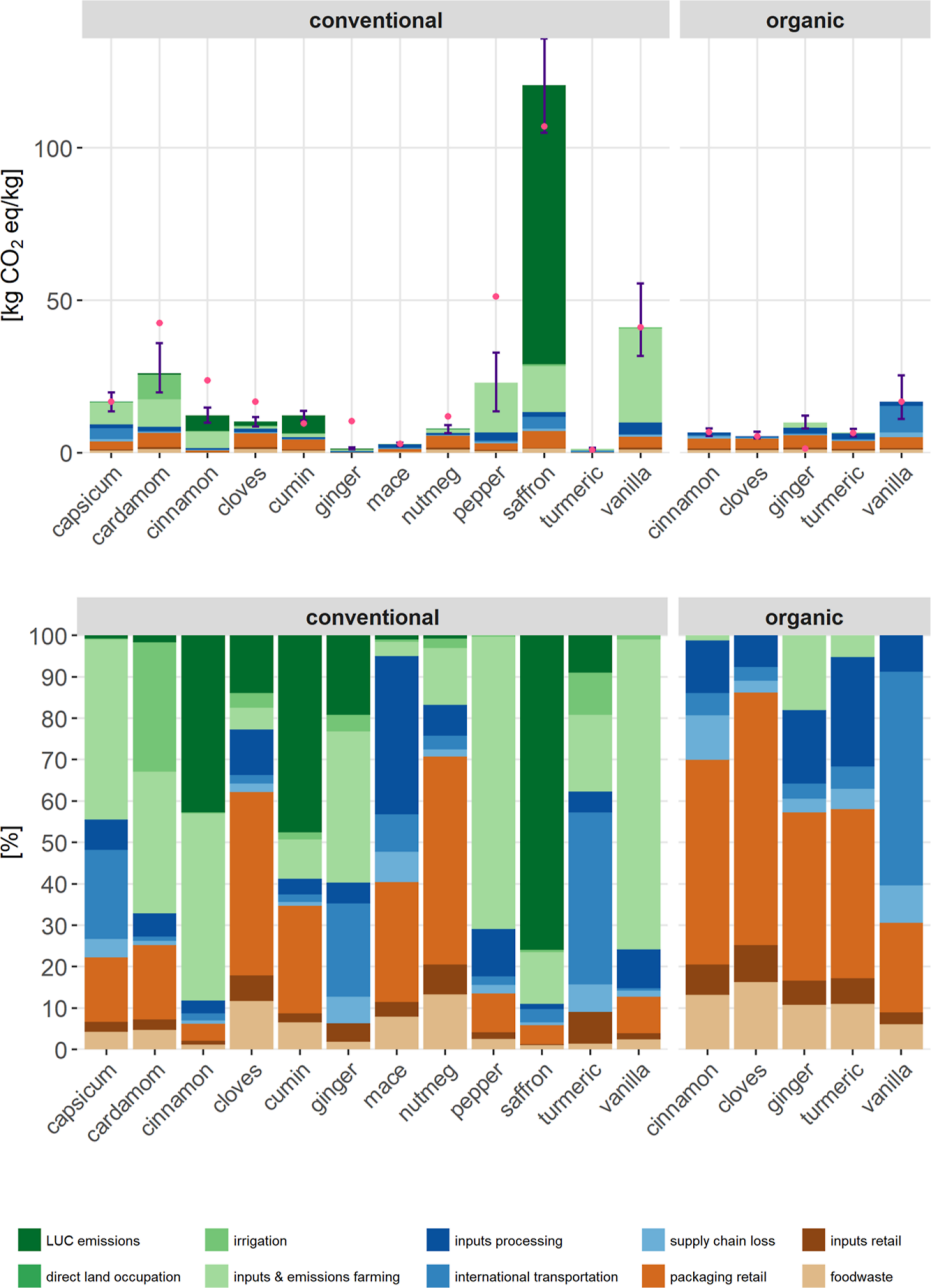
Climate impact of 17 spices based on specific case studies (bars) and converted with global median yield (pink dots) in absolute values (top) and relative contribution (bottom). Error bars represent the upper and lower 95% quantiles derived from the Monte Carlo simulation. The process contributions are shown based on three stages: (1) cultivation: land use change emissions, occupation, irrigation, inputs and emissions farming (fertilizer, pesticides, seeds, cultivation methods); (2) processing: inputs processing (infrastructure, processes like drying), international transportation, supply chain loss; (3) retail: packaging retail, inputs retail (infrastructure, etc.), foodwaste. Conventional turmeric and ginger are modeled as fresh rhizomes at the point of sale.

Figure 2 (top) shows the error bars representing the upper and lower 95% quantiles derived from the Monte Carlo simulation. This uncertainty arises mainly from variability in cultivation practices that are not fully captured in the available data sources, as well as from yield variability.

Conventional spices tend to have a higher share of environmental impacts originating from the cultivation stage than organic spices. With lower cultivation-related impacts, the environmental impact of organic spices is more influenced by processing and retail. Previous studies have confirmed that organic cultivation often results in lower environmental burdens than conventional methods.^60,62^ This is primarily due to the prohibition of synthetic pesticides and mineral fertilizers, which removes some of the most environmentally intensive inputs. According to international organic standards, these substances are strictly banned.^63^ However, lower organic yields can also lead to higher climate impacts.^64^ Therefore, caution is required when comparing organic and conventional products, as the differences often reflect the characteristics of the cultivation systems (see Chapter [Sec sec4.4]).

### 3.2 Biodiversity Footprint per kg of Spice

The biodiversity intensities per kilogram of spices were calculated using two different methods. Using the LUIF method (Figure 3), organic vanilla shows the highest potential species loss per kilogram, estimated at 2.95 × 10^–12^ PDF·yr/kg. Applying LC-IMPACT_updated_ with the updated land-use method (LUIF) (Figure 4) results in the highest biodiversity intensity for conventional vanilla (4.0 × 10^–12^ PDF·yr/kg), followed by saffron (3.5 × 10^–12^ PDF·yr/kg) and cardamom (5.5 × 10^–13^ PDF·yr/kg). The LUIF method produces lower biodiversity impact estimates than LC-IMPACT_updated_ because it considers only land use, whereas LC-IMPACT_updated_ also factors in other pressures such as water use and global warming.

**Figure 3:**
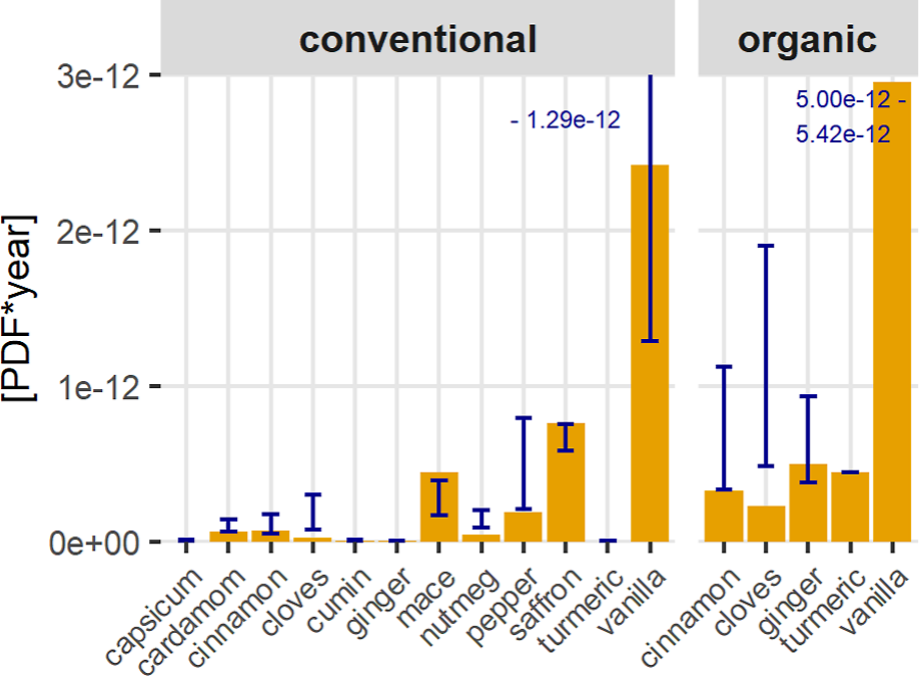
Biodiversity impact of all spices assessed with the LUIF method in absolute PDF·yr per kilogram of spice. The blue sensitivity bars show the interquartile-based sensitivity analysis using the 25th and 75th percentiles of the yield distribution.

**Figure 4:**
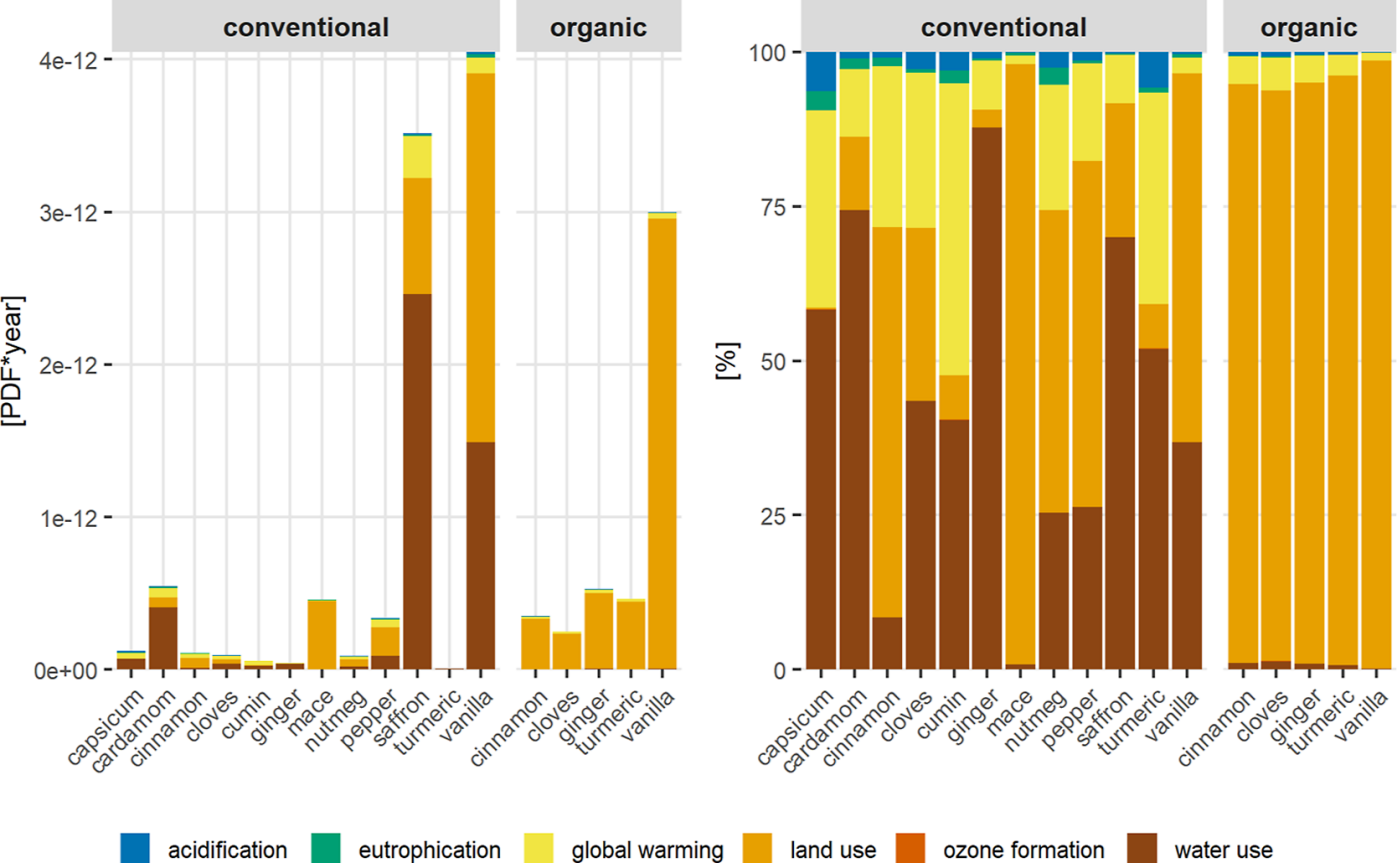
Biodiversity impact of spices assessed with the LC-IMPACT_updated_ method in PDF·yr per kilogram of spice (left) and relative contributions (right) with contributions per impact category.

The LUIF method includes two primary factors influencing the result. The first is the characterization factor, which depends on the region and the biodiversity present in that area, the land-use type, and the intensity level of cultivation. The second factor is the land area required to produce 1 kg of spice. Together, these factors play a significant role in determining the environmental impact of spice cultivation: spices requiring large land areas (e.g., saffron, vanilla, mace, cardamom, and pepper) or those grown in biodiversity-rich regions (e.g., Madagascar, the Central Highlands in Vietnam, Kerala in India) exhibit higher biodiversity impacts. Although organic vanilla requires less land (33.3 m^2^/kg) than conventional production (168 m^2^/kg), its cultivation in Madagascar leads to higher biodiversity impacts due to many endemic species in the area. Saffron requires the highest land use, but its biodiversity impact is lower due to cultivation in Iran’s Khorasan region, which is less biodiverse than other spice-producing areas,^57^ resulting in 7.6 × 10^–13^ PDF·yr per kilogram.

As shown in Figure 7, yield variability is high. To account for this, an interquartile sensitivity analysis was conducted using the 25th and 75th percentiles of the yield distribution. The resulting variability in PDF·yr is shown as blue sensitivity bars in Figure 3. This illustrates how strongly the results depend on yield: higher yields reduce PDF·yr per kilogram, whereas lower yields increase it. These differences stem from diversity in cultivation practices, ranging from spice as a side crop to highly optimized systems targeting maximum yields (SI1, Chapters 1–11). An additional Monte Carlo simulation assessing uncertainty in the LC-IMPACT_updated_ results revealed substantial uncertainties (SI6, SI1 Figure 16).

Differences between the LUIF method and the LC-IMPACT_updated_ method become evident when comparing the relative contributions of impact categories to the overall biodiversity footprint. LUIF focuses exclusively on direct biodiversity impacts from land use, which are determined by factors such as occupation area, regional biodiversity, land-use type, and management intensity. In contrast, LC-IMPACT_updated_ accounts for both direct and indirect drivers of biodiversity loss. In addition to land use, it incorporates further impact pathways such as acidification, eutrophication, global warming, ozone formation, and water use and their effects on ecosystem quality. No consistent pattern emerges regarding the dominance of direct versus indirect impacts across all spices (Figure 4). Nevertheless, all organically produced spices exhibit more than 92% of their total PDF·yr attributable to direct impacts (land use). In contrast, several conventionally produced spices, such as capsicum, cardamom, cumin, cloves, turmeric, and ginger, show more than 72% of their total PDF·yr arising from indirect impacts, primarily linked to global warming and water use (SI1, Chapter 21).

### 3.3 Carbon Footprint of the Global Spice Market

The carbon intensities per kilogram of spice do not reflect each spice’s overall impact, which is why the results were scaled using global production volumes. The analysis revealed that the global spice sector emits 52.1 Mt CO_2_-eq annually (Figure 5, total). The 95% upper and lower quantiles of the Monte Carlo simulation for the spice market range from 41.4 to 64.8 Mt CO_2_-eq per year. Capsicum production for paprika spice accounts for the largest share of emissions (40.4% of total impacts), followed by cumin (17.8%), pepper (17.5%), and ginger (13.7%). The error bars in Figure 5 represent the upper and lower 95% quantiles of the Monte Carlo simulation, highlighting differences in uncertainty across the spices. For instance, pepper production contributes 9.12 Mt CO_2_-eq, with an uncertainty range of 5.43–13.0 Mt CO_2_-eq, largely due to variability in plantation poles (wood, concrete, or living plants; see sensitivity analysis SI1, Figure 20). In contrast, cumin contributes 9.28 Mt CO_2_-eq, with a narrower uncertainty range of 8.13–10.5 Mt CO_2_-eq.

**Figure 5:**
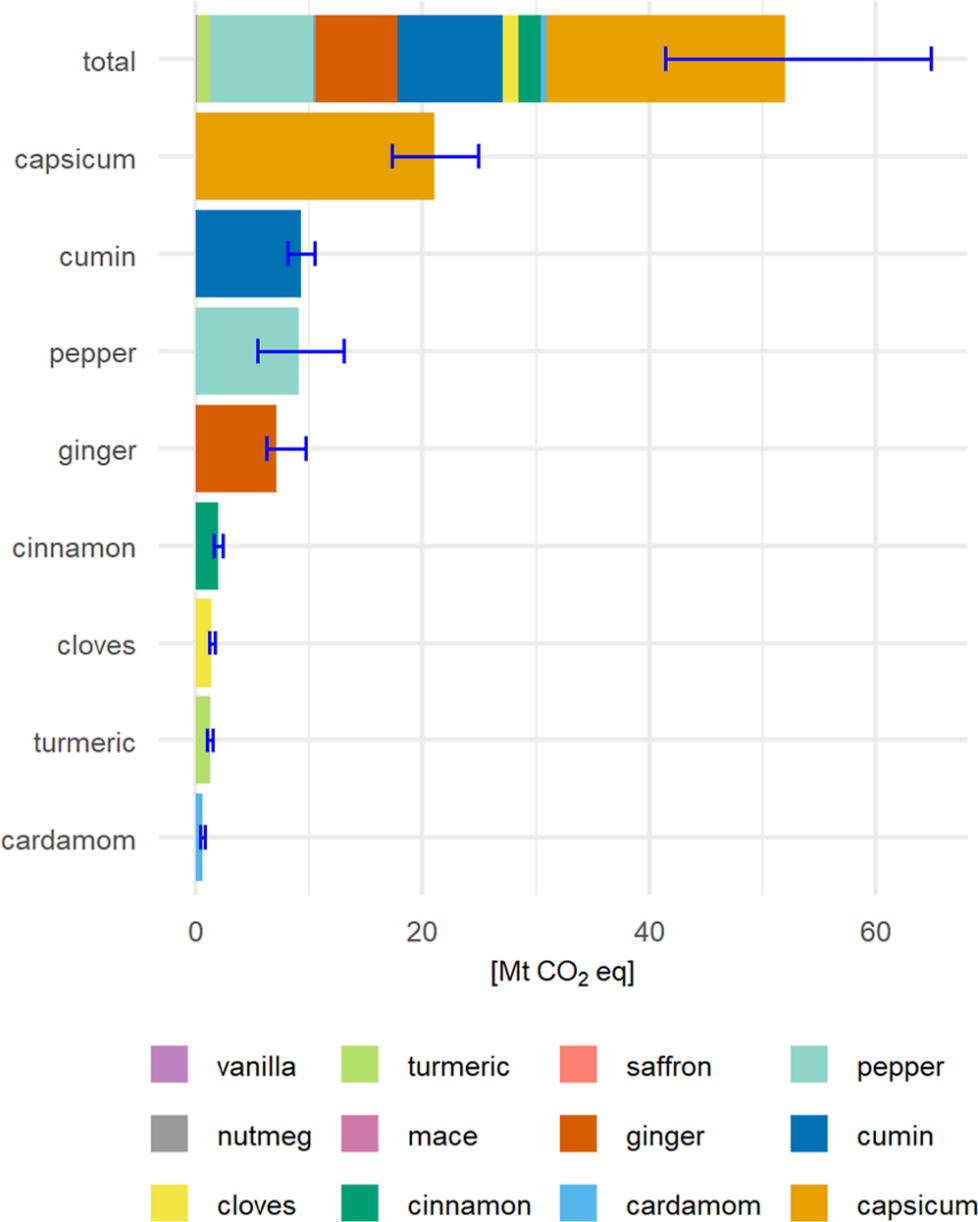
Annual contribution of different spices to the carbon footprint of the global spice market, expressed in absolute values (Mt CO_2_-eq). The stacked bar at the top shows the total climate impact with the proportional contributions of individual spices; the accompanying error bar represents the aggregated upper and lower 95% quantiles derived from the Monte Carlo simulation. Below, individual bars display the most relevant spices in order of decreasing contribution, each with its respective upper and lower 95% quantiles.

Overall, spices produced in large volumes with high environmental intensities dominate the sector’s climate impact. This emphasizes the importance of considering both production scale and per-unit intensity when assessing environmental burdens of whole sectors.

### 3.4 Biodiversity Footprint of the Global Spice Market

In terms of biodiversity footprint, the spice sector’s impact caused by land use, calculated with the LUIF method, is 2.9 × 10^–4^ PDF·yr. However, when indirect drivers are also included using LC-IMPACT_updated_, the result increases to 7.2 × 10^–4^ PDF·yr. To illustrate variation based on yield in the LUIF method, a sensitivity analysis using the interquartile range of yield was conducted (Figure 3). Based on this assessment, the interquartile-derived uncertainty range for the global spice market spans from 2.8 × 10^–4^ to 7.4 × 10^–4^ PDF·yr. The main result lies therefore in the lower part of this range because the case studies use comparatively high yields. As the method comparison indicates, indirect drivers are crucial. Therefore, for the final sector-level analysis, the LC-IMPACT_updated_ method with the updated land-use method (LUIF) was applied. Significant contributors to the global biodiversity impact of the global spice sector are ginger (39.0%), capsicum (21.3%), pepper (15.6%), and turmeric (10.0%). Despite high per-kilogram intensities for spices like saffron and vanilla, their contributions remain small due to low global production volumes. The PDF·yr of the global spice sector is distributed to production countries, spices, and importing regions and is visualized in Figure 6.

**Figure 6:**
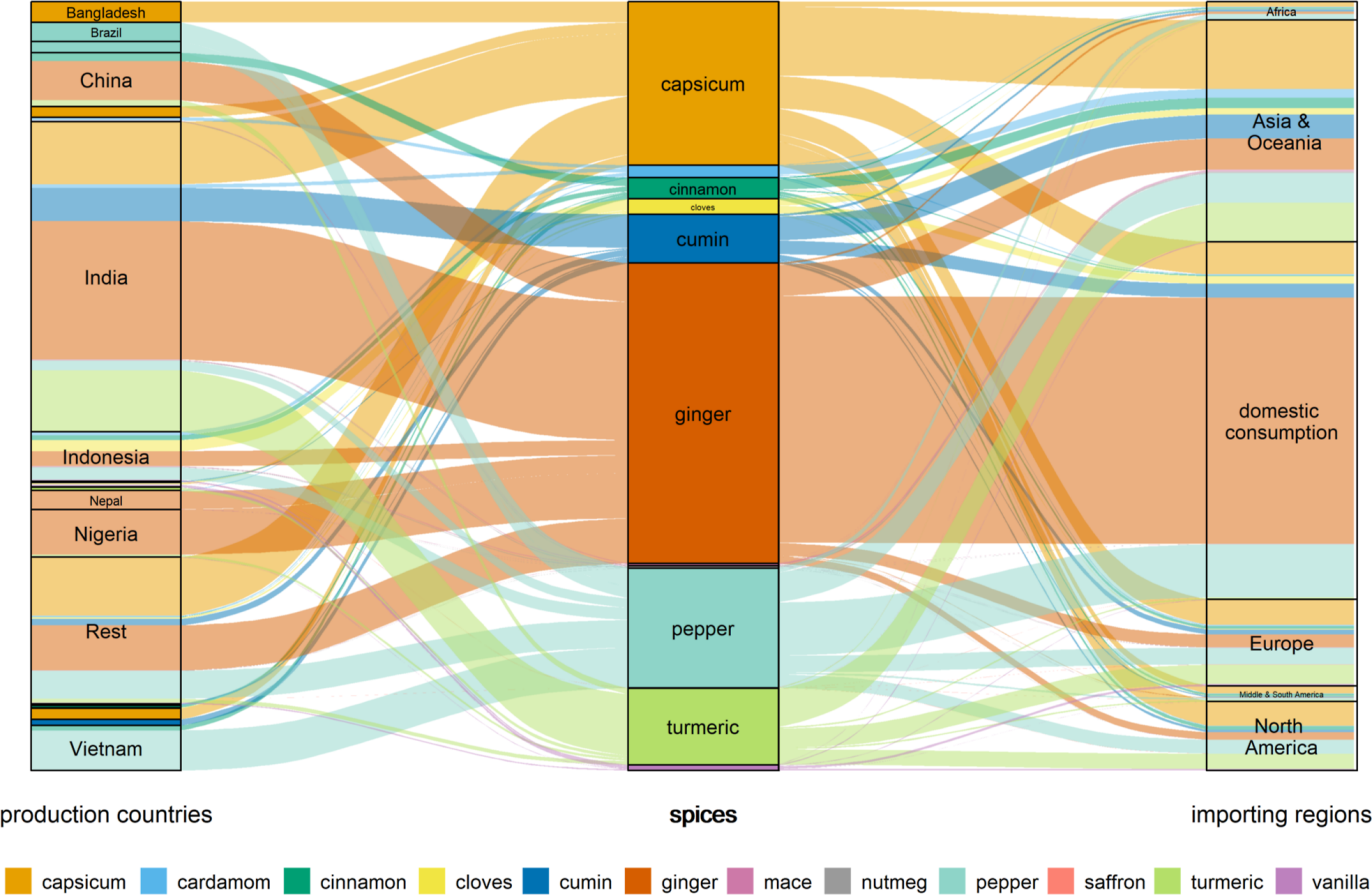
Flow of potentially disappeared fraction of species (PDF·yr), assessed with the LC-IMPACT_updated_ method from production countries (left) per spice (middle) and to importing regions (right).

Most global spice production happens in biodiversity-rich, tropical regions. Among the production countries, India shows the highest biodiversity footprint, accounting for 40.3% of the overall PDF·yr, followed by China (7%), Indonesia (6.5%), Nigeria (6.1%), and Vietnam (5.9%). From a consumption perspective, 46.5% of spices are consumed locally in the production countries. Asian countries are the largest importers of spices (28.8%), mainly sourcing from other Asian countries, followed by Europe (11.3%) and North America (9%).

## 4 Discussion

### 4.1 Embedment in a Global Context

The global spice sector contributes 52.1 Mt CO_2_-eq (lower and upper 95% quantiles: 41.4–64.8 Mt CO_2_-eq), representing 0.325% of the total agrifood sector’s annual GHG emissions. The sector’s turnover of $15.5 billion^65^ corresponds to 0.18% of the agri-food sector’s total turnover ($8.67 trillion),^66^ illustrating the relevance of spices within the agri-food sector. While certain spices, such as saffron and vanilla, command high prices, their overall revenue remains modest due to limited production volumes.

According to the FAO, approximately 9.5 billion tonnes of primary crops are produced globally each year,^2^ while spices account for merely 0.06% (around 5.3 million tonnes). This disparity shows that spices have a disproportionately higher CO_2_-eq per kilogram compared with average crops, resulting in an environmental impact that is 5.4 times greater relative to their production.

For context, the carbon footprints of vegetables and fruits typically range from 0.1 to 7.4 kg CO_2_-eq/kg,^67^ substantially lower than that of spices. The CO_2_-eq/kg of spices was found to be 8 to 16× higher than that of other crop categories. These elevated carbon emissions result primarily from the greater number of processing steps, such as drying, and the higher land-use requirements per unit of product compared to most other crops.^68^ The inefficiency arises from several factors: less effective cultivation practices that yield lower outputs, and longer crop life cycles relative to vegetables, which are predominantly annual plants with shorter growing cycles.^68^

However, since spices are consumed in much smaller quantities than vegetables, a direct weight-based comparison overlooks actual consumption patterns; assessing emissions per serving offers a more realistic evaluation of their environmental impact from a consumption perspective. Therefore, an example calculation for a fictional menu is presented. Assuming a menu for one person comprising 250 g of pork, potatoes, and red cabbage results in an estimated carbon footprint of 1.81 kg CO_2_-eq^21^ Adding one teaspoon (4 g) of capsicum and 1/2 teaspoon (2 g) of black pepper increases the carbon emissions by 67.2 g CO_2_-eq for the capsicum and 45.8 g CO_2_-eq for the black pepper. Consequently, the total carbon footprint of the meal becomes 1.93 kg CO_2_-eq/menu, representing an increase of 6.2% in environmental impact when spices are included.

The biodiversity footprint of the global spice sector, including both direct and indirect impacts assessed with the LC-Impact_uptaded_ method, amounts to 7.2 × 10^–4^ PDF·yr. This aligns with MRIO-based LC-Impact estimates showing substantially larger biodiversity footprints for the overall food system, such as 0.15 PDF·yr for Europe’s food consumption, including 5.2 × 10^–2^ PDF·yr from plant-based foods.^8^ Product-level comparisons also show that tropical perennial export corps tend to exhibit relatively high biodiversity intensities. For example, coffee production has been estimated at 1.15 × 10^–12^ PDF·yr using LC-Impact,^69^ which is within the same order of magnitude as high-impact spices such as conventional vanilla at 4.0 × 10^–12^ PDF·yr per kg.

When biodiversity loss is assessed considering only land-use-induced impacts, substantially lower values are obtained. For the spice sector, the biodiversity footprint decreases to 2.9 × 10^–4^ PDF·yr, using the LUIF method. An MRIO study using the Chaudhary et al. 2015 ^58^ characterization factors estimated a biodiversity impact of 1.02 × 10^–5^ PDF·yr for total global soybean production.^70^ The higher footprint estimated for spices, despite their smaller production volume, reflects structural differences in production systems. Spice cultivation is largely concentrated in tropical biodiversity hotspots with high species richness and endemism, such as Madagascar, where SAR-based models assign substantially higher characterization factors to land-use change. In addition, many spice crops have relatively low yields, increasing land demand per unit of product and thereby elevating biodiversity impacts per kilogram.

Independent of the used biodiversity method, MRIO-based analyses consistently identify agricultural production systems,^3–5,8^ particularly tropical crops,^3^ as major drivers of global land-use-induced biodiversity loss. Globalized trade and long supply chains shift biodiversity impacts away from the point of consumption,^3,7,71^ creating production hotspots in high-biodiversity regions.^72^ Although sustainability projects in Europe and North America have reduced biodiversity loss associated with land use and LUC, it remains critical to prevent the displacement of these impacts to biodiversity-rich regions.^73^

### 4.2 Literature Comparison

The comparison with existing literature shows that most studies only account for the agricultural stage, which can significantly underestimate the overall environmental impacts of spices. A Vietnamese study on cinnamon reported lower GHG emissions of 2.14 kg CO_2_-eq/kg as it considered only cultivation and processing.^74^ The higher emissions in this study (organic: 6.8 kg CO_2_-eq/kg, conventional: 12.3 kg CO_2_-eq/kg) are mainly due to the inclusion of the retail stage, with packaging alone accounting for 69% of organic cinnamon’s total impact. Without packaging, the GHG emissions of the organic cinnamon are 2.11 kg CO_2_-eq/kg, aligning with the Vietnamese study.

Khanali et al.^18^ reported 1′816 kg CO_2_-eq/ha saffron, driven mainly by fertilizer use. In contrast, the 121 kg CO_2_-eq/kg (420 kg CO_2_-eq/ha) found in this study is primarily driven by LUC (75.9%), highlighting the major influence of land management on saffron’s GWP. A more recent study from 2021 of the Khorasan region reported 339 kg CO_2_-eq/kg flower yield.^75^ The lower values observed in our study compared to those reported in the literature can be attributed to reduced electricity inputs, as sun drying was assumed.

The results for organic vanilla (16.8 kg CO_2_-eq/kg) are consistent with the 13.4 kg CO_2_-eq/kg reported for organic vanilla in ecoinvent.^20^ The impact assessment of pepper revealed emissions of 22.9 kg CO_2_-eq/kg, with 70.9% attributed to cultivation, driven primarily by concrete poles (60.1%). This contrasts with Korean studies,^19^ which reported much lower emissions for red (4.13 kg CO_2_-eq/kg) and green pepper (4.7 kg CO_2_-eq/kg). Their results were dominated by fertilizer use,^19^ likely because concrete poles and retail processes were excluded.

### 4.3 Comparison of Biodiversity Assessment Methods

While land use is identified as the most dominant driver of biodiversity loss for spices, focusing solely on land use risks underestimating total biodiversity impacts.^71^ The results of the LC-IMPACT analysis show that land use is indeed the most important impact category for most spices; however, indirect drivers are more significant for certain spices. Therefore, using a method that integrates indirect impacts is crucial.

However, LC-IMPACT_original_ relies on the Chaudhary et al.^58^ model for land-use assessment, which represents an earlier version of the current LUIF method. The LUIF method developed by Scherer et al.^57^ applies a more advanced model that retains ecoregion-level resolution and additionally accounts for fragmentation effects and taxonomic differentiation. LUIF is selected for its advanced, regionalized approach, providing reliable land-use-induced results by considering regional biodiversity and endemic species through elementary flows. The LUIF method is considered state of the art and is included in the most recent global guidance of life cycle impact assessment method (GLAM) release.^76^

Since land use is a dominant factor influencing biodiversity in LC-Impact, accurate land-use data is crucial for interpreting outcomes. To improve accuracy, this study applies a combined approach, integrating LC-IMPACT_original_ with the LUIF method. In this study, the land-use characterization factors from Scherer et al. (2023) were applied in place of the original LC-IMPACT land-use factors (based on Chaudhary et al., 2015), while all other biodiversity-related impact categories were assessed using LC-IMPACT. Although both approaches rely on SAR-based modeling and report impacts in PDF·year, they differ in ecological parametrization, particularly regarding vulnerability scaling and the explicit inclusion of habitat fragmentation. Consequently, the aggregated biodiversity results represent a hybrid modeling framework not fully harmonized for extinction modeling approach across all biodiversity pressures.

Both methods can be compared based on their biodiversity results (PDF·yr). The global potentially disappeared fraction of species (PDF·yr) indicates the fraction of global species committed to extinction due to anthropogenic pressures such as land use. For example, a PDF of 0.01 corresponds to 1% of the global species pool potentially committed to extinction if the pressure persists.^59^ Species do not go extinct instantly after a pressure is applied, because there is typically a lag time between the increase in pressure and its ecological effects. In other words, changes in land use do not immediately cause global extinctions but affect species gradually over time. This is why the duration of exposure to a pressure is included in the unit “PDF·yr”. Accordingly, impact scores reflect the increase in global extinction risk over the period of exposure, rather than immediate species loss.

The primary drivers of biodiversity loss identified by IPBES,^77^ such as land use change, climate change, and environmental pollution, are incorporated in the combined approach using the LUIF method and LC-IMPACT_original_. However, other key biodiversity drivers, such as exploiting organisms and invasive species,^77^ are not yet comprehensively represented in commonly used LCA methods, including those applied in this study. Emerging methodological developments are beginning to address these stressors. For example, recent approaches consider the impacts of terrestrial alien species when they are accidently introduced to new areas, where their potential effects on biodiversity can be even more severe than those of climate change.^78^

In conclusion, no single approach can comprehensively capture all elementary flows, pressures, ecosystems, and taxonomic groups at a regionalized ecoregion level with high reliability.^79^ However, the methodological combination of LC-IMPACT_original_ and LUIF to LC-IMPACT_updated_ offers a holistic assessment of biodiversity impacts at the ecoregion level.

### 4.4 Uncertainty and Limitations

While individual case data were used, the emphasis of this study lies on a comprehensive sector-wide analysis rather than on individual products. The assessment considered 12 key spices; other spices were excluded due to limited and uncertain production data.

One limitation of this study concerns the treatment of organic production systems and the availability of reliable data on organic production shares. Current LCA methodologies do not fully capture the benefits of organic cultivation, as they primarily focus on harvested products and often overlook the multifunctionality of organic systems. This narrow scope excludes important noncommodity outputs and ecosystem services such as pollination, natural pest control, erosion regulation, or water purification. As a result, conventional LCA frameworks tend to underestimate the positive externalities of organic farming, leading to potentially biased evaluations.^80^ Because these cobenefits are not accounted for, organic systems often appear to have higher environmental impacts, primarily due to lower yields.^64^ Consequently, comparisons between organic and conventional systems should be interpreted with caution, as they mainly reflect differences in cultivation intensity rather than the full range of environmental services provided. In this study, however, organic spices performed better than their conventional counterparts. This result should not be generalized, as the data were based on a single organic producer using efficient, high-yield management practices with minimal inputs. A meta-analysis on horticultural crops, including spices, reported that organic yields are on average 10–32% lower than conventional yields, though there is substantial variation across production contexts.^81^ In addition, many smallholder farmers employ low-input or agroforestry-based practices that are effectively organic but not certified, meaning their contributions to the sector are likely underrepresented in global statistics.

The case study approach only reflects one specific region and cultivation system per spice and does not capture within-spice variation. Since impacts were scaled using global production volumes, differences between producing countries are not reflected. To show where biodiversity loss occurs, we distributed the global impact across countries using FAO production data, without adjusting for country-specific practices.

When assessing LUC emissions, additional carbon storage in sustainable systems such as agroforestry is currently not captured. Differentiation is limited to annual versus perennial crops and varying tillage and input intensities.^41^ Therefore, spices grown in intercropped or agroforestry systems do not receive reduction in LUC emissions (see sensitivity analysis SI1 Figure 15 & Table 29).

The most significant uncertainty is yield variability, illustrated in the boxplots in Figure 7, which show the yield distributions across all analyzed spices. Agricultural products typically exhibit high yield variation,^67^ and most spices display heterogeneous yield patterns. Turmeric’s interquartile range demonstrates especially high variability, with yields ranging from 4950 kg/ha to 31,300 kg/ha. Organic yields used in the case studies, based on primary data, are higher for cinnamon, cloves, and vanilla compared to the median yield of FAOSTAT.^10^ The conventional yields used for the case studies fall within the interquartile range, except for pepper and cumin. Yield estimation for cardamom, nutmeg, mace (HS0908), and cumin (including anise, badian, and coriander) is challenging due to limited data availability. The modeling relies on case-specific yield data, as shown in the graph by colored dots (green for organic, pink for conventional). To address this high uncertainty, environmental impacts based on the median yields were calculated (Figure 2 top), and the blue bars in Figure 3 show the interquartile based sensitivity analysis using the 25th and 75th percentiles of the yield distribution.

**Figure 7:**
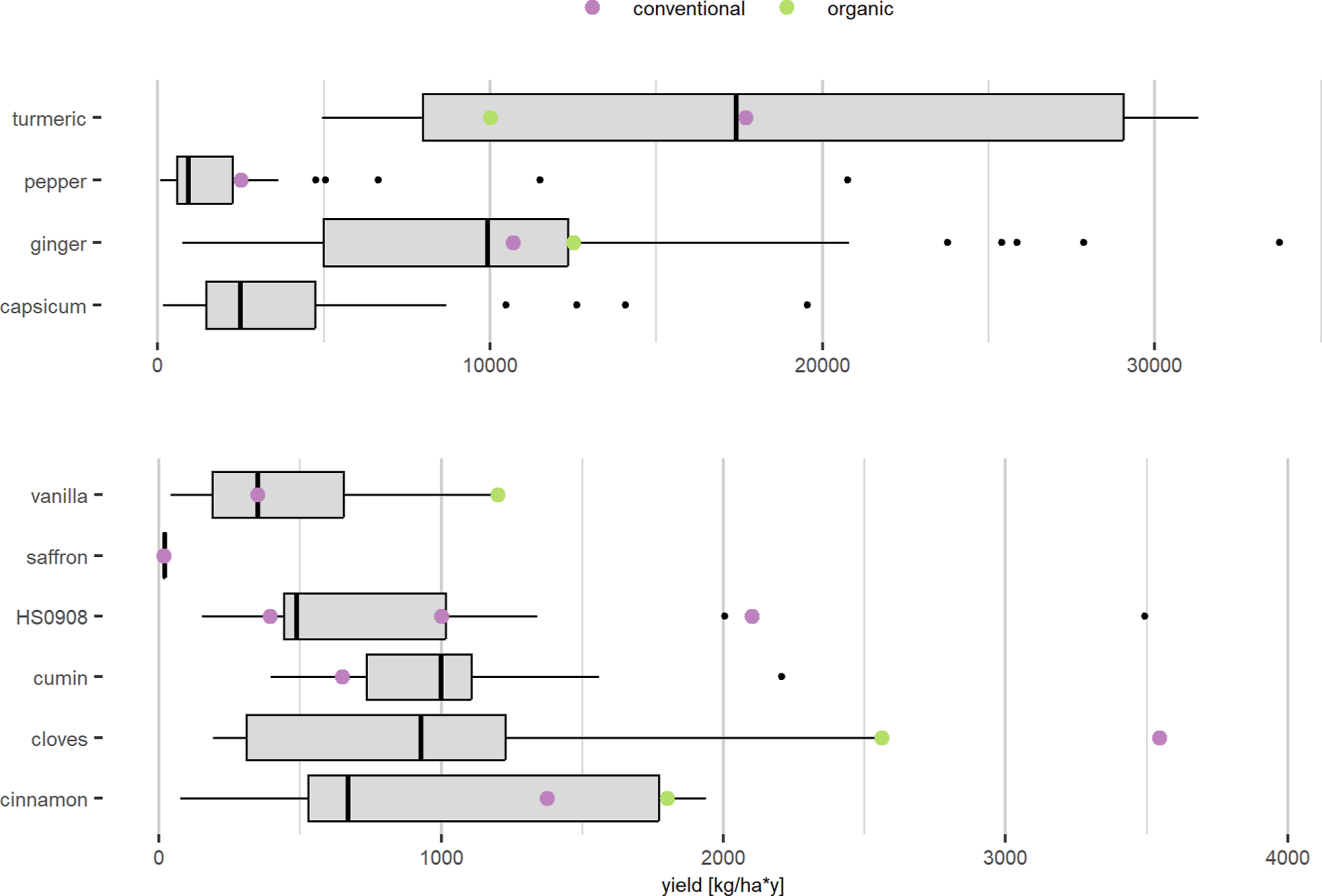
Boxplots of the yield for all analyzed spices.^3^ The line within the box marks the median yield, the bars show the lower (Q1) and upper (Q3) quartiles and 50% of the values are within the box (interquartile range). Outliers are shown as dots. The actual yield values used for the models are colored green (organic cultivation) and purple (conventional cultivation). HS0908 is a summary of cardamom, nutmeg and mace, as yield data only exists for them as one category. Actual yield for capsicum is an outlier (50,705 kg/ha).

To further account for the uncertainty of yield, the case-study approach, and assumptions made in the modeling, a Monte Carlo simulation was carried out, showing the uncertainty for each spice and for the global spice market. The estimated impacts are associated with high variability and should therefore be interpreted as indicative rather than exact. Consequently, while the results allow for a general understanding of potential environmental impacts, direct ranking or fine-scale comparisons between spices are not statistically robust. Variation arises from multiple sources of uncertainty, with scaling being the largest contributor, followed by yield variability (Figure 7).

The final limitation concerns the choice of the functional unit, which in this study was defined as “the total amount of spices supplied to the global market over one year”. From an end-use consumption perspective, a more appropriate functional unit could be a single serving. However, as this study focused on global production, trade, and market-level consumption (i.e., consumption for sale) rather than end-use consumption patterns, this approach was beyond its scope. Future research could apply a single-serving functional unit to capture consumer-level impacts more accurately.

### 4.5 Data Quality

Irrigation and packaging data are robust across all spices. Packaging data are field data based on weighted samples of spice packaging types, while irrigation data followed the WFLDB guidelines^40^ and were adjusted according to country precipitation. The five organic models rely on high-quality primary data provided by an organic producer, though uncertainties arise in the drying and milling stages, where general electricity and infrastructure models are assumed.

The data quality for conventional spices depends on the quality and range of literature values found. Generally, the model of every spice can be differ substantially depending on whether it is cultivated in a monoculture with high input levels or more regenerative agroforestry systems. Additional uncertainties stem from production data, trade data, trade connections, and LUC emissions. FAOSTAT^3^ production data include reliable figures for some countries but also estimates for others, limiting accuracy. Trade data, primarily from FAOSTAT,^10^ impacts transportation calculations and lacks granularity for spices such as saffron and turmeric. As spices are often not listed separately in economic and statistical data, their general reliability is lower than for more common crops.

### 4.6 Recommendations

The following recommendations aim to guide farmers, supply chain actors, and policy-makers in addressing key environmental hotspots identified in this study. They support aligning spice-sector practices with broader sustainability goals, including SDG 12 ^22^ and the EU Deforestation Regulation.^16^

- Sustainable cultivation practices: organic cultivation systems show that reducing mineral fertilizers and fossil fuel machinery can cut climate and biodiversity impacts. Farmers can maintain yields through improved crop varieties, organic amendments, soil health management, and agroecological practices. Redirecting subsidies toward low-input systems aligns with the EU Farm to Fork Strategy,^23^ the global biodiversity framework,^21^ and international climate targets.
- Transport efficiency: shortening transport distances or using lower-impact methods, like minimizing air freight for high-impact spices such as saffron and vanilla, reduces emissions. The EU Green Deal’s goal of a 90% reduction in transport GHG emissions supports modal shifts, infrastructure investment, and low-emission trade policies.^82^
- Low-impact packaging: using packaging with smaller environmental footprints, such as plastic or paper over glass, can significantly cut impacts. For nutmeg, replacing a glass jar with a plastic bag reduces packaging impact by 83.9–96.8% across categories (SI1, Figure 19). Retailers should assess packaging using life-cycle criteria.
- Targeting high-impact spices: sector-wide reductions can be achieved by prioritizing the decarbonization of ginger, pepper, cumin, and capsicum, which together account for the largest share of global spice production and associated environmental impacts.
- Deforestation-free sourcing: spices should not be sourced from areas at risk of deforestation, especially biodiversity hotspots. Instead, supply chains should prioritize regions with low land-use change pressures. Measures such as protected area designations, minimum conservation-to-cultivation ratios, and no-conversion commitments can support alignment with the EU Deforestation Regulation.^16^

In terms of methodological recommendations, the inclusion of the LUIF method into the LC-IMPACT_original_ method should be further explored. The integration and application of land-use transformation and occupation require additional investigation to ensure coherent application without double counting or excessive time-horizon sensitivity. Moreover, the integration of noncommodity outputs in organic or extensive systems should be reflected in biodiversity assessment methods. Additionally, more accurate data on trade flows, production volumes and cultivation inputs are required to enable a more precise assessment of the environmental impacts of the global spice sector.

This study not only provides the first comprehensive analysis of the environmental impacts of the global spice sector but also highlights its underrated relevance within the food sector. It demonstrates that despite the low production volume of spices, their environmental impacts, especially their life cycle greenhouse gas emissions and biodiversity loss, are disproportionately high compared with other crops.

## Supplementary Material













## Notes

A listing of the contents of each file supplied as Supporting Information is provided below.
SI1: Information on the inventory analysis, including production systems of all spices. Information on packaging, transportation, and irrigation. Further visualizations (PDF)
SI2: Excel file “spices_data” with all inventory data on every spice (XLSX)
SI3: Excel file “spices_emissions_calculation” with all emissions from land use change, fertilizer, and pesticide application of all spices (XLSX)
SI4: Excel file “yield_data” with yield, production, and trading data (XLSX)
SI5: Excel file “biodiversity_data” with all results and calculations of the three methods used for biodiversity assessment (XLSX)
SI6: Excel file “monte_carlo_data” with the results of the Monte Carlo simulations for all 17 spice models (XLSX)
