# Supplementary material for: From Farm to Flavor: Carbon and Biodiversity Footprint of the Global Spice Market
Source: Environ Sci Technol. 2026 Apr 7;60(15):11421–33. doi: 10.1021/acs.est.5c04846 (PMC13104020; doi:10.1021/acs.est.5c04846)
Supplement: Supplementary file 1 [file es5c04846_si_002.pdf]

Supporting Information for the manuscript:
From Farm to Flavor: Carbon and Biodiversity Footprint of the Global Spice Market

Authors:
Corinna Bolliger*, René Itten, Matthias Stucki

Affiliation:
Zurich University of Applied Sciences (ZHAW), Institute of Natural Resource Sciences, Life Cycle Assessment Research Group, Grüentalstrasse 14, 8820 Wädenswil, Switzerland.

Corresponding Author:
Corinna Bolliger
Email: corinna.bolliger@zhaw.ch

A listing of the contents of each file supplied as Supporting Information

SI1: Information on the inventory analysis, including production systems of all spices. Information on packaging, transportation, and irrigation. Further visualizations.

SI2: Excel file «spices_data» with all inventory data on every spice.

SI3: Excel file «spices_emissions_calculation» with all emissions from land use change, fertilizer, and pesticide application of all spices.

SI4: Excel file «yield_data» with yield, production, and trading data.

SI5: Excel file «biodiversity_data» with all results and calculations of the three methods used for biodiversity assessment.

SI6: Excel file «monte_carlo_data» with the results of the Monte Carlo simulations for all 17 spice models.

Supporting Information 1

1. Capsicum ... 1
1.1. General Information ... 1
1.2. Inventory data ... 1
1.3. Production system ... 2
2. Cardamom ... 3
2.1. General Information ... 3
2.2. Inventory data ... 3
2.3. Production sytem ... 4
3. Cinnamon ... 6
3.1. General Information ... 6
3.2. Inventory data ... 6
3.3. Production system ... 7
3.3.1. Conventional ... 7
3.3.2. Organic ... 8
4. Cloves ... 10
4.1. General Information ... 10
4.2. Inventory data ... 10
4.3. Production sytem ... 11
4.3.1. Conventional ... 11
4.3.2. Organic ... 13
5. Cumin ... 14
5.1. General Information ... 14
5.2. Inventory data ... 14
5.3. Production system ... 15
6. Ginger ... 16
6.1. General Information ... 16
6.2. Inventory data ... 16
6.3. Production sytem ... 17
6.3.1. Conventional ... 17
6.3.2. Organic ... 19
7. Nutmeg & Mace ... 21
7.1. General Information ... 21
7.2. Inventory data ... 21
7.3. Production sytem ... 22
8. Pepper ... 24
8.1. General Information ... 24
8.2. Inventory data ... 24
8.3. Production sytem ... 25
9. Saffron ... 28

9.1. General Information 28
9.2. Inventory data 28
9.3. Production sytem 29
10. Turmeric 32
10.1. General Information 32
10.2. Inventory data 32
10.3. Production sytem 33
10.3.1. Conventional 33
10.3.2. Organic 35
11. Vanilla 37
11.1. General Information 37
11.2. Inventory data 37
11.3. Production sytem 38
11.3.1. Conventional 38
11.3.2. Organic 39
12. Packaging for transport retail 41
13. Transport distances 44
14. Irrigation 47
15. Biodiversity assessment by the method Scherer et al. (2023) 48
16. Visualisations EF3.1 49
16.1 Environmental impacts of spices (EF3.1) - normalized 49
16.2 Environmental impacts of spices (EF3.1) – absolute values including Monte Carlos Simulation.50
17. Results Biodiversity impact LUIF and LC-Impact & Monte Carlo Simulation 51
18. Production and Economic Share 53
19. Sensitivity Analysis 54
20. Yield of all Spices 56
21. Biodiversity method comparison 57
22. Biodiversity method comparison ....**Error! Bookmark not defined.**
Literature 59

List of Figures

Figure 1: production system of 1 kg dried & ground capsicum sold in global supermarkets 2024 2

Figure 2: production system of 1 kg dried & whole cardamom sold in global supermarkets 2024. 4

Figure 3: production system of 1 kg dried (quills & ground) cinnamon sold in global supermarkets 2024 8

Figure 4: production system of 1 kg dried (& ground) cloves sold in supermarkets 2024. 12

Figure 5: production system of 1 kg cumin seeds dried and whole sold in global supermarkets 2024. 15

Figure 6: production system of 1 kg fresh or dried (& ground) ginger sold in global supermarkets 2024. 19

Figure 7: production system of 1 kg dried & while nutmeg & mace sold in global supermarkets 2024. 23

Figure 8: production system of 1 kg dried peppercorns sold in global supermarkets 2024. 26

Figure 9: production system of 1 kg dried saffron sold in global supermarkets 2024. 29

Figure 10: production system of 1 kg fresh or dried (& ground) turmeric sold in global supermarkets 2024. 34

Figure 11: production system of 1 kg fermented vanilla sold in global supermarkets 2024. 39

Figure 12: World regions used for the importing areas. 46

Figure 13:Normalized values of four midpoint impact categories of EF3.1 of 17 spices based on specific case studies. The process contributions are shown based on three stages: (1) cultivation: land use change emissions, occupation, irrigation, inputs & emissions farming (fertilizer, pesticides, seeds, cultivation methods); (2) processing: inputs processing (infrastructure, processes like drying), international transportation, supply chain loss; (3) retail: packaging retail, inputs retail (infrastructure, etc.), foodwaste. Conventional turmeric and ginger are modelled as fresh rhizomes at point of sale. 49

Figure 14: Absolute values of four midpoint impact categories of EF3.1 of 17 spices based on specific case studies (bars) and converted with global median yield (red dots. Error bars show the 95% confidence interval based on the Monte Carlo simulation. The process contributions are shown based on three stages: (1) cultivation: land use change emissions, occupation, irrigation, inputs & emissions farming (fertilizer, pesticides, seeds, cultivation methods); (2) processing: inputs processing (infrastructure, processes like drying), international transportation, supply chain loss; (3) retail: packaging retail, inputs retail (infrastructure, etc.), foodwaste. Conventional turmeric and ginger are modelled as fresh rhizomes at point of sale. 50

Figure 15:Biodiversity impact in PDF*year per kg of spic in absolute values (left) and relative contributions of the pressures (right) calculated with LUIF and LC-Impact. 51

Figure 16: Absolute values of the potentially disappeared fraction of species (PDF·year) per kilogram of spice, calculated using LC-Impact. Error bars represent the 95% confidence interval based on Monte Carlo simulation. 52

Figure 17: Production and economic share of the 12 most relevant spices (FAOstat, 2022; World Customs Organisation, 2022). 53
Figure 18:Sensitivity analysis of land use change emissions contributions of the five organic spices over two different time horizons: 20 and 50 years. 54
Figure 19: Sensitivity analysis of the retail packaging of the spice nutmeg with glass bottle packaging (left bar) and plastic bag packaging (right bar). The environmental impacts of four EF 3.1 midpoint impact categories and the single score EF 3.1 are shown...55
Figure 20: Sensitivity analysis of pillars in the pepper cultivation system. Scenario “concrete” refers to concrete pillars for 2/3 of the pepper plants and 1/3 plants. Scenario “plants” only uses plants, and scenario “trellis” uses 2/3 wooden trellis and 1/3 plants. Pillars’ contribution is marked in orange but still belongs to the farming stage. 55

List of Tables

Table 1: production amount and production countries of capsicum......1
Table 2: inventory data of capsicum......1
Table 3: production amount and production countries of cardamom......3
Table 4: inventory data of cardamom......3
Table 5: production amount and production countries of cinnamon......6
Table 6: inventory data of cinnamon......6
Table 7: production amount and production countries of cloves......10
Table 8: inventory data of cloves......10
Table 9: production amount and production countries of cumin......14
Table 10: inventory data of cumin......14
Table 11: production amount and production countries of ginger......16
Table 12: inventory data of ginger......16
Table 13: production amount and production countries of nutmeg and mace......21
Table 14: inventory data of nutmeg and mace......21
Table 15: production amount and production countries of pepper......24
Table 16: inventory data of pepper......24
Table 17: production amount and production countries of saffron......28
Table 18: inventory data of saffron......28
Table 19: production amount and production countries of turmeric......32
Table 20: inventory data of turmeric......32
Table 21: production amount and production countries of vanilla......37
Table 22: inventory data of vanilla......37
Table 23: packaging material and disposal datasets used from ecoinvent......41
Table 24: Packaging details for retail phase for every analysed spice......42
Table 25: Transport distances in exporting countries (1), international transport (2), and transport within importing countries (3) for every analysed spice......44
Table 26: example calculation of irrigation amounts for the spice ginger from India......47
Table 27: irrigation amount per in m^3 per ha and per kg fresh yield......47
Table 28: Comparison of the results of a biodiversity assessment of Chaudhary et al. (2015) and Scherer et al. (2023). The orange marked numbers show the higher values......48
Table 29: Land use change emissions [t CO2-eq/ha*year] of the five organic spices over two time horizons: 20 and 50 years......54
Table 30: The spice's fresh yield, weight loss, required fresh yield for 1 kg spice at the point of sale and the dried yield......56
Table 31: Share of direct and indirect impact on species loss per spice in %. Green indicates whether direct or indirect impacts are dominating......57

1. Capsicum

1.1. General Information

Pepper (capsicum ssp.) is a vegetable which is consumed fresh but also served as a spice in dried and ground form (Tripodi & Kumar, 2019). It thrives in climates with temperature between 18 and 27°C, with well distributed rainfalls of 600 to 1250 mm per year in several parts of the world (FAO, 2024).

Table 1: production amount and production countries of capsicum.

Production amount	58’622’202 fresh t of chillies and peppers (capsicum) were produced, out of which **4’909320 t** (8.37%) is then dried and used for spices. → 1’295’631 t dried capsicum/chilli
Producing countries	- India (38.2%), Bangladesh (12.7%), Ethiopia (6.7%), Thailand (6.7%), Rest: 35.8% (FAO, 2022)

1.2. Inventory data

Table 2: inventory data of capsicum.

**models**	**capsicum conventional**
country	India
sources	(Apnikheti, 2024; ecoinvent v3.10, 2024; Sharma et al., 2015; Tripodi & Kumar, 2019)
land use change	- greenhouse model : 0.0618055979029<br>- open field model : 0
yield	- greenhouse model: 90’000 kg/ha (0.0625m2a) → 53%<br>- open field model: 6’395 kg/ha (1.56m2a) → 47%<br>- weight loss of 75%<br>- greenhouse model: 22’500 kg/ha<br>- open field model: 1’599 kg/ha<br>- average fresh yield: 50’705 kg/ha<br>- average dried yield: 12’676 kg/ha
occupation of land	- open field model: annual crop, irrigated<br>- greenhouse model: 7 months occupation, greenhouse
fertiliser	- see excel list
pesticides/ fungicides/ insecticides	- greenhouse model: lambda-cyhalothrin<br>- open field model: insecticides like carbendazim, carbofuran, clothianidin, dichlorvos, thiamethoxam and fungicides like chlorothalonil and difenoconazole
corms (seedlings)	- open field: 0.081142019 p/kg<br>- greenhouse model:
irrigation	- greenhouse model: 0.038 m3<br>- open field: 11.8m3
processing steps	- washing & sorting out → 2 l tap water/kg yield dataset<br>- preparation of shreds<br>- blanching in boiling water 3 min → 0.5l tap water/kg yield<br>- draining shreds & drying in dehydrator 58°c → drying dataset<br>- grinding & sieving → milling dataset<br>for all processes: 1.12 mj/kg processing yield
transport mode to importing countries	- transport by lorry within exporting country (1200 km)<br>- transport by sea freight from india to LA (15’000 km)<br>- general distribution in importing country (not refrigerated)
important	- data for farming part is a mix of ecoinvent data (57% greenhouse, 43% open field, both conventional)

1.3. Production system

Farming phase: For the farming part of the life cycle assessment, two datasets of ecoinvent are used and slightly adapted. A global production system of bell pepper in greenhouses (53%) and a global production of bell pepper on an open field (47%) is used, both conventional prodcution systems.

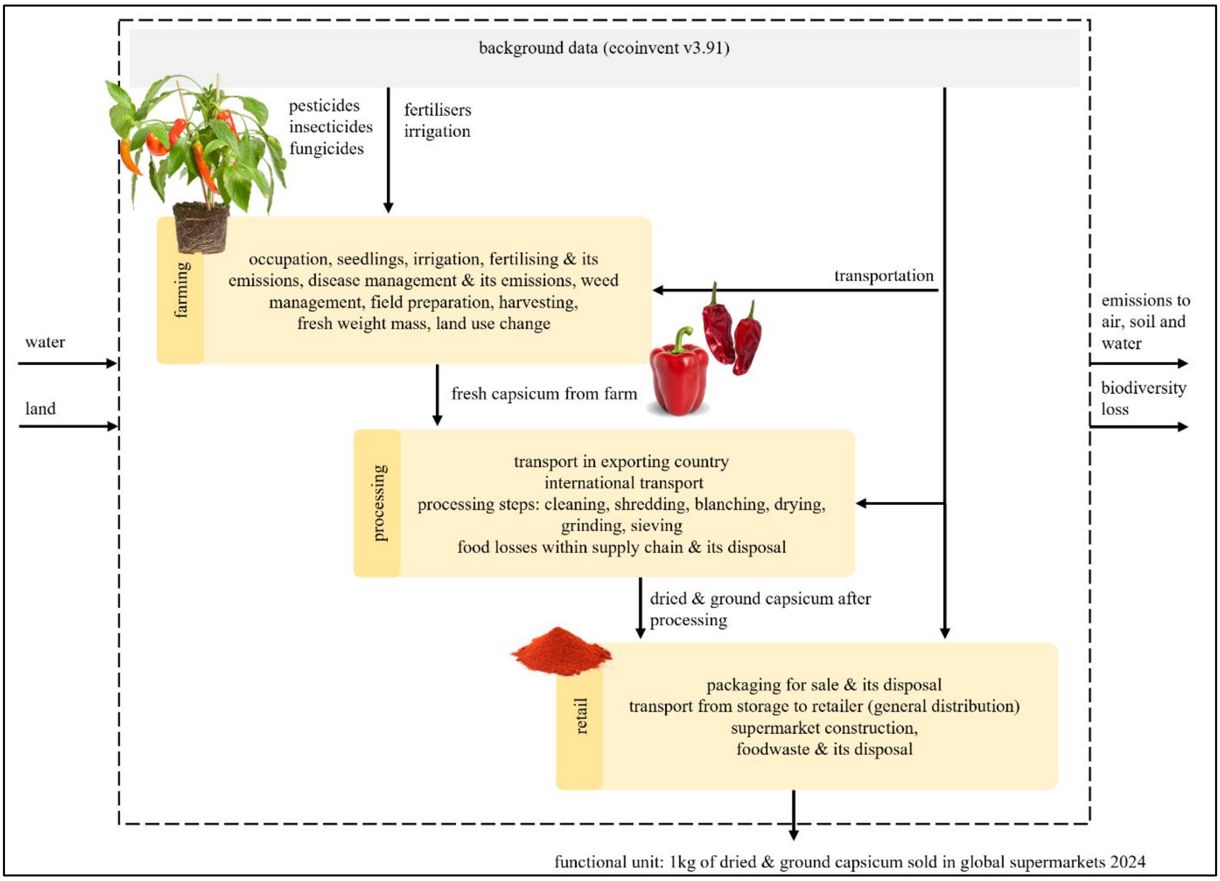

Figure 1: production system of 1 kg dried & ground capsicum sold in global supermarkets 2024

Processing phase: After harvesting, the ripe peppers are washed to remove all dirt, and the bad ones are sorted out (general assumption of water use). After shredding the peppers, they are blanched in boiling water for 3 minutes. After draining the shreds, the peppers are dried in a dehydrator or a solar poly tunnel drier (general assumption of drying). Lastly, the dried peppers are ground (general assumptions of grinding) and sieved before packed into the glasses for sale (Sharma et al., 2015). Additionally, the general assumption for electricity consumption for processing is included. Major importing countries are USA, followed by China, Thailand, and Spain. Therefore, the shipping route is modelled from India, the main producing country, to Los Angeles, the main hub of the importing area (15'000 km).

2. Cardamom

2.1. General Information

Cardamom (*Elettaria cardamomum Maton*) is a spice which uses the dried fruits (capsules) of the cardamom plant. The plant prefers a warm and humid climate with rainfalls between 1500-2000mm, temperatures between 15°C to 35°C and a high humidity between 75% and 90%. The best growing conditions are met in forests with loamy, slightly acidic soils. The harvested pods are either sold as pods or decorticated resulting in seeds. Cardamom powder is the result of grounded seeds and shows an intense flavour which however loses its aroma quickly after grounding. By distillation of the powdered seeds, cardamom oil can be produced. (Ankegowda et al., 2015)

Table 3: production amount and production countries of cardamom.

Production amount	Cardamom, nutmeg and mace yields in 138’887 kg in the year 2022 (FAO, 2022) → 69% cardamom = 96’686 t cardamom → 21’464 t dried
Producing countries	India (29.5%), Indonesia (29.2%), Guatemala (26.2%), Nepal (6.3%), Rest: 8.8% (FAO, 2022)

2.2. Inventory data

The system modelled for this study is an Indian conventional cultivation system:

Table 4: inventory data of cardamom.

models	cardamom
country	India
sources	(Ankegowda et al., 2015; Murugan et al., 2022; Rema Shree et al., 2021; TNAU, 2022)
land use change	20y : 0.1 t CO2eq/ha
yield	- 1000 kg fresh cardamom/ha<br>- Weight loss: 77.8%<br>- 222 kg dried cardamom/ha
occupation of land	perennial
fertiliser	- 50 g rock phosphate per plant/year<br>- 75:75:150 kg/ha, rainfed<br>- 125:125:250 kg/ha, irrigated<br>- FYM 5kg/plant/year
pesticides/ fungicides/ insecticides	- rhizome rot : copper oxychloride 0.25% → 2.5kg/ha (500g/kg active substance) → 1.25 kg active substance/ha<br>- pseudostem rot: carbendazim @ 2 g/L (5 L/plant) at monthly intervals, 3 months → Active substance: 500g/l, application rate: 0.5l/ha = 250g active substance/ha<br>- thrips/ shoot borer/capsule borer/shoot fly: quinalphos/*endosulfan/*fenthion (0.075%) twice during January/February and September/October → active substance 250g/kg, application rate: 250g/ha<br>- herbicide: Paraquat: active substance 438g/kg, application rate: 0.4kg/ha.
seedlings	2500/ha,
irrigation	5334.38 m3/ha, sprinkler irrigation
processing steps	- harvesting → manually<br>- washing → water consumption 2l

	- drying: sun or flue curing → flue curing<br>- polishing → manually<br>- grading<br>- packaging
transport mode to importing countries	- transport by lorry within exporting country (1000 km)<br>- transport by sea freight from India to China (4160 km)<br>- general distribution in importing country (not refrigerated)
important	

2.3. Production sytem

Farming phase: for modelling the seedling, the dataset of wheat seedlings of ecoinvent is used *(Wheat seed, for sowing {GLO}| market for wheat seed, for sowing | Cut-off, U).* It is important to provide shade for the cardamom plants by either cultivating in forests or planting shade trees first. Depending on the variety, the distance between two plants varies. For this model 2500 seedlings/ha are assumed. The yield varies depending on the variety and the region. It lays between 275 kg/ha and 3000 kg/ha (Ankegowda et al., 2015). The horticulture research portal of India reports average yields between 200 and 250 kg/ha (TNAU, 2022). The spice board of India however, shows higher average yield between 600 and 1000 kg fresh cardamom per ha (Rema Shree et al., 2021). For this study, an average of 1000 kg/ha is used. Before and while planting, 50 g rock phosphate and FYM is applied per plant. Cardamom plantations are rainfed or irrigated by drip or sprinkler irrigations systems. NPK fertiliser is applied with following ratio (75:75:150 kg/ha). Additionally, 5 kg of farm yard manure is applied per plant per year. For disease

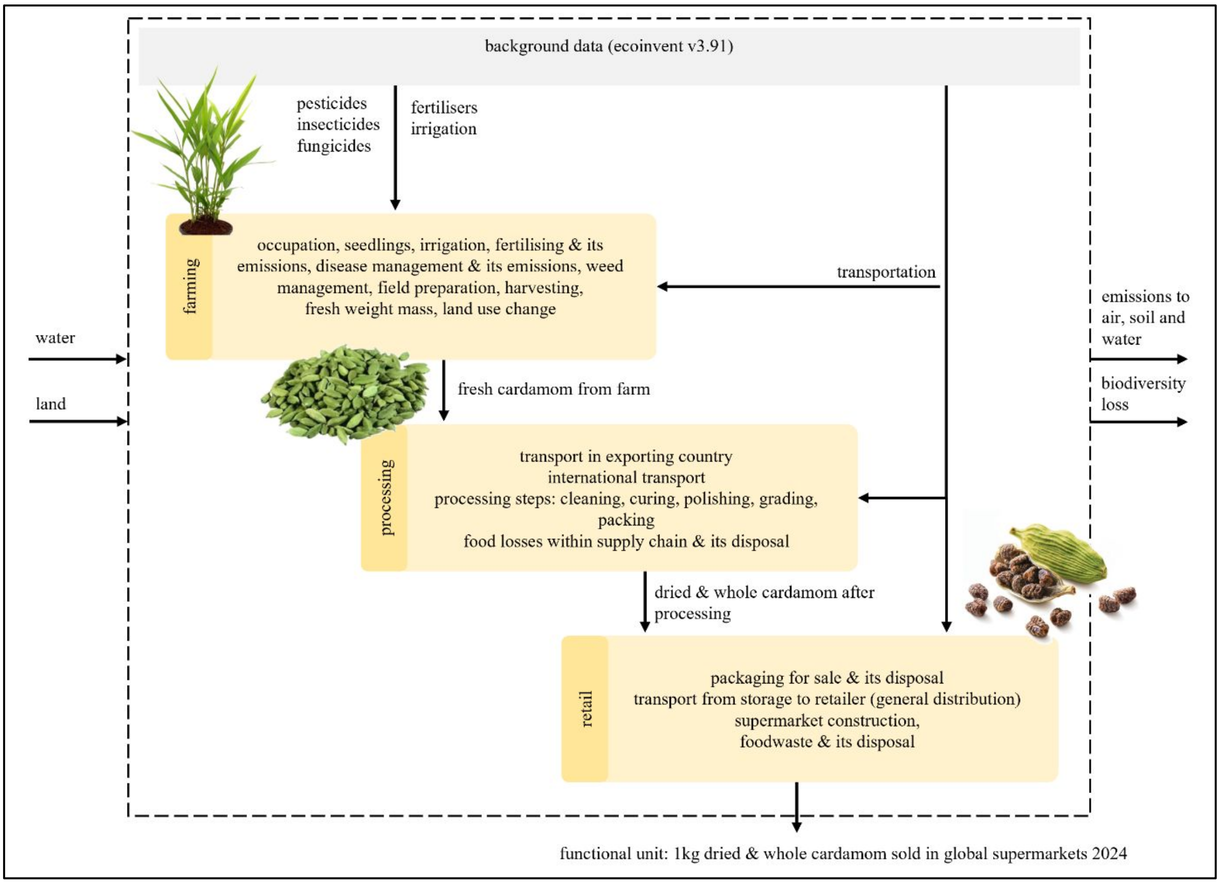

Figure 2: production system of 1 kg dried & whole cardamom sold in global supermarkets 2024.

and weed management, four different chemicals are applied. Against rhizome rot, copper oxychloride is applied (2.5kg/ha, 500g/kg active substance,1.25 kg active substance/ha). For fighting pseudostem rot, ca, carbendazim is applied (5 L/plant, at monthly intervals, 3 months, active substance: 500g/l, application rate: 0.5l/ha = 250g active substance/ha), against thrips, shoot borer, capsule borer, and shoot flies quinalphos, or endosulfan is apllied (twice during January/February and September/October , active substance 250g/kg, application rate: 250g/ha). As a herbicide, paraquat is used (active substance 438g/kg, application rate: 0.4kg/ha). The list of possible pests, viral, and bacterial disease is long but here are only the most occurring once covered since for most of the disease similar or the same pesticides are used. (Ankegowda et al., 2015)

Processing phase: after 2-3 years of plantations, cardamom capsules can be harvested after a growing period of 120-135 days. The harvesting is done manually. To remove dirt and soil particles, the capsules are then washed. In the curing phase, the cardamom moisture level is decreased from 80% to 10% (weight loss of 77.8%). This drying part is either done by natural sun drying or by flue curing. When using sun drying, the capsules lose their green colour and could split. Flue curing however, is the best method to maintain high quality green cardamom capsules and therefore used in this model. The curing traditionally takes place in a drying house (4.5 m in length and a capacity of 2 t) which is heated with a furnace and firewood (3-4 kg firewood/ kg fresh cardamom). By maintaining 45-55°C and even 60°C in the last two hours, the curing process is within 24 hours finished. The next step, called polishing, includes rubbing the dried capsule either by machines or mainly manually. After classification, capsules are packed in 300 gauge black polyethline lined gunny bags which protects their aroma. (Ankegowda et al., 2015)

The main part of the cardamom is consumed in Asia and Arabic countries. China is the leading importer and therefore, the transport is modelled from India to China by sea freight which is 4160 km. Additionally, 1200 km of transport within in India and the general distribution in the importing country is added.

3. Cinnamon

3.1. General Information

Ceylon cinnamon (*Cinnamomum verum*) is the dried inner bark of the evergreen, bushy cinnamomum tree available in quills or as powder, native to Sri Lanka (Britannica, 2024). Related species like Chinese cassia (*Cinnamomum cassia*), Indonesian cinnamon (C. burmannii), or Vietnamese cinnamon (C. loureiroi) are cultivated as cinnamon as well. Cinnamon has a sweet and warm taste and a brown colour (Britannica, 2024). Cassia cinnamon has a less delicated flavour, a thicker bark (more starchy) with a hollow structure, more coumarin and a reddish brown colour in comparison to the true Ceylon cinnamon (Britannica, 2024). The spice is made using the inner barks of the Cinnamomum tree which are dried until they curl into rolls (EDB, 2021). Cinnamon shows anti-viral, anti-fungal, anti-bacterial and anti-carcinogenic properties and is therefore not only used when cooking but also for natural preservative of food and as a medicine or for cosmetics (Persistance Market Research, 2018). If we only take a look at the true Ceylon cinnamon, 90% of it is produced in Sri Lanka (De Silva & Esham, 2020).

Table 5: production amount and production countries of cinnamon.

Production amount	218'694 t of fresh cinnamon was produced in the year 2022 (FAOstat, 2022)<br>164'020 t dried cinnamon
Producing countries	China (40%), Vietnam (24%), Indonesia (23%), Sri Lanka (11%), Madagascar (1.5%), Rest: 0.5% (FAO, 2022)

3.2. Inventory data

Two different case studies are modelled in this study:

1) Organic production in Madagascar with primary data from the organic spice provider, dried and ground
2) Conventional production in India with literature values, dried and quills

Table 6: inventory data of cinnamon.

**models**	**organic model (1)<br>dried & ground**	**conventional model (2)<br>dried & quills**
country	Madagascar	India
sources	organic spice provider, 2024	(H. Aluthgamage et al., 2023; H. N. Aluthgamage et al., 2021; Rawat et al., 2020; Suriyagoda et al., 2021)
land use change	20y: 0, or -4.3 t co2eq/ha<br>50y: 10.6 t co2eq/ha	20y : 5.43 t co2eq/ha
yield	- 1800 kg fresh product/ha<br>- 25 % weight loss & stem pith<br>- 900 kg dried cinnamon/ha	- 1375 kg fresh product<br>- 25 % weight loss<br>- 687.5 kg dried cinnamon/ha
occupation of land	perennial crop	perennial crop
fertiliser	none	- 29kg farmyard manure,

		- 150 g n, 75 g p2o5, 150 g k2o per plant
pesticides/ fungicides/ insecticides	none	endosulphan or quinolphos
seedlings	1000 trees/ha, 100 years expectancy	1100 trees/ha, 100 years expectancy
irrigation	none	290.58 m3/ha (calculated), surface irrigation
processing steps	- debarking, drying, sorting, packing → manually<br>- packaging transport: in plastic bags<br>- packaging sale: glass bottles	- debarking, drying, sorting, packing (manual)<br>- packaging: plastic bags with quills
transport mode to importing countries	- transport by lorry within madagascar (1200 km)<br>- transport by ship to hamburg (17’300 km)<br>- general distribution in import country (not refrigerated)	- transport within india by lorry (1200 km)<br>- transport by ship to la (usa) (23’153 km)<br>- general distribution in import country (not refrigerated)
important	economic allocation	

3.3. Production system

3.3.1. Conventional

Farming phase: Upon reaching the age of four years, the cinnamon tree is viable for harvesting. The optimal time for harvesting is in the wet season in the morning when the branches are still moist which makes their handling afterwards easier. By careful cutting down the branches close to the ground and therefore promoting regrowth, harvesting after eight months is possible again (Business Insider, 2021). One cinnamon tree is planted each 9m2. After three years they are fertilized with 29 kg farm yard manure, 4kg neem cale, 150 g N, 75g P2O5, and 150 g K2O per cinnamon plant (Rawat et al., 2020). To prevent caterpillars of the cinnamon butterfly, 0.05% quinolphos are applied (Rawat et al., 2020). The average yield per ha varies depending on the region. In Sri Lanka its 500 kg dried product/ha, in China bewtween 1350 and 1600 kg/ha, Indonesia 1350 kg/ha (Piyasiri & Wijkeratne, 2016) and sometimes it is also lower like 375 kg/ha (Rawat et al., 2020).

Processing phase: Following the harvest, all branches are transported to processing locations where skilled “peelers” extract the cinnamon (Business Insider, 2021). Initially, nodes and the outer bark are removed with a semicircular blade which exposes the inner valuable bark, the cinnamon (Business Insider, 2021). Rubbing with a brass, helps loosen the bark for harvesting the inner bark (Britannica, 2024). The inner bark is manually stripped with focus on thinness which enhances the value of the final cinnamon. Thinner bark reaches a higher grading and therefore a higher price (Business Insider, 2021). In the case of Ceylon cinnamon, each piece dries in the sun where it scrolls up within minutes. Smaller bark pieces are stuffed within larger rolls to prevent their breakage. Conversely, the thicker bark of cassia cinnamon does not need stuffing. In both cases, the quills (removed bark) are then dried in the shade for three to five days (Business Insider, 2021) which prevents losing their shape. Afterwards the

quill are dried in the sun or with a mechanical dryer to remove any moist (K Agriculture, 2022a). Once dried, the quills undergo a grading, typically based on ten levels which assesses their quality and their value on the market. Factors such as bark's thickness, appearance and essential oil content influence the grading process (K Agriculture, 2022a), with larger and longer quills generally commanding higher prices. Any leftover or smaller parts are used for grounded cinnamon (Business Insider, 2021). Depending on where they are sold and for what purpose, the quills are cut (K Agriculture, 2022). Processing steps like peeling off the bark is labour intensive and all done manually. To sell the quills, normally cut to a length of 10 cm, they need to be stored dry and pest-free and are therefore packed into polypropylene bags for sale. It is important to use moisture-proof bags preventing breaking for transportation (K Agriculture, 2022). The storage conditions for cinnamon are dry, cool, clean, and well-ventilated places preventing direct sunlight (K Agriculture, 2022). And the transport is modelled according to Chapter 13, from India to America, as this is the main importing region (ITC, 2022) and therefore to Los Angeles port(17'300 km).

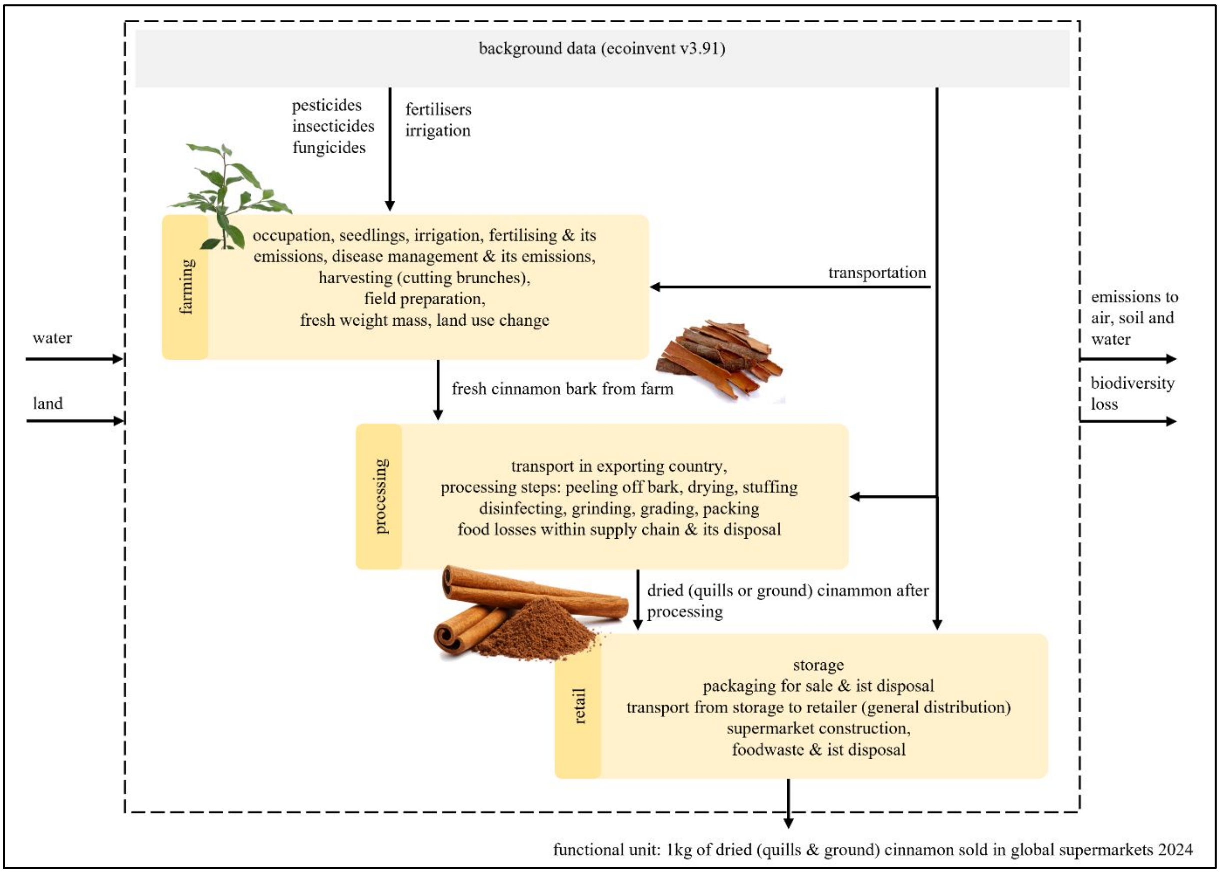

Figure 3: production system of 1 kg dried (quills & ground) cinnamon sold in global supermarkets 2024

3.3.2. Organic

Farming phase: in Madagascar, Cinnamomum zeylanicum, or true cinnamon, is cultivated in an agroforestry system with an average yield of 1800 kg fresh product/ha. This perennial crop grows alongside cassava (7000 kg/ha), pineapple (50,000 kg/ha), litchi (5000 kg/ha), or banana (30,000 kg/ha) with about 1000 trees per hectare. For this, an economic allocation with pineapples is carried out. Since

the exact market price of these products will remain confidential, only the price relation of 1:20 (cassava:cinnamon) is documented. These allocation factors are included in the farming stage, meaning that 72% of the emissions are allocated to ginger, whereas the remaining 28% are allocated to cassava. The Cultivation is entirely manual and organic, with no irrigation, fertilization, or pesticide use.

Processing phase: from the initial harvest (1800 kg/ha), 1200 kg/ha of cinnamon bark is obtained by scraping the stems, while the remaining 600 kg/ha of stem pith is used as firewood. The bark is sun-dried, where it loses 25% of its weight, resulting in 900 kg/ha of dried cinnamon in chips form. It is then packed and transported by lorry to Tamatave and shipped to Hamburg via Colombo. In Hamburg, the chips are stored until they are requested by consumers (assumption of an average storage time of 1 month), either in chips form or as ground cinnamon. In case of pests, pressure disinfection is necessary which is the case in 5% of the amount. If no manifestation with pest, only steam sterilisation (autoklav) is applied with temperatures of 70-105°C. For the ground cinnamon, its ground in a mill in Hamburg wich is cooled. The ground cinnamon is then packaged in glass jars. The jars are subsequently transported and sold in stores.

4. Cloves

4.1. General Information

The unopened flower bud of the cloves tree (*Syzygium aromaticum, Eugenia aromaticum or Eugenia caryophyllata*) serve as a culinary and a medicinal spice (Shamina, 2022). Cloves possesses antioxidant, anti-inflammatory, pain relieving, and anti-funcal properties (Shamina, 2022). Optimal cultivation conditions include a minimal annual rainfall of 100 cm, temperatures ranging from 25°C to 35°C, approximately 70% humidity and partial sunlight exposure (Shamina, 2022). Clove trees thrive in laterite, clay, or loamy soils with high humus content and good drainage to prevent waterlogging (Agri Farming, 2015; India Agro, 2024; Shamina, 2022), often cultivated in intercropping systems alongside cocoa, coconut, or banana to provide partial shade, with bamboo or Jack serving as border crops (Shamina, 2022).

Table 7: production amount and production countries of cloves.

production amount	global production estimated at 183’452.3t (faostat, 2022) → 142’051 t dried
producing countries	- Indonesia (73%), Madagascar (13%), Tanzania (4.7%), Rest: 9.3% (fao, 2022)

4.2. Inventory data

Two different case studies are modelled in this study:

1) Organic production in Madagascar with primary data from the organic spice provider
2) Conventional production in Indonesia and India with literature values

Table 8: inventory data of cloves.

models	organic model (1) ground	conventional model (2) whole cloves
country	Madagascar	India/ Indonesia
sources	organic spice provider 2024	(india agro, 2024; shamina, 2022; agri farming, 2015)
land use transformation	20y: 0 kg /kg fresh cloves<br>50y: 4.14 kg /kg fresh cloves	20y: 0.0266 kg/ kg fresh cloves
yield	- 13.33 kg fresh cloves /tree<br>- 10 kg dried cloves /tree<br>- weight loss of 25%<br>- no yield in first 4 years → 4% less yield<br>- 12.8 kg fresh cloves/tree<br>- 9.6 kg dried cloves/tree<br>- **1920 kg dried product/ha**<br>- **2560 fresh product/ha**	- 13.33 kg fresh cloves /tree<br>- 10 kg dried cloves /tree<br>- weight loss of 25%<br>- no yield in first 4 years → 4% less yield<br>- 12.8 kg fresh cloves/tree<br>- 9.6 kg dried cloves/tree<br>- **2659 kg dried product/ha**<br>- **3546 kg fresh product/ha**
occupation of land	perennial crop	perennial crop
fertiliser	none	15 kg manure, chicken dung or compost

		300 g n (600 g urea), 250 g p2o5 (1560 super phosphate g), 750 g k2o (1250 g muriate of potash) per year and tree
pesticides/ fungicides/ insecticides	none	carbendazim<br>quinalphos<br>dimethoate
seedlings	- 200 trees/ha<br>- life expectancy 100 years<br>- 0.00078 p/kg dried cloves	- 277 trees/ha<br>- life expectancy 100 years<br>- 0.00043 p/kg dried cloves
irrigation	none	8190.45 m3/ha (calculated), surface irrigation
processing steps	- separation of flower from clusters, drying, grading, sorting → manual<br>- Packaging transport: in plastic bags<br>- grinding: machine in Hamburg<br>- Packaging sale: glass bottles	- separation of flowers from clusters (manually)<br>- no grinding<br>- Packaging sale: glass bottles
transport mode to importing countries	- Transport by boat within Madagascar (350 km)<br>- Transport by ship to Hamburg (17'300 km)<br>- general distribution in import country (not refrigerated)	- Transport by ship (3800 km) from indonesia to india<br>- general distribution in import country (not refrigerated)
important		

4.3. Production sytem

4.3.1. Conventional

Farming phase: Ripe seeds of a mother clove are used to cultivate a clove tree (Agri Farming, 2015; Shamina, 2022). These seeds are soaked overnight immediately after harvesting and then sown into pots (Agri Farming, 2015; Shamina, 2022). Germination typically occurs within 15 days, with seedlings nurtured in pots containing a soil mix of sand, decomposed organic matter, and cow dung (Agri Farming, 2015) until they reach 18-24 months of age (India Agro, 2024). Clove trees require about 6 to 7 meters distance between each other, depending on the intercropping culture (Agri Farming, 2015; India Agro, 2024). This means 36 m2 per tree resulting in 277 trees per acre. During the initial four years, considerable care is essential for establishment, with low clove yields during this period. It takes four years until the tree starts flowering, and another six years to reach the full production volume. Once a tree is established, the production will continue for the following 100 years (Shamina, 2022). For accounting the missing yield in the first four years, 4% less yield is taken over 100 years. Yield can vary between 2 kg and 10 kg per plant (India Agro, 2024; Shamina, 2022). For this study also 10 kg like in the organic model are taken.

Irrigation is only necessary in the first four years and in drought seasons (Agri Farming, 2015; Shamina, 2022). Common diseases attacking clove trees are stem borer which causes death by boring into the stem, scale insects which mainly infest the leaves and leaf rot (cylindrocladium quinquiseptatam) which causes defoliation. By spraying a 0.2% carbendazim (India Agro, 2024) or copper sulphate twice a year (Shamina, 2022) on the tree, leaf rot can be mitigated. Stem borer are treated by swabbing the surface

with a 50% w.p.b.h.c (India Agro, 2024) or by using 0.15% carbaryl or 0.1% Quinalphos (Shamina, 2022). Scale insects are killed by applying a spray with the insecticide dimethoate (0.05%) (Shamina, 2022). Fertilising the right amount can play a key role in yield amounts. A combination of 50-60 kg manure, chicken dung or compost in Mai or June with inorganic fertiliser is used (Agri Farming, 2015; Shamina, 2022). If using inorganic fertilizer the recommendation is to use 300 g N (600 g urea), 250 g P2O5 (1560 super phosphate g), 750 g K2O (1250 g Muriate of potash) per year and tree applied in two doses through the year (Agri Farming, 2015).

Processing phase: as soon as the flower buds are turning pinkish red, they can be harvested by handpicking. Optimal cloves exhibit four calyx and four unopened petals, measuring approximately 2cm in length. Yield underlays fluctuation from 2 to 8 kg per tree and year. However, on average, the yield is between 2 and 3 kg per tree and year (India Agro, 2024; Shamina, 2022). Following harvest, meticulous separation of flowers from clusters is performed manually, followed by sun-drying lasting four to seven days. The completion of drying is indicated by the stem's dark brown coloration.

Retail phase: In the conventional model, cloves are sold as whole in glass bottles. Since India is the main importer and Indonesia the main exporter (ITC, 2022), the shipping route between these countries is modelled which is 3800 km by sea freight from Jakarta via Singapore to Chennai in India (Fluent Cargo, 2024).

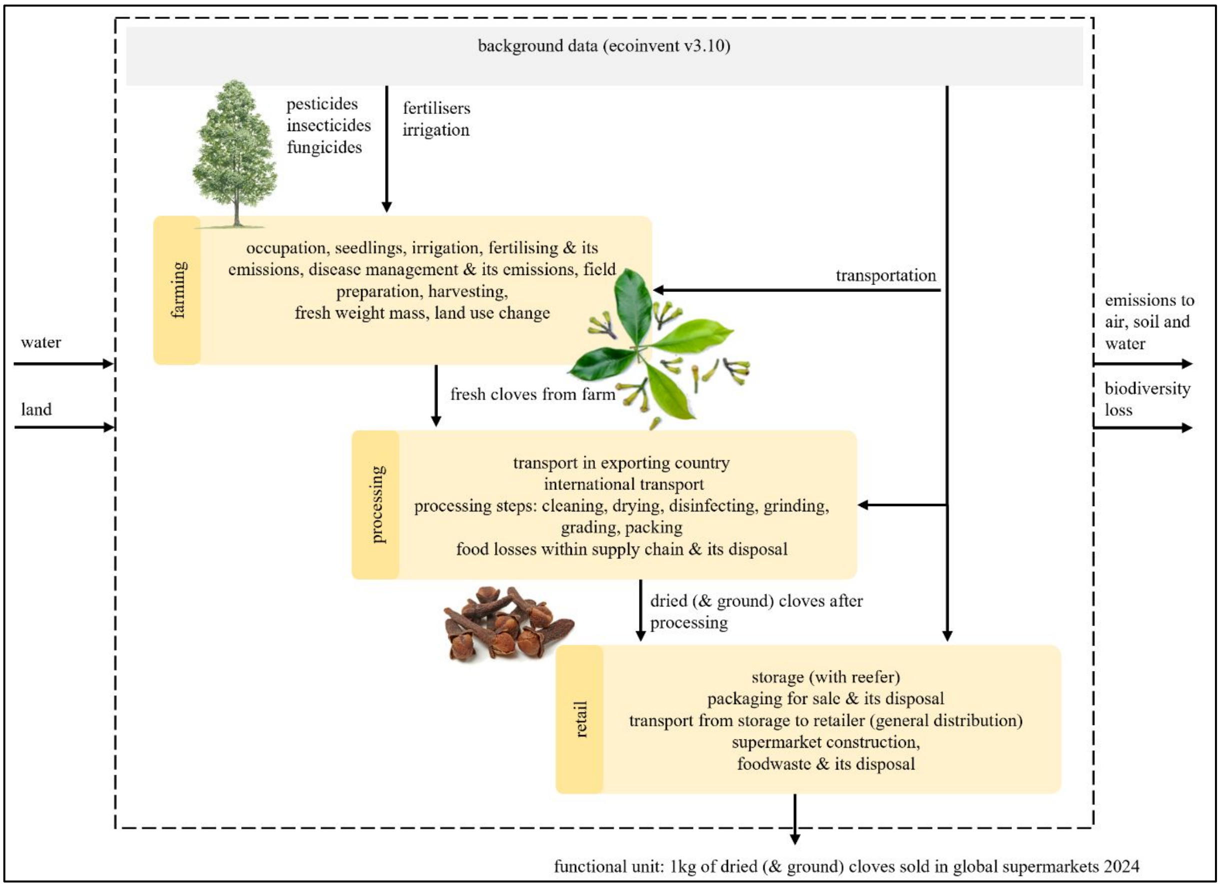

Figure 4: production system of 1 kg dried (& ground) cloves sold in supermarkets 2024.

4.3.2. Organic

Farming phase: Cloves are cultivated in wild clove tree forests, adhering to organic farming practices without using fertilisers, pesticides, irrigation, or other inputs. On one acre, 200 trees are grown, each yielding approximately 10 kg of dried cloves.

Processing phase: The processing steps, including harvesting, drying, grading, sorting, and packing, are all carried out manually. The transport from the field to the processing location is on a man's back. The only additional input is water, used for cleaning equipment during grading and sorting. During the drying process, cloves lose 25 % of their initial weight. The dried cloves are then packed into 25 kg bags (composed of 95 % polypropylene, 4 % calcium carbonate, and 1% paraffin) and shipped to Hamburg via Colombo. In Hamburg, the cloves are stored until they are requested by consumers (assumption of an average storage time of 1 month), either as whole cloves or as ground cloves. In case of pests, pressure disinfection is necessary which is the case in 5% of the amount. If no manifestation with pest, only steam sterilisation (autoklav) is applied with temperatures of 70-105°C. For the grounded cloves, its ground in a mill in Hamburg wich is cooled. The ground cloves is then packaged in glass jars. The jars are subsequently transported and sold in stores.

5. Cumin

5.1. General Information

Cumin (cuminium cyminium L.) belongs to the family Apiaceae and is cultivated for its seed. It thrives in moderate cool and dry climate where winters are not severe and humidity is low. It is used as a spice, either in mixed spices like curry or as a own spice. It is also used as essential oil for the perfume industry or cosmetics. (Meena et al., 2021)

Table 9: production amount and production countries of cumin.

Production amount	Anise, badian, coriander, cumin, caraway, fennel and juniper berries, raw: 2'803'164.35 t → 760'000t dry cumin
Producing countries	India (68.6%), Turkey (12.6%), Russia (3.3%), Iran (2.2%), Rest (13.3%), (FAO, 2022)

5.2. Inventory data

Table 10: inventory data of cumin.

**models**	**cumin conventional**
country	India
sources	(Aravind et al., 2020; Meena et al., 2021)
land use change	- 20y: 3.59 t CO2eq/ha*y
yield	- Fresh yield: 649 kg/ha<br>- Weight loss: 5%<br>- Dry yield: 616 kg/ha
occupation of land	- Annual 120 days
fertiliser	- 30 kg N/ha<br>- 20 kg P/ha.<br>- 10 t FYM/ha
pesticides/ fungicides/ insecticides	- Seed treatment: Thiram @ 2.5 g/kg seed, 42% active ingredient<br>- Herbicide: Fluchloralin @ 0.77 to 1.00 kg/ha , 400g/kg active ingredient<br>- Disease: Mancozeb (0.2%), Sulphur @ 20-25 kg/ha
seeds	- 12 kg/ha in Rajasthan (main producing area) (1 seed = 5mg → 2'400'000 seeds/ha) → 3697 seeds/kg
irrigation	- Drip irrigation<br>- 1026 m3/ha → 1.58 m3/kg spice
processing steps	- Harvesting → manually<br>- Threshing → machine<br>- Cleaning with vacuum gravity separator → 1.12 kWh/kg processing yield<br>- drying in partial shade → manually<br>- packaging in glass bottles (2128 g glass, 128 g plastic)
transport mode to importing countries	- transport by lorry within exporting country (1200 km)<br>- transport by sea freight from india to china (4160 km)<br>- general distribution in importing country (refrigerated)
important	although the model is only for cumin, it is used for the whole economic category of anise, badian, coriander, cumin, caraway, fennel and juniper berries, raw

5.3. Production system

Farming phase: The soil is prepared by ploughing at least twice and incorporating the FYM. In November, Cumin seeds are put in to the soil with a rate between 12 an 15kg/ha. To avoid any borne diseases in the early state the seeds are first treated with Trichoderma culture (10g/kg seed) or Thiram or carbendazim @ 2.5 g/kg seed. Seeding is either done manually or by line sowing with machinery. It is not recommended to grow cumin in intercropping system to better manage diseases. But crop rotation between cultivation is important to avoid diseases. Irrigation should be applied mainly in the first stadium, as older plants don't like too much water. For this, sprinkler or drip irrigation systems are used. In terms of weed management, hand weeding is commonly used in combination with Fluchloralin @ 0.77 to 1.00 kg/ha or Basalin @-2.5 kg/ha or Stamp F-34 @3.33 kg/ha should be applied as pre-emergence weedicide. Diseases like Blight can be controlled by spray application of Mancozeb (0.2%). For treatment of powdery mildew, Sulphur @ 20-25 kg/ha is applied. For fighting against pests, mainly neem oil is used.

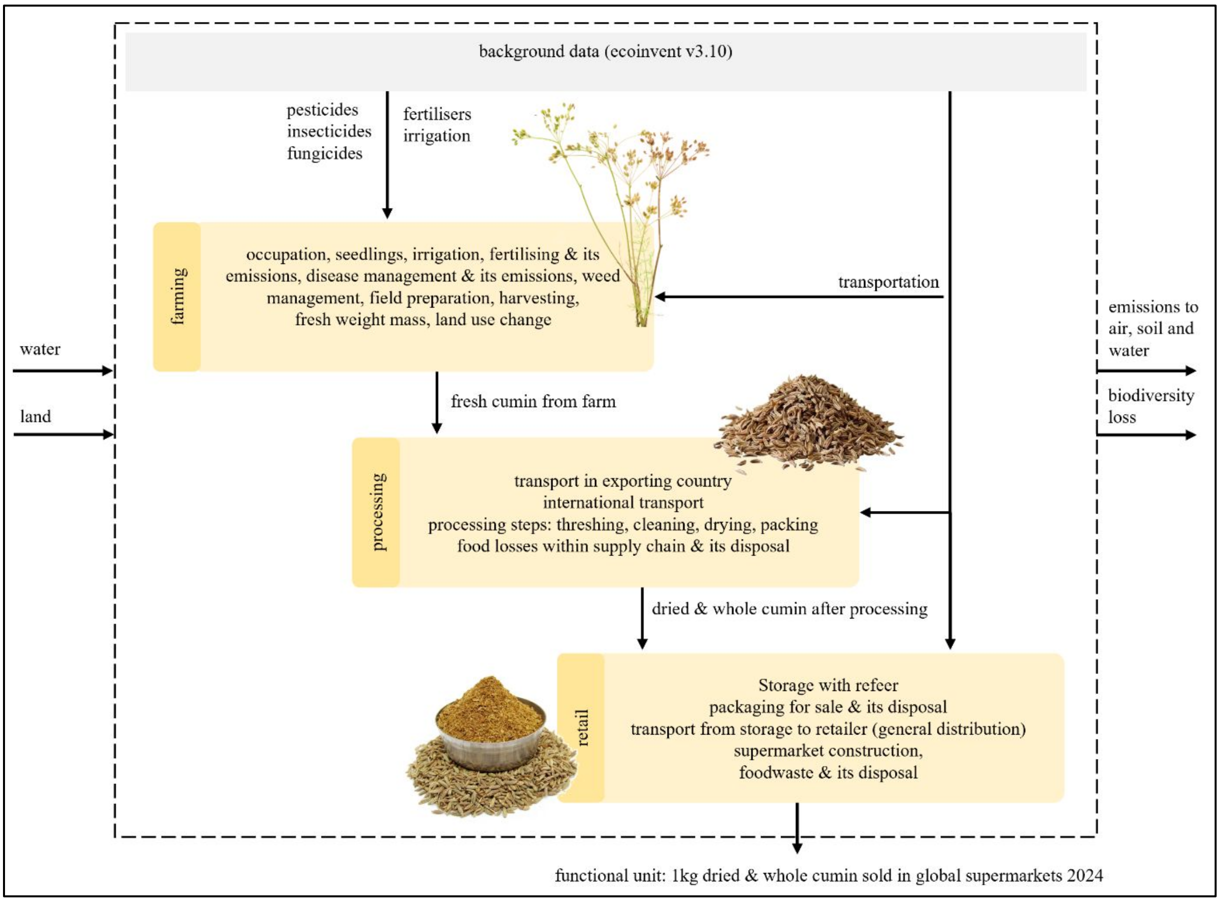

Figure 5: production system of 1 kg cumin seeds dried and whole sold in global supermarkets 2024.

Processing phase: After around 120 days, cumin can be harvested. Threshing is either done manually by trampling on a clean floor or by a thresher. The yield can be between 800 and 1000 kg/ha, but in reality it lays between 423.4, and 984.96 kg/ha due to bad techniques and management. For cleaning the seeds, a vacuum gravity separator is used. Then the seeds are dried in partial shade where the moisture level is kept at 9%. Packed in gunny bags, the seeds are further distributed.

6. Ginger

6.1. General Information

Ginger (*Zingiber officinale Roscoe*), belonging to the family Zingiberaceae, is a perennial plant typically cultivated as an annual plant in tropical and subtropical regions. The rhizomes, located near the soil surface, serve as the spice source (Kaufman, 2016). Ginger thrives at altitudes exceeding 500 metres above sea level in well drained sandy loam or clay loam soil enriched with humus and favours a pH range of 6 to 6.5, humidity levels of 70-90% and temperatures between 19-28°C. Partial sunlight, such as that found in coffee and coconut plantations, is ideal for cultivation (Saravanakumar, 2021). Possible diseases include Phyllosticta leaf spot disease and rhizome rot disease (Saravanakumar, 2021).

Table 11: production amount and production countries of ginger.

production amount	in the year 2022 it was 4’874’216 t (FAOstat, 2022) → 3’969’364 t dried and fresh sold
producing countries	India (46%), Nigeria (15%), China (13%), Nepal (6%), Indonesia (5%), Rest: 15% (FAO, 2022)

Since some processing steps and the transportation varies depending on whether the ginger is consumed fresh or dried and grounded, the distribution between these forms is approximated. Utilizing export and import data from the ITC trade map (ITC, 2022), the economic values reveal that approximately $123,000 is attributed to exported ground ginger, while fresh ginger commands around $777,000. Lacking precise volumetric data, an economically derived ratio is employed, indicating that 86% of fresh ginger is marketed without processing, with the remaining 14% being subjected to drying and grounding.

6.2. Inventory data

Some farmers in India are cultivating ginger in intercropping systems with maize, French bean, and pumpkin to achieve a better benefit cost ratio and therefore improve their economic conditions (Rymbai et al., 2021). However, ginger is mainly grown in monoculture systems (Saravanakumar, 2021) and therefore modelled as a monoculture cropping system in the conventional model.

Two different case studies are modelled in this study:

1) Organic production in Madagascar with primary data from the organic spice provider, grounded ginger
2) Conventional production in India with literature values, fresh ginger

Table 12: inventory data of ginger.

**models**	**Organic model (1)**	**Conventional model (2)**
country	Madagascar	India
sources	organic spice provider 2024	(saravanakumar, 2021) & (soni et al., 2022) & (bhutia et al., 2022).
land use transformation	20y: 0kg or -0.085759584t co_2eq/ha<br>50y: 12.3t co_2eq/ha	20y: 2.67 tco_2eq/ha

yield	- 12'500 kg fresh ginger/ha<br>- 85% weight loss<br>- 1'785 kg dried ginger/ha	- 10'700 kg fresh ginger/ha
occupation of land	annual crop, 9 months occupation	annual crop, 9 months occupation
fertiliser	organic manure (3 kg/are → 300 kg/ha)	mineral NPK fertiliser<br>80n:100p:80k kg npk/ha
pesticides/ fungicides/ insecticides	none	fungicide (mancozeb & carbendazim)
rhizome seedlings	1500 kg/ha	55'500 rhizomes/ha → 227 kg/ha
irrigation	none	3117.45 m3/ha, drip irrigation
processing steps	- Harvesting → manually<br>- cleaning, slicing, drying, packing → manually, 2l water<br>- disinfection & grinding in hamburg (1.12 mj/kg spice)<br>- packaging transport: in plastic bags<br>- packaging sale: glass bottles (2.702 kg/kg ground ginger)	- harvesting: by tractor<br>- cleaning, slicing, packing → manual<br>- drying → solar drying<br>- packaging transport: cardboard boxes<br>- packaging sale: none
transport mode to importing countries	- transport by lorry within madagascar (1200 km)<br>- transport by ship to hamburg (17'300 km)<br>- general distribution in import country (not refrigerated)	- transport by train, cooled (416 km)<br>- transport by lorry, cooled (1200 km)<br>- transport by ship (12151 km)<br>- general distribution in import country (refrigerated)<br>- storage refrigerated
important	economic allocation	

6.3. Production sytem

6.3.1.Conventional

Farming phase: before the agricultural cultivation starts, the field is prepared. After the first rain showers, the field is ploughed 2-3 times followed by soil solarisation in case of a history with rhizome rot disease. Soil solarisation mitigates the rhizome rot disease in ginger cultivation. By covering the soil with a polyethylene sheet for about six weeks before planting, pathogens will die because of the accumulated heat underneath. It is most effective during extended periods of dry and sunny weather (Saravanakumar, 2021). Usually, the foil is based on polyethylene plastic and between 0.02 and 0.05 mm thik and covers the filed completely (Cherlinka, 2023). The densitiy of polyethylene film is 0.94g/cm3 (Kunststoff Schweiz, 2024). Based on these informations the total weight used to cover 1ha is 188 kg polyethylene film (0.02mm *1ha = 200'000cm3 → *0.94g/cm3 = 188'000g →188 kg). As the film can be reused if used colorless (Cherlinka, 2023), the assumption of a 10 year usage of this film is made. Additionally, the planting material is cultivated or bought. For planting, seed rhizomes or ginger seedlings can be used (Saravanakumar, 2021). After washing the seed rhizomes, they are treated with a fungicide to prevent the rhizome rot disease and put in a growing soil mix (Saravanakumar, 2021). The rhizomes are for example dipped in carbendazim 12% and mancozeb 63% WP @ 2g/l for 30 minutes

and then dried in the shade for 3 to 4 hours (Sathya, 2022). It is assumed, that the mix can be used for 20 kg rhizomes. Since carbendazim is a Benzimidazole-compound (PubChem, 2024) and not included in the Ecoinvent database, the chemical compound benzimidazole is used.

The agricultural cultivation starts with transplanting the ginger seedlings after transporting them to the fields. By creating holes, the seedlings are planted with 30 cm space between plants and 60-75 cm space between rows. For 1 ha this corresponds to 55'500 seedlings. For this, single bud rhizomes with an average weight of 5g can be used. Hence, 277.5kg rhizomes are needed for 1 ha. In case of irrigated cultivation, drip irrigation is recommended to increase water efficiency and prevent rhizome rot as well as wilt pathogen. Supplying enough water is crucial, especially right after transplanting and during development stages of the rhizomes. Rainfed cultivation is best when the rainfall is even distributed. However, irrigated cultivations show more productivity. As Ginger requires high amount of nutrients, fertilising nitrogen during the active vegetative growth (day 60-90) and fertilising potassium during rhizome development (days 130-190) is crucial (Saravanakumar, 2021). A general recommendation of an NPK fertiliser is 80N:100P:80K kg NPK/ha cultivation (Soni et al., 2022). Of course, organic fertiliser can be used and replace a part of the fertiliser. The efficiency of the fertiliser can be increased by hilling up the ginger cultivation. Generally, herbicides are avoided and only applied if needed (Saravanakumar, 2021). Mulching after 4 to 6 weeks is the primary used weed management which uses banana or mango leaves. Disease management is crucial to avoid harvest losses. The rhizome rot disease is caused by water stagnation due to heavy rainfall and shows symptoms such as yellow or wilting leaves. Responsible for this disease are pathogenic fungi and bacteria in combination with a nematode complex. To mitigate this disease, ginger cultivation is rotated every year and grown in intercropping systems with legumes like corn or peanut. Biopesticides (VAM, Bacillus or Trichoderma) can be applied preventive. Further, caterpillar can infest the ginger. By using neem based or bacillus thuringiensis based organic insecticides the caterpillars can be controlled (Saravanakumar, 2021). These kind of pesticides are not included in the model since it is a bacteria. Further diseases are thripse, and spider mites (Discover Agriculture, 2023). Depending on the purpose of ginger, harvesting time differs. For producing ginger in powder form, it is harvested after 210-240 days when the leaves are turning yellow. For the use as a vegetable, harvesting takes place after 180 days. Harvesting in smaller farms is done manually, whereas larger farms harvest with a harvester led by a tractor. (Saravanakumar, 2021) For simplicity, the general average of nine months cultivation time is used. A comparative study on India's national productivity of ginger cultivation from 2016 to 2019 revelaed an average yield of 10.72 t/ha, ranging from 4.89 t/ha up to 22.08 t/ha in regions like Gujarat (Bhutia et al., 2022). Reasons for higher yields are different varieties and a more effieicient, scientific management system (Bhutia et al., 2022).

Processing phase: The local processing right after harvesting starts by trimming the rhizomes from the roots and foliage, washing them, and drying for an hour. Afterwards, the ginger is transported by lorry at 10°C to larger processing locations. The ginger is cleaned again and graded by the help of grading tables and screens. To reach a thicker skin, the ginger is cured by drying it at 22-26°C and 70-75%

relative humidity for 14 days. Preferably by solar drying which has the advantage of eliminating microbial contamination in comparison to sun drying (Saravanakumar, 2021). Ginger is packaged in aerated bags, such cardboard boxes. It is important that fresh ginger is stored at a fumigated and dry room at a temperature of 12°C and a humidity level of 70-75% (Saravanakumar, 2021).

In terms of transportation, the distance between ginger-producing countries, particularly India and China (ITC, 2022), and major importing nations like the Netherlands and Germany (ITC, 2022), has been studied. Notteboom & Rodrigue (2011) illustrate the primary route from ginger-producing regions like India to the Netherlands via maritime transport, typically passing through the Suez Canal and Gibraltar, ultimately reaching Rotterdam. Using the Cargo Calculator (SeaRates, 2024), the estimated distance via sea transport is 12,151 km. Considering the transportation within India, ginger is typically transported from various cultivation regions to Mumbai, covering an average distance of approximately 416 km by train and the general transporation within the exporting country of 1200 km by lorry. In the end, ginger is sold in supermarkets as fresh or grounded ginger and takes up some space in the supermarket which is included in the assessment.

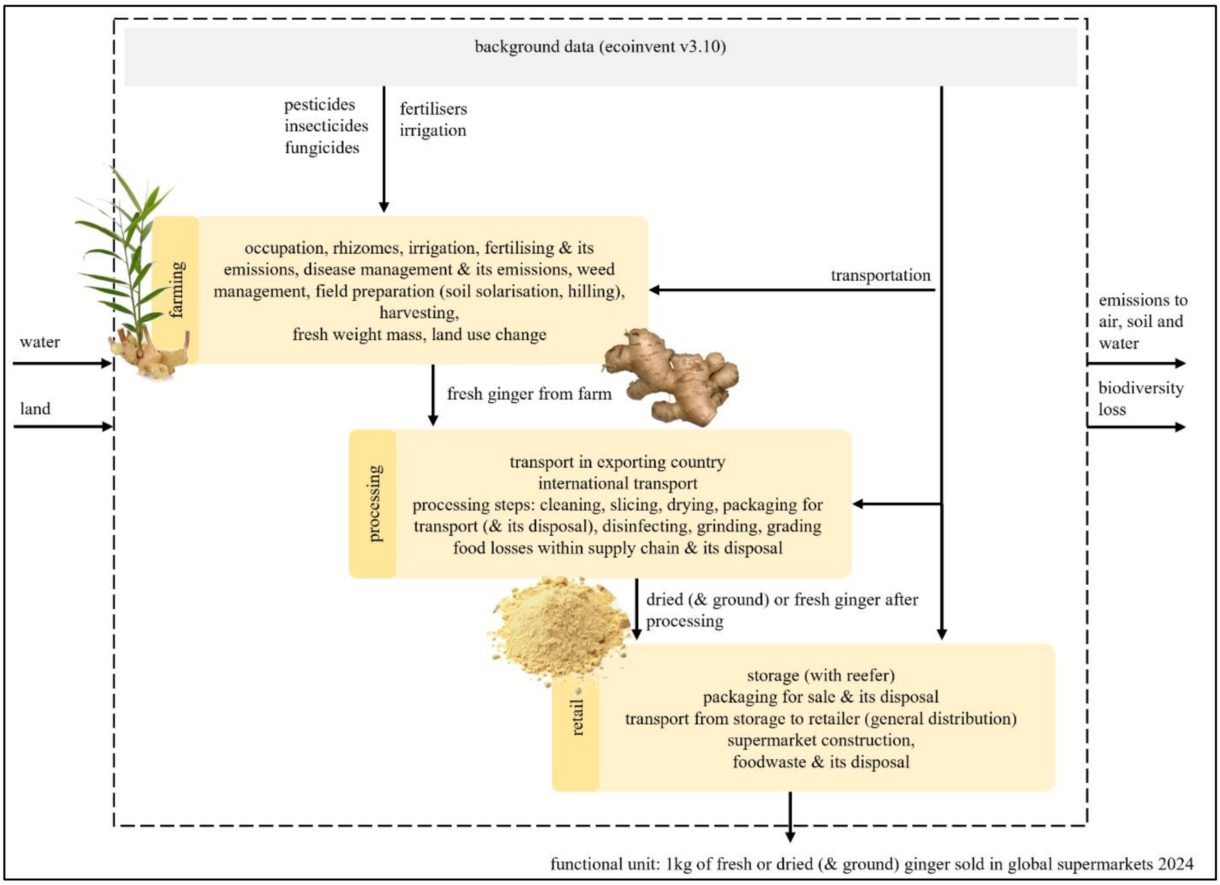

Figure 6: production system of 1 kg fresh or dried (& ground) ginger sold in global supermarkets 2024.

6.3.2. Organic

Farming phase: ginger is cultivated in a intercropping system with crops like cassava (7'000kg/ha), pineapple (50'000kg/ha), litchi (5'000kg/ha), or banana (30'000kg/ha). Since different crops are cultivated, an economic allocation is performed. The exact market price of these products will remain

confidential, therefore only the price relation of 1:15 (pineapple:ginger) is documented. These allocation factors are included in the farming stage, meaning that 79 % of the emissions are allocated to ginger, whereas the remaining 21 % are allocated to pineapple. This extensive cultivation method avoids pesticides, mineral fertilizers, and irrigation, relying entirely on manual labor. The only input during the farming stage is organic manure from cattle (300 kg/ha). Harvesting is performed manually, and the ginger is transported on a man's back to the nearest processing facility. The plant residues remaining on the field are assumed to be 450 g per kg ginger rhizome (Robertson et al., 2023).

Processing phase: it the facility, the ginger is washed using tap or river water where no pumps are needed. The tropical climate ensures an ample water supply, preventing issues with water scarcity. After washing, the ginger is sliced and sun-dried until it becomes completely dry chips. These chips are then packed into 25 kg bags composed of 95% polypropylene, 4% calcium carbonate, and 1% paraffin. (Calculation: densitiy of ginger: 88g/236.6cm3 → volume for 25 kg is 236.6/88*2500 = 6727.3cm3 =6.73l content of ginger chips. Add some air, since its not grinded but in chips form = 8l = 8000cm3. Plastic bag this size is around 15g). The packed ginger chips are transported by lorry to Tamatave and then shipped to Hamburg via Colombo. In Hamburg, the ginger chips are stored until they are requested by consumers (assumption of an average storage time of 1 month), either in chips form or as grinded ginger. In case of pests, pressure disinfection is necessary which is the case in 5% of the amount. If no manifestation with pest, only steam sterilisation (autoklav) is applied with temperatures of 70-105°C. For the grounded ginger, its ground in a mill in Hamburg wich is cooled.

Retail phase: the ground ginger is then packaged in glass jars, with each jar weighing about 85 grams for 40 grams of ginger powder. The jars are subsequently transported and sold in stores.

7. Nutmeg & Mace

7.1. General Information

Nutmeg and mace both originate from the evergreen nutmeg tree (*Myristica fragrans*), which can reach heights of up to 18 meters. The seed's kernel is harvested as nutmeg, while the leathery, net-like aril surrounding the seed is processed into mace. Although they share similar flavors, mace is considered more delicate. (Spence, 2024)

Nutmeg trees thrive in temperatures between 20–30°C, with 1500–2000 mm of rainfall and loamy sandy soil. Approximately half of the nutmeg trees are male and do not bear fruit, but their sex is only identifiable after 6 to 8 years when they first flower. To optimize fruit production, seedlings are planted closely, and once the males are identified, most are removed, leaving a typical ratio of one male for every ten female trees for pollination. (Azam-Ali, 2007)

Both products are mainly used as a spice but also for essential oil production and medicinal purposes (Azam-Ali, 2007). Nutmeg and mace are typically sold whole, as their ground form loses aroma quickly.

Table 13: production amount and production countries of nutmeg and mace.

Production amount	Cardamom, nutmeg and mace yields in 138'887 kg in the year 2022 (FAO, 2022) → 21.8% nutmeg = **30'277 t, 22'910t dried**; 9.1% mace = **12'639 t, 5'100 t dried**
Producing countries	India (29.5%), Indonesia (29.2%), Guatemala (26.2%), Nepal (6.3%), Rest: 8.8% (FAO, 2022)

7.2. Inventory data

The system, modelled for this study is an Indian conventional cultivation system where only whole nutmeg and mace are considered.

Table 14: inventory data of nutmeg and mace.

**models**	**conventional model nutmeg**	**conventional model mace**
country	India	India
sources	(Azam-Ali, 2007; Spence, 2024; TNAU Agritech, 2013)	(Azam-Ali, 2007; Spence, 2024; TNAU Agritech, 2013)
land use change	20y: 0.1 t co2eq/ha	20y: 0.1 t co2eq/ha
yield	- nutmeg: 2'100 kg fresh nutmeg, 25% weight loss<br>- 1'575 kg dried nutmeg/ha	- mace: 393.7 kg fresh mace,<br>- 60% weight loss<br>- 157.5 kg dried mace/ha
occupation of land	perennial	
transformation	To account for the agroforestry type of system, the transformation is considered to forest intensive.	
fertiliser	- 50 kg FYM/tree → 11'250 kg FYM/ha<br>- 300 g n/tree → 67.5 kg n/ha<br>- 300 g p/tree → 67.5 kg p/ha<br>- 960 g k/tree → 216 kg k/ha	
pesticides/ fungicides/ insecticides	- 20 kg bordeaux mixture (1% → 20 kg in 1000l)	
corms (seedlings)	12kg/ha	
irrigation	2844.82 m3/ha, mixture irrigation	
processing steps	- harvesting (manual)	- harvesting (manual)

	- separating aril from nut (manual)<br>- sun drying<br>- packing in jute bags for transport<br>- packaging glass (2.632 kg/kg nutmeg)	- separating aril from nut (manual)<br>- sun drying<br>- packing in jute bags for transport<br>- packaging: plastic bag
transport mode to importing countries	- transport by lorry within country<br>- transport by sea freight from india to singapore (2900 km)<br>- detailed distribution within importing country	- transport by lorry within country<br>- transport by sea freight from india to singapore (2900 km)<br>- detailed distribution within importing country
important	economic allocation between mace and nutmeg	

7.3. Production sytem

Farming phase: nutmeg (*Myristica fragrans*) is cultivated as a perennial crop with propagation most commonly achieved through seeds obtained from fruits that have naturally split open. The seed rate varies between 12 and 16 kg/ha (Aravind et al., 2020). For optimal growth, the trees require regular fertilization, with recommended applications per tree per year including 50 kg of farmyard manure (FYM), 300 g of nitrogen (N), 300 g of phosphorus (P), and 960 g of potassium (K) (TNAU Agritech, 2013). To mitigate water stress during the summer months, irrigation is advised and commonly practiced (Sumbula & Mathew, 2015). Nutmeg cultivation is susceptible to various fungal diseases, which can significantly reduce yields. One of the most detrimental is leaf fall caused by *Phytophthora* sp., which necessitates treatment with fungicides. In a study by Sumbula and Mathew (2015), various treatments were tested both in vitro and in vivo, with the most effective method being the application of a 1% Bordeaux mixture. Copper-based fungicides, including Bordeaux mixture, have been proven effective against leaf fall. The application of 2 kg of Bordeaux mixture in 100 liters of water, assuming a total application of 1,000 liters per hectare, equates to the use of 20 kg of Bordeaux mixture per hectare (Sumbula & Mathew, 2015). Harvesting of nutmeg occurs when the fruit naturally splits open and falls to the ground, at which point it is collected for processing. The yield varies depending on the age of the tree. Nutmeg trees begin flowering and bearing fruit after six years, with maximum yield reached at around 40 years of age, during which yield steadily increases (Azam-Ali, 2007). A mature nutmeg tree can produce between 1,000 and 10,000 fruits, yielding on average 5-7 kg of dried nuts and 0.5-0.7 kg of dried mace per tree (TNAU Agritech, 2013). ver a 100-year lifespan, assuming an average of 2,000 fruits per tree and a planting density of 250 trees per hectare (225 of which are female), the estimated yield is 1,575 kg of dried nuts per hectare and 157.5 kg of dried mace per hectare (TNAU Agritech, 2013). Since the inputs on the farming level are the same for the two spices, an economic allocation is carried out between nutmeg and mace. The allocation is carried out based on market prices from July 2024. The price for nutmeg lays between 3.97 and 6.19 US$/kg and of mace between 1.48 and 3.45

US$. The average price of both spices is taken for further calculations (5.08 US$/kg nutmeg, 2.47 US$/kg mace).

- value added:
    - nutmeg: 1‘575 kg /ha, 5.08 US$/kg => 1575kg/ha *5.08 $/kg = 8001 $/ha
    - mace: 157.5 kg/ha, 2.47 US$/kg => 157.5 kg/ha * 2.47 US$/kg = 389 $/ha
- allocation factor:
    - 8001/ (8001+389) $ = 0.954
    - 389 / (8001+389) $ = 0.046

These allocation factors are included in the farming stage, meaning that 95.4% of the emissions are allocated to nutmeg, whereas the remaining 4.6% are allocated to mace.

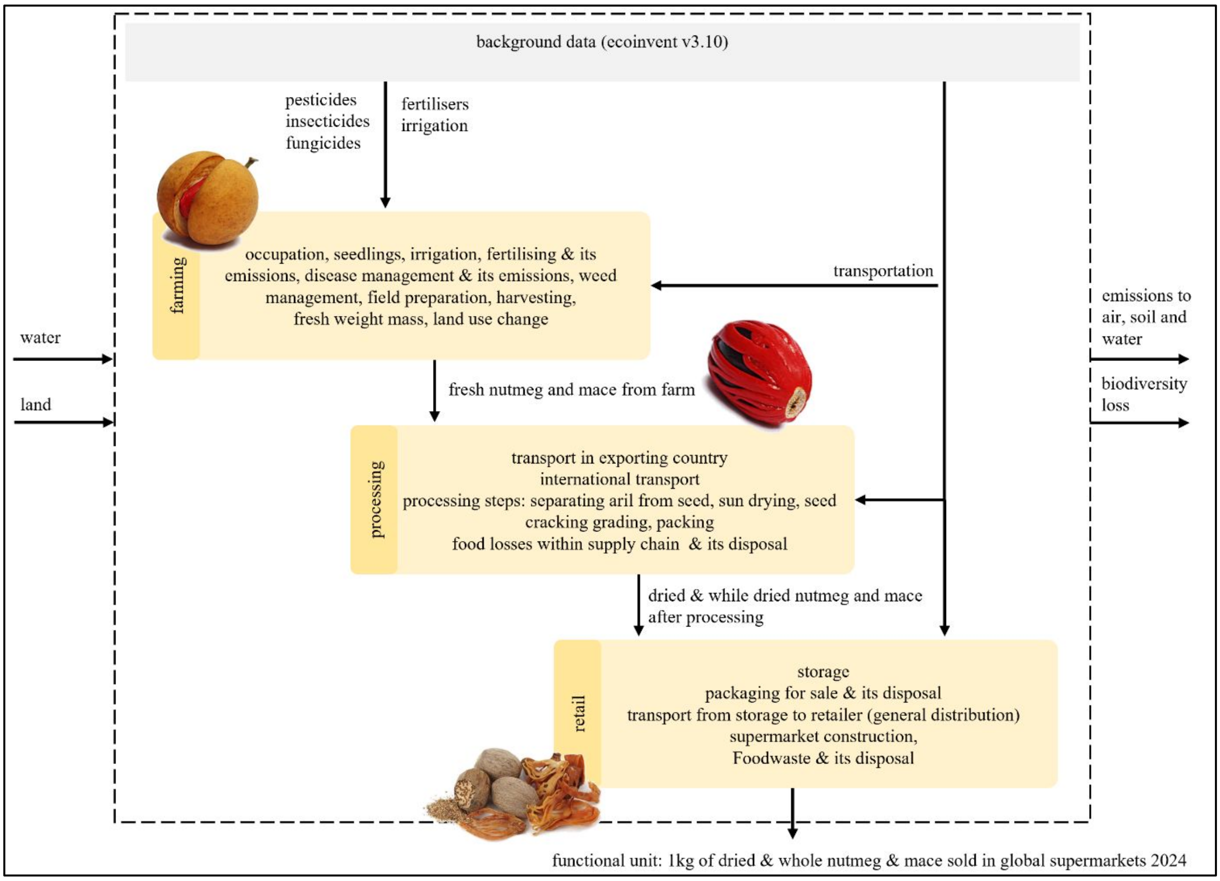

Figure 7: production system of 1 kg dried & while nutmeg & mace sold in global supermarkets 2024.

Processing phase: the aril (mace) is manually separated from the seed using machetes (Kembauw et al., 2023). In case of the mace, it is first flattened and then sun dried on mats for about four hours to two days (Azam-Ali, 2007; Kembauw et al., 2023). Afterwards, the dried mace needs to be stored for four months before using it to be cured. Only then, the mace can be graded and packed. The nutmeg seeds are sun-dried for one week, during which time they are turned daily to prevent fermentation (Kembauw et al., 2023). Nutmeg are either sold with the shell or without. By tapping the end of a nut with a wooden instrument, the seeds can be cracked. The resulting kernel are then sorted based on quality and size and ready for the export. For transportation, nutmegs are typically packed into gunny bags or hessian sacks (Azam-Ali, 2007).

8. Pepper

8.1. General Information

Pepper (*Piper nigrum*) is the world's most traded spice and cultivated for its fruit. It belongs to the family Piperaceae (IFEAT, 2022) and is a climbing plant, reaching heights until 15 m, native in tropical warm Asian regions (Duwe, 2022). Despite its role in the kitchen, pepper is also used for industrial and pharmaceutical purposes as well as the perfumery (IFEAT, 2022).

Depending on the form of the pepper the taste differs from the burning hot flavour of black pepper, to very mild in the case of green pepper. It is consumed as whole berries, crushed or ground (Duwe, 2022). Pepper is available in four forms, including black, white, red, and green, each differing in processing stages and treatments. Black pepper is harvested as unripe green berries, fermented and dried, resulting in a black appearance. For producing green pepper, unripe berries are preserved in vinegar or brine and then dried, retaining their green colour. White pepper, however, is obtained from fully ripe red berries that are submerged in water to soften the skin and to promote fermentation. Afterwards, the outer skin is removed, which leaves only the inner seed, which is then dried. Red pepper also consists of fully ripe berries, which are dried without fermentation, thus maintaining their red colour (K Agriculture, 2022b). Ideal growing conditions include a hot and humid climate between 24°C and 30°C. Pepper is cultivated either in mixed cropping systems, where the vines are supported by attaching to other plants, or in monoculture systems, where the plants require artificial support structures (Vogel, 2023). Conventional monoculture cultivation is primarily practised in Brazil, Indonesia, and Vietnam, often involving the clearing of rainforests for cultivation. In mixed cropping systems, pepper is grown alongside crops such as mango, coconut, and nutmeg in Sri Lanka, which serve as supporting plants. In India, pepper is commonly intercropped with tea or coffee plantations (Vogel, 2023).

Table 15: production amount and production countries of pepper.

production amount	812,674 metric tons in 2022 (FAOstat, 2022) in 43 countries worldwide → 396'416 t dried
producing countries	Vietnam (33.5%) → 272'245, Brazil (15.8%) → 128'402, Indonesia (10.1%), Burkina Faso (9.5%), India (7.9%), Rest: 23.2% (FAO, 2022)

8.2. Inventory data

Two different models are listed, but only the conventional one is modelled:

1) Conventional monoculture in Vietnam/Indonesia/Brazil
2) Organic intercropping system in India/Sri Lanka

Table 16: inventory data of pepper.

models	Conventional monoculture	Organic intercropping (not modelled)
country	Vietnam/ Brazil	India/ Sri Lanka
sources	(Carvalho et al., 2023)<br>(Oliveira et al., 2021)	(Devasahayam et al., 2015)<br>(IFEAT, 2022)

	(TNAU Agritech, 2022)	
Land use change	20y: agricultural area<br>50y: rain forest	20y: agricultural area<br>50y: rain forest
Yield	- 2500 kg fresh pepper/ha<br>- Weight loss: 52.7% (Roslan & M. Yudin, 2020)<br>- 1182.5 kg dried pepper/ha	- In India and Sri Lanka: 500 – 1000 kg/ha (IFEAT, 2022)<br>- Weight loss: 52.7% (Roslan & M. Yudin, 2020)<br>- 473 kg dried pepper/ha
Occupation of land	Perennial (IFEAT, 2022)	Perennial (IFEAT, 2022)
Fertiliser	- 15 kg manure /vine<br>- 100 g N, 50 g P, 150 g K/vine<br>- Slaked lime 500g/vine every second year<br>- 150g rock phosphate/vine<br>(TNAU Agritech, 2022)	- 5-10kg FYM per vine (Devasahayam et al., 2015).
pesticides/ fungicides/ insecticides	- Bordeaux mixture<br>- Dimethoate<br>- Quinalphos<br>(TNAU Agritech, 2022)	- Neemgold (0.6%) spray (Devasahayam et al., 2015).<br>- 1% Bordeaux mixture (Devasahayam et al., 2015).
Corms (seedlings)	1’250 vines per ha	Cuttings: 2,500 pepper vines/ha (IFEAT, 2022).<br>- Indian mixed crop system: 540-560 vines/ha (Vogel, 2023)
Irrigation	444.02 m3/ha (calcualted), sprinkler irrigation	- On average 40l/vine every two weeks for 2 months
Processing steps	- Threshing mechanically<br>- Drying artificial<br>- Classifying & sieving<br>- Packaging: glass bottles (1660 g glass, 50 g plastic)	- Threshing by hand<br>- Blanching (black), soaking (white) or preserving (green)<br>- Sun drying<br>- Classifying & sieving<br>- Packaging:<br>-
transport mode to importing countries	- transport by lorry within exporting country (1000 km)<br>- Transport by sea freight from Vietnam to Los Angeles (USA) (13’300 km)<br>- General distribution within importing country (not refrigerated)	- By lorry within exporting country<br>- Transport by sea freight to Hamburg<br>- General distribution within importing country (not refrigerated)
Important		- Intercropping: areca nut, coconut, nutmeg, coco (IFEAT, 2022)

8.3. Production sytem

Farming phase: The seedlings used for pepper plants can be propagated by several methods. The one used in this model is propagating through cuttings. Runner shoots are separated and put in polyethylene bags which are filled with a potting mix consisting of soil, sand and farm yard manure (2:1:1). Frequent irrigation and shade are important for them to grow. The growing takes about 3 months (Devasahayam et al., 2015).

The yield in Vietnam lays between 2500-3000 kg/ha (IFEAT, 2022) and in Brazil around 2’250 kg/ha (Oliveira et al., 2021). In India and Sri Lanka however, the yield is estimated to be lower with 500 to 1000 kg/ha and up to 1’100 kg/ha in Indonesia (IFEAT, 2022).

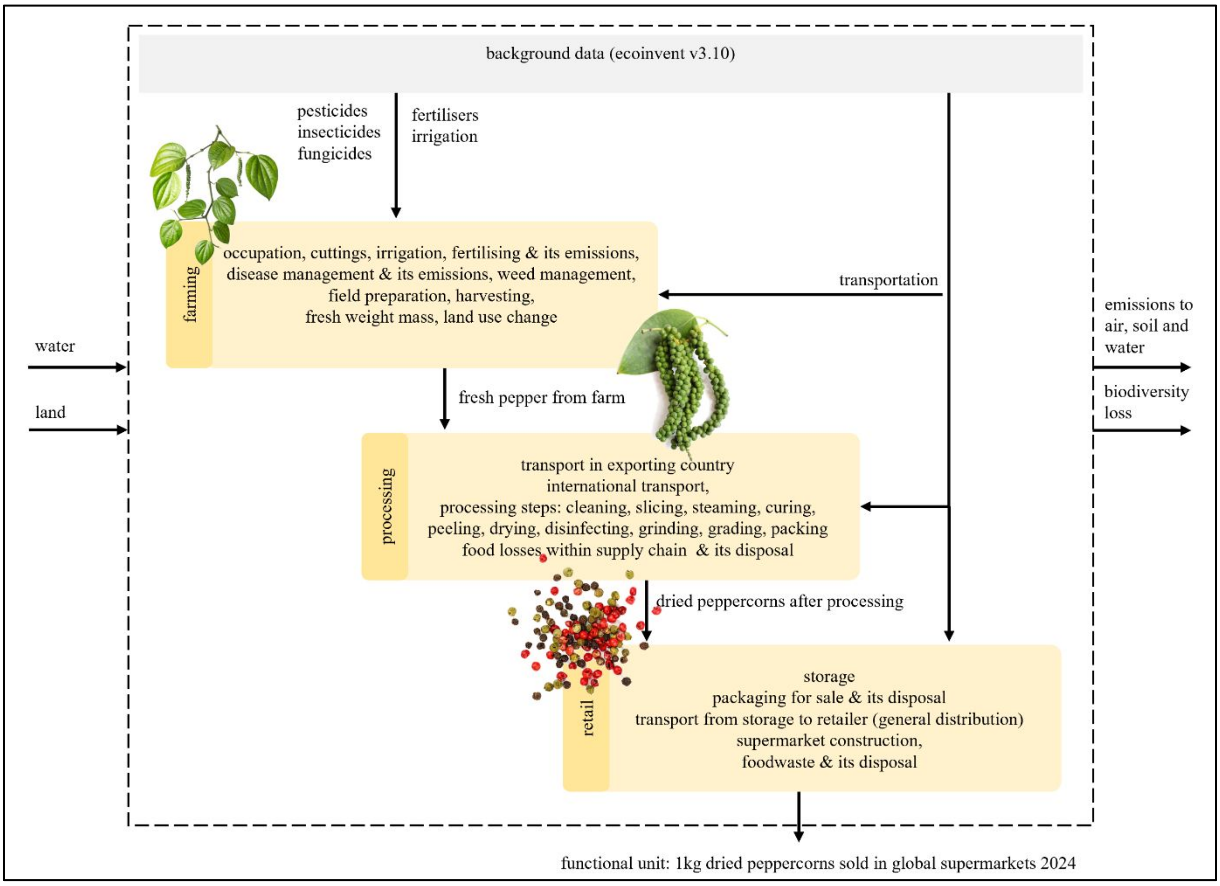

Figure 8: production system of 1 kg dried peppercorns sold in global supermarkets 2024.

For cultivation in monoculture, concrete pillars with a diameter of 15 cm are used, 3.5-4 m in height. Volume in m3 per pillar is 0.15m* pi *4m in height = 1.89m3. By maintaining a planting distance of 2.5 m to 3 m, 1’111 to 1’600 plants/ha are cultivated (K Agriculture, 2022b). Indian monoculture systems usually have 1100 vines/ha, whereas a comparative report of Asian countries plants on average 2,500 pepper vines/ha (IFEAT, 2022) and India uses 1’600 plants/ha (K Agriculture, 2022b). For this model, 1’250 vines/ha are used. In the case of using plants as pillars, shade is sufficiently provided. But in the case of concrete, shade trees are necessary. For this purpose, every third row needs trees instead of dead pillars. Trees like durian, cassia or other shade trees can be used (K Agriculture, 2022b). Therefore, 417 of the pillars are modelled as plants and 833 as concrete pillars. The life expectancy of concrete pillars is assumed to be 30 years as it does not have to fulfil any load-bearing function (Noyce & Crevello, 2016). When planting two rooted cuttings next to each pillar, 5kg of farm yard manure and 150 g rock phosphate are added per pillar (Devasahayam et al., 2015). When planted, 10 kg of cattle manure per vine and chemical NPK fertiliser with 50: 50: 150 g per vine per year (Devasahayam et al., 2015). Irrigation is not always necessary but general irrigation increases yield, as pepper is sensitive to water deficit (Carvalho et al., 2023). In traditional mixed cropping systems irrigation is not necessary (Mariyam, 2023). Irrigating every second week about 40 l per vine showed an increased yield of 90-

100% in India (Devasahayam et al., 2015). Drip irrigation or sprinkler irrigation systems are mainly used for irrigation (Mariyam, 2023). Disease management is complex and depends on what pests and disease are appearing and of course on the region. To prevent foot rot disease, spike shedding, and pollu disease, a bordeaux mixture is applied (1%) on leaves where 400 g active substance per 100 l are used and 5l per vine are applied (Devasahayam et al., 2015). To control virus diseases like stunt disease, the insecticide dimethoate (0.05%) is applied. Quinalphos (0.05%) is effective against pests and sprayed on the leaves. The harvesting is done manually.

Processing phase: After harvesting, the berries are threshed manually or mechanically to separate berries from the stem (IFEAT, 2022). In case of a mechanical threshing, between 50 kg and 2500 kg per hour can be threshed (Devasahayam et al., 2015). A treshing machine is around 140 kg (UGOOD, n.d.). Then, the processing differs depending on the wished peppercorn. In this study, only the black peppercorn, as it has the highest demand, is modelled.

- Black pepper: After threshing, the peppercorns are dried either with artificial dryer (max. 55°C) or by sun drying for 3 days until the targeted moisture content is reached (Oliveira et al., 2021). Sometimes a blanching step is performed before drying where the berries are dipped in boiling water for one minute to provide a uniform coloured black peppercorn and shorten the drying afterwards (Devasahayam et al., 2015). Peppercorn has a moisture content of 65% to 70% and should be reduced to under 10% (Devasahayam et al., 2015).
- White pepper: After harvesting, the peppercorns are soaked in water for 15 to 30 days (Oliveira et al., 2021) or less like 7-8 days (Devasahayam et al., 2015) to peel off the shell. Water is changed three times during this process. Drying under the sun for the following three days finishes the processing of the white pepper. After air drying and sieving, it is ready for exporting. (Oliveira et al., 2021)
- Green pepper: After harvesting, the fruits are preserved in brine or vinegar

In the end, peppercorns are classified depending on size and quality by using sieves and dividing them into different categories (Devasahayam et al., 2015; Oliveira et al., 2021).

Retail phase: the pepper is now sold as peppercorn or as ground powder. The peppercorns are collected within the producing countries by lorry, packed in big bags and shipped via sea freight to Hamburg (S. Stamer, personal communication, 29 November 2022).

9. Saffron

9.1. General Information

Saffron (*Crocus sativus L.*) stems from the family *Iridaceae* and from the genus *Crocu*s. Only the *Crocus sativus L.* is cultivated as a spice for its stigma (Leone et al., 2018). Saffron has a typical aroma, caused by the safranal, a strong colour caused by crocin and a bitter flavour because of the picrocrocin (Cagliani et al., 2015). Saffron is able to grow in areas with droughts during the summer and its habitat ranges from areas at sea level up to 2000 m above sea level, preferably growing in areas from 600 to 1700 m above sea level (Salwee & Nehvi, 2013). The major use of saffron are as a spice, as medicine and as colorant. Since saffron contains crocin which shows antitumor and anticancer properties, it is used in plant-based medicine (Kothari et al., 2021). It is grown as a perennial crop mainly in Iran (> 90%) (Cagliani et al., 2015; FAO, 2018), but also in Spain, Italy, India, Afghanistan, Azerbaijan, Turkey, Egypt, France, UAE, Israel, China, Iraq, Greece, Japan, Australia, and Switzerland (Cardone et al., 2020).

Table 17: production amount and production countries of saffron.

production amount	418 - 450 t saffron (Statistia, 2019), (Cardone et al., 2020), 90 t dried saffron
producing countries	- Iran (90%) (Cagliani et al., 2015; FAO, 2018),<br>- Rest: 10%

9.2. Inventory data

Since saffron is mainly cultivated in Iran (Cagliani et al., 2015; FAO, 2018), only one conventional model with data displayed in table X is used.

Table 18: inventory data of saffron.

**models**	**conventional model - dried saffron**
country	Iran
sources	(andabjadid et al., 2015; asadi et al., 2016; cagliani et al., 2015; cardone et al., 2020; dar et al., 2017; ghorbani & koocheki, 2017; husaini et al., 2010, 2010; kafi et al., 2018; koocheki et al., 2019; kumar et al., 2008; leone et al., 2018; mohammad, 2012; mollafilabi et al., 2021; nehvi et al., 2014; salwee & nehvi, 2013; sepaskhah & kamgar, 2009) and more
land use transformation	20y: 1.83 kg/kg fresh saffron
yield	17.5 kg fresh saffron/ha (only stigmas)<br>weight loss 80%<br>3.5 kg dried saffron /ha
occupation of land	annual crop
fertiliser	- 10t manure/ha before planting<br>- 20t cow manure/ha<br>- 50 kg urea/ha
pesticides/ fungicides/ insecticides	herbicide: 1kg (900 g active substance) atrazine /ha
corms (seedlings)	2.5-3 t corms or 500’000 corms/ha
irrigation	- 808.9 m3 water/ha<br>- surface irrigation

processing steps	- harvesting (manual)<br>- plucking (manual)<br>- drying (shade drying)<br>- packaging for transport: cardboard boxes with aluminium layers<br>- packaging for sale: plastic bags of 5g per 6g content
transport mode to importing countries	- transport by lorry within iran<br>- air freight from iran to europe (4'314 km)<br>- general distribution in import country (not refrigerated)

9.3. Production sytem

Farming phase: Prior to cultivation, the land has to be prepared for corm multiplication by ploughing three to five times in May, June, and July and by fertilising the soil with 10 t ha−1 of manure (Menia et al., 2018). The corms are sown by hand from mid-August to mid-September after bed formation (Menia et al., 2018). Depending on the corm density, the amount of yield varies. Dense corms planting results in increased plants, bigger flowers and more yield because of the heavier stigmas (Andabjadid et al., 2015). According to Andabjadid et al. (2015) about 200 corms m2-1 are required for 170 kg ha−1 flower yield. A review on saffron cultivation recommends 2.5-3 t corms ha-1 or 500'000 corms ha-1 (Kumar et al., 2008).

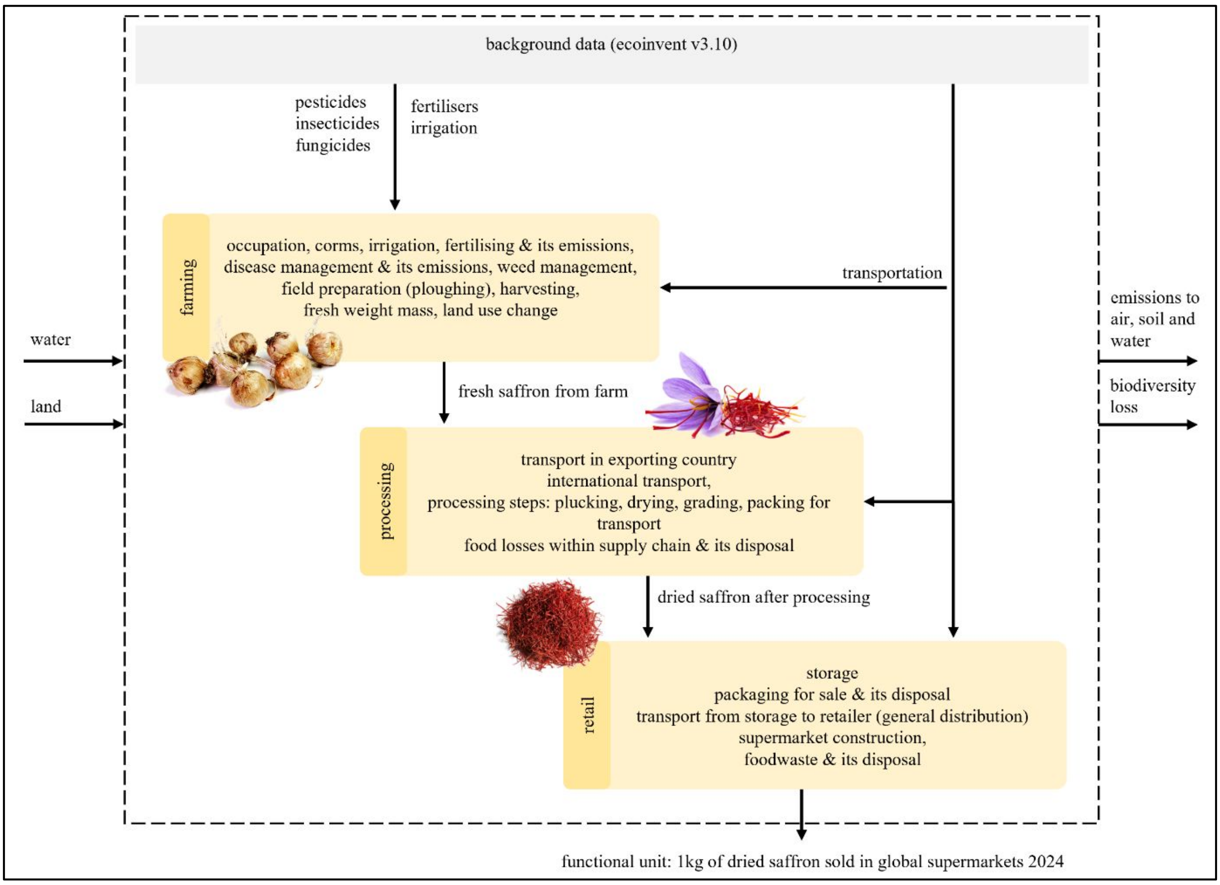

Figure 9: production system of 1 kg dried saffron sold in global supermarkets 2024.

The maximum yield of dried stigma found in a study on Indian Kashmir saffron is 4.8kg/ha after 4 to 5 years (Nehvi et al., 2014). The average yield of a recent study in Iran lays at 3.53 kg dried saffron/ha (Koocheki et al., 2019). The yield for Iran aligns with the production volume per ha stated in the study

of Cardone et al. (2020) which lays at 3.45kg/ha on average. Therefore, an average yield of 3.5 kg/ha is used for further calculations. Rotating the saffron cultivation increases yield and decreases pests incidents and diseases (Kafi et al., 2018). Intercropping with nitrogen fixating crops like chickpea, a sustainable cultivation management can be achieved (Asadi et al., 2016). Normally, a combination of liquid cow manure and urea as a nitrogen source is applied. The highest yield of saffron was achieved by using 20 t ha-1 of cow manure in combination with 50 kg ha-1 urea. The yield achieved was 0.45g m-2 which corresponds to 4.5kg ha-1 (Mohammad, 2012). This amount aligns with the range of farm yard manure use in Iran in general which lays between 10-80t ha-1 (Ghorbani & Koocheki, 2017). Saffron can grow in arid or semi-arid regions as the corms are dormant for about five months between May to September (Kafi et al., 2018). Prior to flowering, irrigation in autumn is necessary depending on the precipitation amount. The most used irrigation method for saffron cultivation is the basin irrigation which is a common form of surface irrigation (Mollafilabi et al., 2021) and has an irrigation efficiency factor of 0.45 (Nemecek et al., 2023). Controlling weed while cultivating saffron is either done by hand or by using herbicides like Atrazine (Gesaprim 50%) or Simanzine (Gesatop 50%). Each of these herbicides is applied with 1kg ha-1 saffron cultivation (Dar et al., 2017). Atrazine is used for the modelling, because of its availability in the ecoinvent database. Gesaprim 50% consists of 500g Antrazine per kg Gesaprim (Syngenta, 2018). As soon as the flowers appear on the fields, which is approximately 40 days after sowing, their plucking by hand starts (Kafi et al., 2018). The harvesting takes place from October to November in Khorasan, which is the main producing area in Iran (Husaini et al., 2010). For the yield of 1 kg dry saffron stigmas, about 78.5 kg fresh harvested flowers (about 170’000 flowers) are required (Husaini et al., 2010) which aligns with the 80% weight loss reported in a recent study on dehydration methods of saffron (Moratalla-López et al., 2021).

Processing phase: After harvesting, the flowers and stigmas are first divided by hand and then dried (Husaini et al., 2010). The drying either happens under the shade for 2-3 days (Husaini et al., 2010), or by freeze drying, vacuum drying, microwave drying, infrared drying, or oven drying (Chen et al., 2020). Storing saffron corms at 25°C resulted in higher yields as concluded by Hyjyzadeh et al. (2017). Since saffron is a light-sensitive cargo, it is important to protect it from light and moisture during the transport. Therefore, saffron is either packed in cans and boxes or in corrugated board cartons with aluminum coating (TIS, 2024). For this study the latter packaging is used. The stowage factor of saffron is 1.98m3/t (TIS, 2024) and therefore 1980 cm3 space is needed per kg saffron. This corresponds to a cardboard box of 20cm*12.5cm*8cm (2000 cm3). Cardboard has a density of 0.13 kg/m2 and is usually 0.25mm thick. Considering this information, the weight of carboard box is 13.26 g (1020 cm2 (= 0.102 m2) area with 0.025 cm thickness. 1m2 = 0.13 kg, 0.102m2 = 0.01326 kg = 13.26 g). The thickness of the aluminium layer is thin, around 0.006 mm. According to data from international trade centre, Iran is the country exporting the most and Spain, the one importing the most (ITC, 2022). Therefore, the transport distance is assumed to be from Teheran to the European area. The most used transport mode is air freight to ensure quick delivery, keep the valuable commodity save and, to preserve the quality of the saffron

by minimizing the time spent in transit (Pack, 2020). Based on SeaRates, this corresponds to a flight of 4’314 km (SeaRates, 2024).

Retail phase: The packaging in the supermarket mainly consists of flat letter-like plastic packaging (5 g) for 6 g saffron.

10. Turmeric

10.1. General Information

Turmeric (*curcuma longa*) is a commercially grown spice native to Southeast Asia and mainly used for cosmetic, medicinal, or culinary purposes (Quelle). The main producer of turmeric is India, where major regions are Telangana, Maharashtra, Karnataka, Assam and some more (Darekar et al., 2021). Problems with conventional production are the usage of agrochemicals which end up in the soil and also the rhizome which is consumed by the customers (Darekar et al., 2021). Currently, no official data on production volumes of turmeric exist in the FAO database, but their estimation is 938’955 t in 2019-2020 with India being the main producer, followed by Pakistan, China, Malaysia, and Tanzania (FAO, 2021).

Table 19: production amount and production countries of turmeric.

production amount	estimation is 938’955 t in 2019-2020 → 338’023 t dried and fresh product
producing countries	India: 80%, China: 8%, Myanmar: 4%, Nigeria 3%, Rest: 5% https://vtpc.karnataka.gov.in/storage/pdf-files/Turmeric%20hig%20res.pdf

10.2. Inventory data

Two different case studies are modelled in this study:

1) Organic production in Madagascar with primary data from the organic spice provider, grounded turmeric
2) Conventional production in India with literature values, fresh turmeric

Table 20: inventory data of turmeric.

models	organic model (1) - dried turmeric	conventional model (2) - fresh turmeric
country	Madagascar	India
sources	organic spice provider 2024	(Darekar et al., 2021)
Land use transformation	20y: 0.4 kg/kg yield<br>50y: 1.23kg/kg yield	20y: 0.09 kg/kg yield<br>50y:
yield	10’000 kg fresh turmeric/ha<br>Weight loss of 80%<br>2000 kg dried turmeric/ha	17’700 kg fresh rhizomes/ha<br>No weight loss for fresh turmeric
Occupation of land	Annual crop, 9 months cycle	Annual crop, 9 months cycle
fertiliser	Organic manure (3 kg/Are → 300 kg/ha)	NPK fertiliser: 200 kg N/ha, 100 kg P/ha, 100 kg K/ha
pesticides/ fungicides/ insecticides	None	- Leaf blotch & leaf spot & leaf blight disease: macozeb 0.2% at fortnight intervals. → 750g/kg mancozeb active substance, application rate: 1.7kg/ha, two applications = 3.4 kg/ha.<br>- Rhizome rot: copper oxychloride 0.25% → 2.5kg/ha (500g/kg active

		substance) → 1.25 kg active substance/ha<br>- Shoot borer: Belt® 480 SC Insecticide: active ingredient flubendiamide 480 g/L → 75ml/ha application → 36 g active substance/ha
Ginger rhizome seedlings	1000 kg/ha<br>0.1 kg/kg yield	1500-2000 kg/ha<br>0.074 kg/kg yield
irrigation	none	drip irrigation<br>7907.24 m3/ha (calculated value)
Processing steps	- Cleaning (water 0.001 m3/kg yield) → manual<br>- slicing, drying, sorting → manual<br>- packing into plastic bags<br>- disinfection & grinding in Hamburg<br>- packing in glass bottles	- Harvesting: by tractor<br>- Cleaning & washing: 0.001 m3/kg yield<br>- Cut into pieces: manual<br>- polishing & smoothened<br>- colouring<br>- grading<br>- packing in glass bottles
transport mode to importing countries	by lorry from mananara to tamatave (1200km)<br>by ship from tamatave via colombo to hamburg (17'300 km)<br>general distribution in importing country	By lorry from field to processing location<br><br>transport from main production country India to main importer by ship<br><br>general distribution in importing country
Important	Economic allocation necessary	

10.3. Production sytem

10.3.1. Conventional

Farming phase: planting time varies in regions, in India its between May and July (Darekar et al., 2021).Turmeric is planted into holes in ridges, where the spacing between ridges ranges from 45-60 cm (Darekar et al., 2021; FAO, n.d.) and between rows it is recommended to have 25 cm (Darekar et al., 2021) space in between. In case of planting in raised bed systems the spacing between rows can be decreased to 30 cm (Darekar et al., 2021; FAO, n.d.). In case of planting mother rhizomes, 2000-2500 kg ha-1 are needed and only 1500-2000 kg ha-1 for finger rhizomes (Mirjanaik & Vishwanath, 2020). Using mother rhizomes (50-60g weight) resulted in the highest yields (Angami et al., 2017). Irrigation is crucial and should be carried out by drip irrigation to avoid unnecessary water loss. Drip irrigation promotes turmeric growth best compared to other systems (Darekar et al., 2021). Depending on the soil, different amounts of irrigation are recommended with a quantity of 150-225 m3 water per ha. For clay soil its about 15-23 times and for sandy loams 40 times (FAO, n.d.). Fertilising differs depending on the soil composition and timing in application (FAO, n.d.). As turmeric can grow on different soil types like clay loam or sandy loam soil the needs differ. Turmeric likes high organic matter and a pH ranging from 4.5 to 7.5 (Darekar et al., 2021). The general recommendation is to fertilise four times; at planting, after 75 days after planting, after 120-130 days after planting, and after150 days after planting (FAO, n.d.). To increase the nutrient uptake hilling is recommended after 45-90 days and after 120 days after planting

(FAO, n.d.). Depending on the region, different NPK amounts are required. A randomized block design split plot study in Nigeria concluded that 150-300 kg fertilizer in 15 – 15 – 15 ration supports high yield the best (Ojikpong, 2018). In Pakistan an NPK ratio of 180:280:80 kg ha-1 to reach the highest yields is promising. For the region Maharashtra in India, which is one of the main producing areas (Darekar et al., 2021), the NPK ratio recommended for the highest yield is 200:100:100 kg NPK per ha in total, applied at four different times (Darekar et al., 2021) like recommended by the FAO (n.d.). There exist differences in the ratio and amount of NPK application between countries and regions. Therefore, the ratio of the cultivation in the region producing most turmeric worldwide is chosen, which is Maharashtra in India (Darekar et al., 2021).

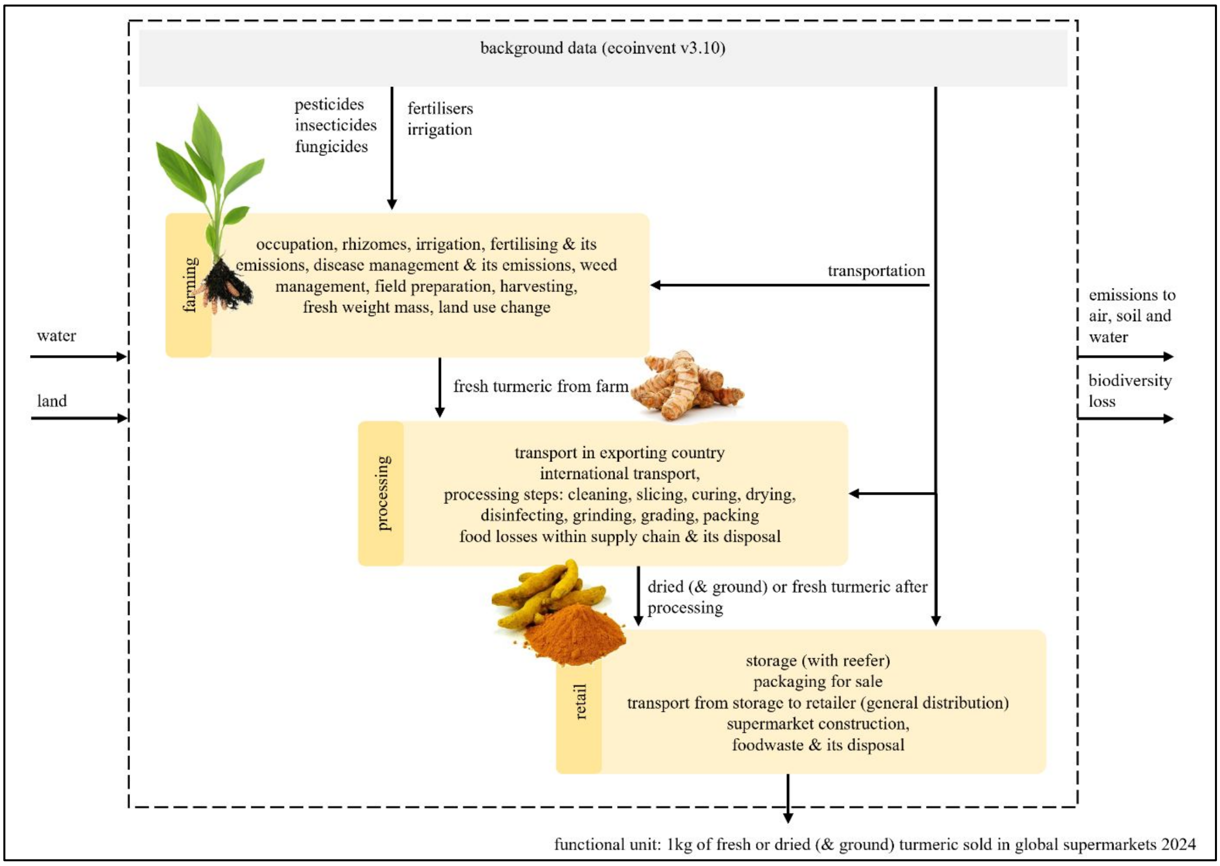

Figure 10: production system of 1 kg fresh or dried (& ground) turmeric sold in global supermarkets 2024.

Mulching immediately after planting controls weed in combination with weeding three times during the growing season (after 60, 90 and 120 days) (FAO, n.d.). Especially in organic farming, weeds are challenging (Darekar et al., 2021). There exist different practices which help to control weed such as tillage, crop rotation, intercropping, increasing plant density, or rapid cleanup after harvest (Darekar et al., 2021). Turmeric can be infested with shoot borer insect or the rhizome scale insect. In case of the shoot borer insect, only pesticides can prevent further spreading. The rhizome scale insect, however, infests matured rhizomes which is why it is crucial to harvest rhizomes without delay. Those rhizomes used for seed material are first treated with insecticides (20-30min) before storage. Also diseases like the rhizome rot disease, leaf spot disease or the leaf blotch disease are a threat to turmeric. When the turmeric emits a foul odour it might be rhizome rot disease, caused by Fusarium or Pythium. By applying

biocontrol based on microbes like Trichoderma, Pseudomonas or Bacillus twice, as seed treatment and soil application after 30-45 days after planting, this disease can be prevented. Also leaf spot disease, showing dark lesions and yellow halo around this spot, can lead to yield losses. The leaf blotch disease shows square shaped lesions in combination with small spots (FAO, n.d.)

According to the Indian Institute of Spices Research several insecticides can be applied. For this model following three insecticides are assumed. Against leaf blotch, leaf spot and leaf blight disease mancozeb at 0.2% is applied. Per 1kg insecticide, 750 g active substance is applied wit a rate 0f 1.7 kg/ha, twice. For the rhizome rot disease copper oxychloride (500g active substance per kg) with an application rate of 2.5kg/ha is assumed. And for fighting the shoot borer, an insecticide with 480 g active ingredient flubendiamide 480 /L is modelled with an application of 75ml/ha application (Prasath et al., 2022).

After seven to nine months, turmeric reaches its maturity which can be told by its light brown and yellowing leaves (FAO, n.d.). Harvesting can then be done by hand or by using a tractor, for example a power tiller operated turmeric harvester (FAO, n.d.). The largest part (90%) is further processed into powder, essential or turmeone oil. Turmeric is sold fresh or in powder as a spice.

Processing phase: after harvesting the rhizomes are washed using tap water, cut into smaller pieces (fingers or chips) The fresh turmeric goes trough cleaning, curing, polishing colouring and grading. Curing means boiling it in hot water for 40 minutes followed by drying them for 11 days. Afterwards the rhizomes are polished and smoothened by rubbing which can be done manually or mechanically. Lastly, the rhizomes are graded into fingers (ranging from 2.5cm to 7.5cm), into bulbs (mother rhizomes) and into splits (split mother rhizomes) (Darekar et al., 2021).

10.3.2. Organic

Farming phase: Turmeric is grown for 9 months in an intercropping system with crops like cassava (7’000kg/ha), pineapple (50’000kg/ha), litchi (5’000kg/ha), or banana (30’000kg/ha) and yields in 10’000kg fresh turmeric/ha. Since different crops are cultivated, an economic allocation is performed. The exact market price of these products will remain confidential, therefore only the price relation of 1:12 (bananae:turmeric) is documented. These allocation factors are included in the farming stage, meaning that 80 % of the emissions are allocated to turmeric, whereas the remaining 20 % are allocated to bananas. The extensive cultivation avoids pesticides, mineral fertilizers, and irrigation and relys on manual labor. The only input during the farming stage is organic manure from cattle. Harvesting is performed manually, and the turmeric is transported on a man’s back to the nearest processing facility. Since the leaves of the turmeric plant are left on the field, an estimation of its biomass is necessary to model its humification.

Processing phase: processing the fresh turmeric involves cleaning, slicing, drying, sorting, and packing. At the facility, the turmeric is washed using tap or river water where no pumps are needed. The tropical climate ensures an ample water supply, preventing issues with water scarcity. After washing, the turmeric is sliced and sun-dried until it becomes completely dry chips. During the drying process, 80%

of its original weight is lost. These chips are then packed into 25 kg bags composed of 95% polypropylene, 4% calcium carbonate, and 1% paraffin. (Calculation: density of ginger: 88g/236.6cm3 → volume for 25 kg is 236.6/88*2500 = 6727.3cm3 =6.73l content of ginger chips. Add some air, since it is not ground but in chips form = 8l = 8000cm3. Plastic bag this size is around 15g). The packed turmeric chips are transported by lorry to Tamatave and then shipped to Hamburg via Colombo. In Hamburg, the chips are stored until they are requested by consumers (assumption of an average storage time of 1 month), either in chips form or as ground turmeric. In case of pests, pressure disinfection is necessary which is the case in 5% of the amount. If no manifestation with pest, only steam sterilisation (autoklav) is applied with temperatures of 70-105°C. For the ground turmeric, its ground in a mill in Hamburg wich is cooled.

Retail phase: the ground turmeric is then packaged in glass jars, with each jar weighing about 85 grams for 40 grams of turmeric powder. The jars are subsequently transported and sold in stores.

11. Vanilla

11.1. General Information

Vanilla is a widely favored flavor across food, perfumery, and pharmaceutical sectors, which originates from Mexico (Lubinsky et al., 2006). The botanical genus Vanilla, classified under the Orchid family, includes *Vanilla planifolia Jacks ex. Andrews*, the predominant species utilized for natural vanilla production derived from its fruits (Arenas & Dressler, 2009). Aerial roots of vanilla require support from other plants like avocado or coffee or artificial structures such as wood and concrete systems (Chambers et al., 2019). Vanilla plants can grow up to 60 m and thrives in warm and humid conditions with rainfalls between 170 and 280 cm a year (Chambers et al., 2019).

Table 21: production amount and production countries of vanilla.

production amount	7’704 tons in 2022 (FAOstat, 2022)
producing countries	Madagascar (39.4%), Indonesia (25.5%), Mexico (9.2%), Papua New Guinea (6.4%), China (5.6%), Rest (13.9%) (FAOstat, 2022)

11.2. Inventory data

Two different case studies are modelled in this study and the ecoinvent database of global vanilla production is used as a reference.

1) Organic production in Madagascar with primary data from the organic spice provider
2) Conventional production in Mexico with literature values and primary data from previous studies

Table 22: inventory data of vanilla.

studies/data sources	reference organic (0)	organic (1)	conventional (2)
country	global	madagascar, mananara nord	mexico, veracruz & puebla
sources	ecoinvent vanilla {glo}	organic spice provider 2024	lupi 2023 & stamm 2024
Land use change		20y: 0 or -0.190462048<br>50y: 14.1	20y: 0
yield	750 kg green vanilla /ha	- 1200 kg green vanilla /ha<br>- Weight loss of 75%<br>- 300 kg fermented vanilla /ha	- 350 kg green vanilla /ha<br>- Weight loss of 83%<br>- 59.5 kg fermented vanilla /ha
lifetime of perennial crop	20 years	25 years	25 years
fertiliser	none	none	NPK, ammonium sulfate, calcium carbonate, calcium nitrate, ash, manure, compost
pesticides/ fungicides/ insecticides	none	none	general pesticides
vanilla seedlings	0.18 pieces per kg fermented vanilla	0.04 pieces/ kg green vanilla	0.137 pieces/ kg green vanilla

	1500 seedlings/ha	1200 cuttings/ha	
irrigation	none	none	185.22 m3/ha, surface irrigation
Processing steps	not further explained	- scalding, steaming, sun drying, sorting, grading, shade drying → manual<br>- Packaging transport: in plastic bags & carton boxes<br>- Packaging sale: glass bottles	- scalding, steaming, sun drying sorting, grading, shade drying, packaging<br>- packaging transport: cotton bags<br>- Packaging sale: glass bottles
transport mode to importing countries	sea and lorry freight	- air freight (17'700 km)<br>- general distribution in import country (refrigerated)<br>- cooled storage	- lorry cooled<br>- sea fright (8'100 km)<br>- general distribution in import country (refrigerated)<br>- Cooled storage
Important			

11.3. Production sytem

11.3.1. Conventional

Farming phase: vanilla can be propagated either by sexual propagation or by stem cutting which can lead to spreading diseases like root and stem rot (*Fusarium oxysporum*), the red bug (*Tenthecoris confuses*), and anthracnosis which all lead to yield loss and therefore financial loss (Ramos-Castellá et al., 2014). Sexual propagation on the other hand often shows no germination from seeds. Therefore, applying micropropagation promises the best results (Ramos-Castellá et al., 2014). Its pollination is primarily reliant on hymenopteran species, such as bees which are only present in middle America. In regions lacking these species, manual pollination becomes necessary, rendering vanilla cultivation among the most labor-intensive and time-consuming agricultural endeavors globally (Lubinsky et al., 2006).

The yield per ha varies depending on production system and producing countries. A study of Hernandez (2019) in Mexico showed that 90% of all farmers produced vanilla in an extensive agroforestry system where patches were smaller than 1 ha. The yield of green vanilla lays between 50 and 500 kg per ha with an average of 200 kg fermented vanilla per ha (Hernández, 2019). Lupi (2023) concluded an average yield of 131.52 kg green vanilla per ha comparing 26 different farmers in Mexico. For this study 350 kg/ha is used. Vanilla plants and tutor plants are ideally replaced every 11 to 20 years to maintain a high harvest (Lupi, 2023; Talattad et al., 2021). Since most of the cultivation of vanilla is done manually and without machinery, the inputs during cultivation are low (Lupi, 2023; Talattad et al., 2021). After vanilla is grown for six to eight months, it can be harvested.

Processing phase: immediately after harvesting, the fermentation process is initiated by briefly heating green vanilla pods in water until it reaches 60°C. Subsequently, they are wrapped in plastic foil and cotton bags. Following fermentation, the pods are sun-dried for two hours daily for three weeks on

wooden structures to prevent mold growth and then stored in a transpiration box for another three months (Talattad et al., 2021). The fermentation process, which only begins through the alternating effects of heat, moisture and air extraction, ultimately gives the fruit its characteristic flavour and dark colour. Afterwards the vanilla pods can be transported to all the countries where they are purchased. Transport is modelled as explained in Chapter 13.

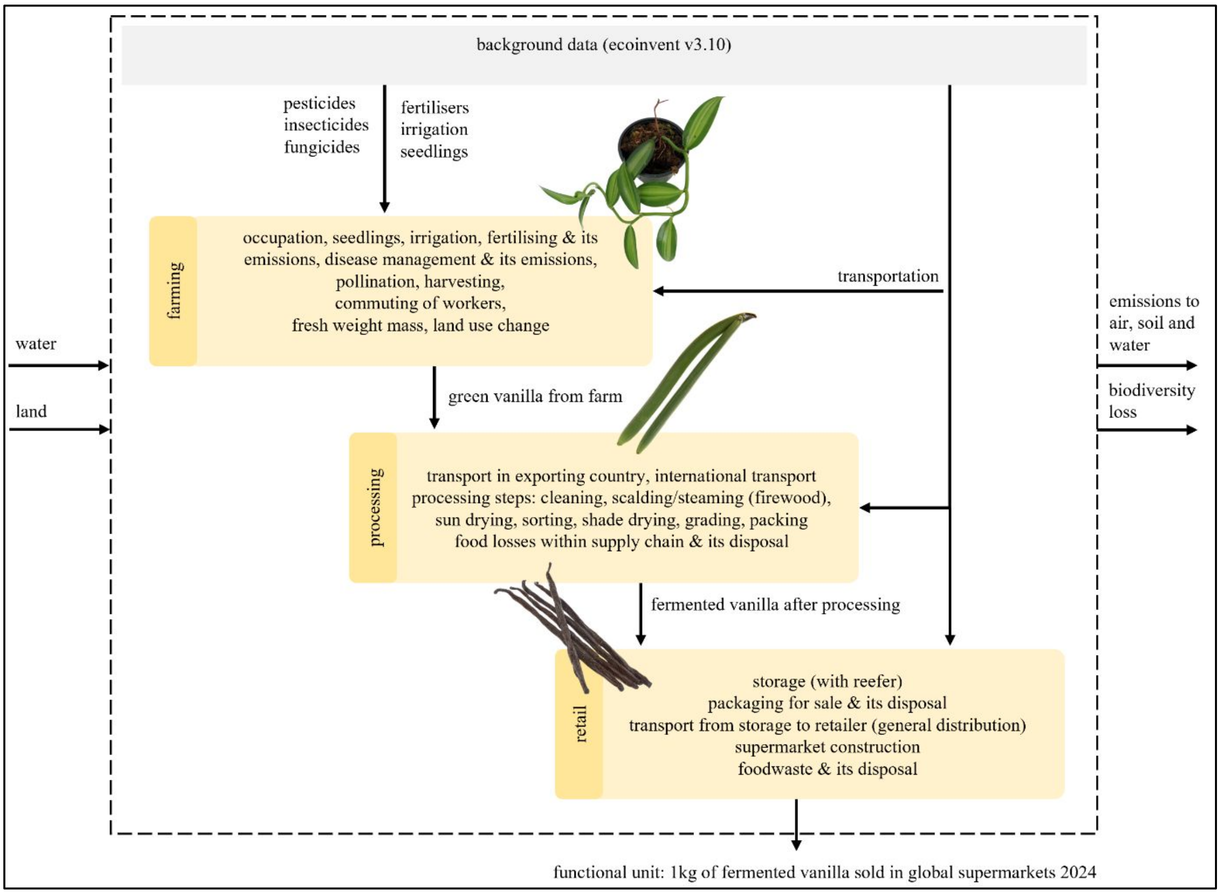

Figure 11: production system of 1 kg fermented vanilla sold in global supermarkets 2024.

11.3.2. Organic

Farming phase: vanilla is grown in an agroforestry system in Mananara North, Madagascar, and yields 1200 kg of green vanilla per hectare. The cultivation is completely organic, without irrigation, fertilisers, pesticides or other aids. Harvesting and processing is done manually and without machines. The vanilla plants are supported either by poles or existing plants within the agroforestry system. The processing of the vanilla involves several steps starting with scalding, steaming, drying in the sun, then sorting, grading, drying in the shade and packaging. Only water and heat are required for the steaming process. During the sun-drying process, vanilla loses 75 % of its weight. The vanilla is packed into smaller plastic bags which are then put into carton boxes holding each 19 kg of vanilla. The transport within Madagascar to a warehouse in Antananarivo is by plane and from there it is shipped as air freight to Switzerland. This method is chosen for insurance reasons, as vanilla transported in shipping containers cannot be insured at the same rate as air freight. In addition, the insecticides that are sprayed on the containers to prevent the spread of diseases and pests pose a risk of contamination for the organic crops.

12. Packaging for transport retail

Two distinct types of packaging are considered in the analysis: transportation packaging, which is accounted for in the processing phase, and retail packaging, which is included in the retail phase.

Packaging for transport: The packaging for transport depends on the case study. There are different materials used and modelled as displayed in table X. The entire packaging for transport, including disposal, is considered as part of the processing phase.

Table 23: packaging material and disposal datasets used from ecoinvent.

Packaging material	Disposal
Plastic bag: Packaging film, low density polyethylene {GLO}| market for packaging film, low density polyethylene | Cut-off, U	Waste polyethylene {RER}| market group for waste polyethylene | Cut-off, U
Carton: Folding boxboard carton {RER}| market for folding boxboard carton | Cut-off, U	Waste paperboard {RER}| market group for waste paperboard | Cut-off, U
Mixture: Polypropylene, granulate {GLO}| market for polypropylene, granulate | Cut-off, U, Paraffin {GLO}| market for paraffin | Cut-off, U, Calcium carbonate, precipitated {RER}| calcium carbonate production, precipitated | Cut-off, U	Municipal solid waste {RER}| market group for municipal solid waste | Cut-off,
Yute bag: Waste yarn and waste textile {GLO}| market for waste yarn and waste textile | Cut-off, U	Waste yarn and waste textile {RoW}| market for waste yarn and waste textile | Cut-off, U
Cotton bag: Fibre, cotton {RoW}| fibre production, cotton, ginning	Municipal solid waste {RER}| market group for municipal solid waste | Cut-off, U

Retail packaging: Spices are commonly sold in uniform glass bottles, making it important to understand the ratio of glass to spice. In this study, spices packaged in glass bottles from Migros were analysed. Two standard bottle sizes were considered: one weighing 100 grams, with dimensions of 12 cm in height and 4.5 cm in diameter, and the other weighing 166 grams, with the same height but a diameter of 6 cm. The quantity of spice contained within each bottle varies based on the type of spice and whether it is whole or ground. For the sale in the supermarket, the number of bottles or packages per m^3 is calculated.

- 100g-bottles: on 45cm*45cm*18cm (=36’450 cm^3 = 0.03645 m^3) are 100 bottles (diameter of 4.5 cm) → 2’743 bottles/m^3. To include the floor construction, the number of bottles is halved to **1’371 bottles/m^3.**
- Plastic packaging: on 45cm*45cm*18cm (= 0.03645 m^3) are 166 packages → 4554 packages/ m^3. To include the floor construction, the number of packages is halved to **2’277 packages/m^3**.
- 166g-bottle: on 45cm*45cm*18cm (= 0.03645 m^3) are 56 bottles (diameter of 6 cm) → 1536 bottles/m^3. To include the floor construction, the number of bottles is halved to **768 bottles/m^3.**
- Vanilla-glass: on 45cm*45cm*18cm (= 0.03645 m^3) are 200 bottles → 5485 bottles/m^3. To include the floor construction, the number of bottles is halved to **2’742 bottles/m^3.**
- **Saffron-packages:** on 45cm*45cm*18cm (= 0.03645 m^3) are 225 packages. → 6172 packages/ m^3. To include the floor construction, the number of packages is halved to **3’086 packages/m^3**.

For modeling the packaging, data were utilized on glass (Packaging glass, white {GLO} | market for packaging glass, white | Cut-off, U), the plastic material used for the caps (Polyethylene terephthalate, granulate, bottle grade {GLO} | market for polyethylene terephthalate, granulate, bottle grade | Cut-off,

U), and its forming process (Thermoforming of plastic sheets {GLO} | market for thermoforming of plastic sheets | Cut-off, U). Since the bottles are disposed of after use, waste treatment processes were also included (Waste plastic, mixture {RoW} | treatment of waste plastic, mixture, municipal incineration | Cut-off, U and Packaging glass, white (waste treatment) {GLO} | recycling of packaging glass, white | Cut-off, U). The entire packaging process, including disposal, is considered as part of the retail phase.

Table 24: Packaging details for retail phase for every analysed spice.

**spice & form**	**packaging [g]**	**spice [g] per packaging**	**spice per m3**	**plastic per kg spice**	**glass per kg spice**
turmeric – dried & ground in glass bottles	bottle: 100 lid: 6	55	1371*55 g = 75’405 g	1000/55*6 = 109 g	1000/55*100 = 1818 g
turmeric – fresh	none		75.405 kg → 20% less per m^3=60324 kg		
ginger – dried & ground in glass bottles	bottle: 100 lid: 6	37	1371*37 g = 50’727 g	1000/37*6 = 162 g	1000/37*100 = 2702 g
ginger – fresh	none		50.727 kg → 20% less per m3		
cinnamon – dried & ground in glass bottles	bottle: 100 lid: 6	45	1371*45 g = 61’695 g	1000/45*6 =133 g	1000/45*100 = 2’222 g
cinnamon – dried & quills in plastic packages	plastic: 3	40	200*40 = 8000 g	1000/40*3 = 75 g	0g
cardamom – dried & whole	bottle: 100 lid: 6	32	1371*32 g = 43’872 g	1000/32*6 = 187 g	1000/32*100 = 3’125 g
cloves– dried & ground	bottle: 100 lid: 6	45	1371*45 g = 61’695 g	1000/45*6 =133 g	1000/45*100 = 2’222 g
cloves – dried & whole	bottle: 100 lid: 6	33	1371*33 g = 45’243 g	1000/33*6 = 182 g	1000/33*100 = 3030 g
vanilla – fermented	bottle: 11 lid: 2	7	2742*7 g = 19’194 g	1000/7*2 = 286 g	1000/7*11 = 1571 g
pepper – dried & ground	bottle: 100 lid: 6	50	1371*50 g = 680550 g	1000/50*6 = 120 g	1000/50*100 = 2000 g
pepper – dried & whole	bottle: 166 lid: 5	100	768*100 = 76800 g	1000/100*5 = 50 g	1000/100*166 = 1660 g
pepper – dried & whole	plastic: 3	95	2277*95 g = 216315 g	1000/95*3 = 32 g	0 g
coriander seeds – dried & ground	bottle: 100 lid: 6	40	1371*40 g = 54840 g	1000/40*6 = 150 g	1000/40*100 = 2500 g
cumin seeds – dried & whole	bottle: 100 lid: 6	47	1371*47 g = 64437 g	1000/47*6 = 128 g	1000/47*100 = 2128 g
cumin seeds – dried & ground	bottle: 100 lid: 6	50	1371* 50 g = 68550 g	1000/50*6 = 120 g	1000/50*100 = 2000 g
saffron – dried & whole	plastic: 5	6	3068*6 g = 18518 g	1000/6*5 = 833 g	0 g

paprika – dried & ground	bottle: 100 lid: 6	57	1371*57 g = 78147 g	1000/57*6 = 105 g	1000/57*100 = 1754 g
paprika – dried & ground	bottle: 166 lid: 5	95	768*95 = 72960	1000/95*5 = 53 g	1000/95*166 = 1747 g
paprika – dried & gound	plastic: 3	95	2277*95 g = 216315 g	1000/95*3 = 32 g	0 g
nutmeg – dried & whole	bottle: 100 lid: 6	38	1371*38 g = 51'098 g	1000/38*6 = 158 g	1000/38*100 = 2632 g
nutmeg – dried & ground	bottle: 100 lid: 6	50	1371* 50 g = 68'550 g	1000/50*6 = 120 g	1000/50*100 = 2000 g
Mace – dried & whole	Plastic: 5	40	200*40 g = 8'000 g	1000/40*5 = 125 g	0 g

For the retail in a global supermarket, it is assumed that dried spices stay in the supermarket for one week, whereas fresh spices like ginger stay for five days. The dataset {Building, multi-storey {GLO}| market for building, multi-storey | Cut-off, U} of ecoinvent (ecoinvent v3.10, 2024) is used to model the supermarket construction.

13. Transport distances

This study simplifies the modelling of trade routes by assuming standardized **transport distances** rather than mapping exact routes for global spice production. Three distinct types of transport are considered: **(1) Transport distances within production country**: modelled case-specific, in absence of case-specific data, an average distance of 1’200 km/kg of spice by lorry is assumed. Following datasets are used for transport in production countries: *Transport, freight, lorry, unspecified {GLO}| market group for transport, freight, lorry, unspecified | Cut-off, U*

(2) International transport from the exporting to the importing country: is modelled using data from the International Trade Centre (ITC, 2022) to identify major producing and importing countries. Based on the world region (Figure 12) of the primary importing country (World Bank, 2018), transport distances are calculated using the Cargo Calculator from SeaRates (2024). The distance is measured from the capital city of the leading producing country to the largest trading hub in the world region importing the highest quantity. The designated trading hubs are Amsterdam for Europe (Stafford, 2021), the Port of Los Angeles for North America (GoComet, 2024), and Singapore for East Asia and the Pacific (HSBC, 2024). Exceptions are these case studies, where importing and exporting country are clearly stated. Table 25 shows all transport distances and modes used for the modelling of the spices.

Table 25: Transport distances in exporting countries (1), international transport (2), and transport within importing countries (3) for every analysed spice.

Spice	Transport in exporting country (1)	International transport (2)	Transport in importing country (3)
Vanilla, organic	By motorbike (30 km)<br>By air freight (300 km) from Mananra to Antananarivo	By air freight from Antanarivo to Hamburg (8900 km)	by train, refrigerated (1552 km), 11.6 w%<br>by lorry, refrigerated (541 km), 89.7 w%
Vanilla, conventional	By lorry, refrigerated (300 km)	By sea freight from Mexico to Los Angeles (2470 km)	by train, refrigerated (1552 km), 11.6 w%<br>by lorry, refrigerated (541 km), 89.7 w%
Ginger, organic	By lorry (not refrigerated) (1200 km)	By sea freight from Madagascar to Hamburg (17300 km)	by train, refrigerated (1552 km), 11.6 w%<br>by lorry, refrigerated (541 km), 89.7 w%
Ginger, conventional	By small lorry, not refrigerated (50 km)<br>By lorry, refrigerated (416 km)	By sea freight from India to Amsterdam (12150 km)	by train, refrigerated (1552 km), 11.6 w%<br>by lorry, refrigerated (541 km), 89.7 w%
Cinnamon, organic	By lorry (not refrigerated) (1200 km)	By sea freight from Madagascar to Hamburg (17300 km)	by train, refrigerated (1552 km), 11.6 w%<br>by lorry, refrigerated (541 km), 89.7 w%
Cinnamon, conventional	By lorry (not refrigerated) (400 km)	By sea freight from India to Los Angeles (15000 km)	by train, refrigerated (1552 km), 11.6 w%<br>by lorry, refrigerated (541 km), 89.7 w%
Cloves, organic	By boat (not refrigerated) (1200 km)	By sea freight from Madagascar to Hamburg (17300 km)	by train, refrigerated (1552 km), 11.6 w%

			by lorry, refrigerated (541 km), 89.7 w%
Cloves, conventional	By lorry (not refrigerated) (1200 km)	By sea freight from India to Singapore (2900 km)	by train, refrigerated (1552 km), 11.6 w% by lorry, refrigerated (541 km), 89.7 w%
Cumin, conventional	By lorry (not refrigerated) (1200 km)	By sea freight from India to Singapore (4160 km)	by train, refrigerated (1552 km), 11.6 w% by lorry, refrigerated (541 km), 89.7 w%
Turmeric, organic	By boat (not refrigerated) (1200 km)	By sea freight from Madagascar to Hamburg (17300 km)	by train, refrigerated (1552 km), 11.6 w% by lorry, refrigerated (541 km), 89.7 w%
Turmeric, conventional	By lorry (refrigerated) (1200 km)	By sea freight from India to Los Angeles (15000 km)	by train, refrigerated (1552 km), 11.6 w% by lorry, refrigerated (541 km), 89.7 w%
Saffron, conventional	By lorry (not refrigerated) (1200 km)	By air freight from Iran to Amsterdam (4300 km)	by train, refrigerated (1552 km), 11.6 w% by lorry, refrigerated (541 km), 89.7 w%
Black pepper, conventional	By lorry (not refrigerated) (1200 km)	By sea freight from Vietnam to Los Angeles (13300 km)	by train, refrigerated (1552 km), 11.6 w% by lorry, refrigerated (541 km), 89.7 w%
Cardamom, conventional	By lorry (not refrigerated) (1200 km)	By sea freight from India to Singapore (4160 km)	by train, refrigerated (1552 km), 11.6 w% by lorry, refrigerated (541 km), 89.7 w%
Nutmeg, mace, conventional	By lorry (not refrigerated) (1200 km)	By sea freight from India to Singapore (2900 km)	by train, refrigerated (1552 km), 11.6 w% by lorry, refrigerated (541 km), 89.7 w%
capsicum, conventional	By lorry (not refrigerated) (1200 km)	By sea freight from India to Los Angeles (15000 km)	by train, refrigerated (1552 km), 11.6 w% by lorry, refrigerated (541 km), 89.7 w%

Following datasets are used for transportation:

- *air freight, long haul: Transport, freight, aircraft, long haul {GLO}| market for transport, freight, aircraft, long haul | Cut-off, U*
- *air freight, short haul: Transport, freight, aircraft, short haul {GLO}| market for transport, freight, aircraft, short haul | Cut-off, U*
- *light commercial vehicle: Transport, freight, light commercial vehicle {RoW}| market for transport, freight, light commercial vehicle | Cut-off, U*
- *lorry, cooled: Transport, freight, lorry with reefer, cooling {GLO}| transport, freight, lorry with reefer, cooling*
- *lorry not cooled: Transport, freight, lorry, unspecified {GLO}| market group for transport, freight, lorry, unspecified | Cut-off, U*
- *sea freight: Transport, freight, sea, container ship {GLO}| market for transport, freight, sea, container ship*

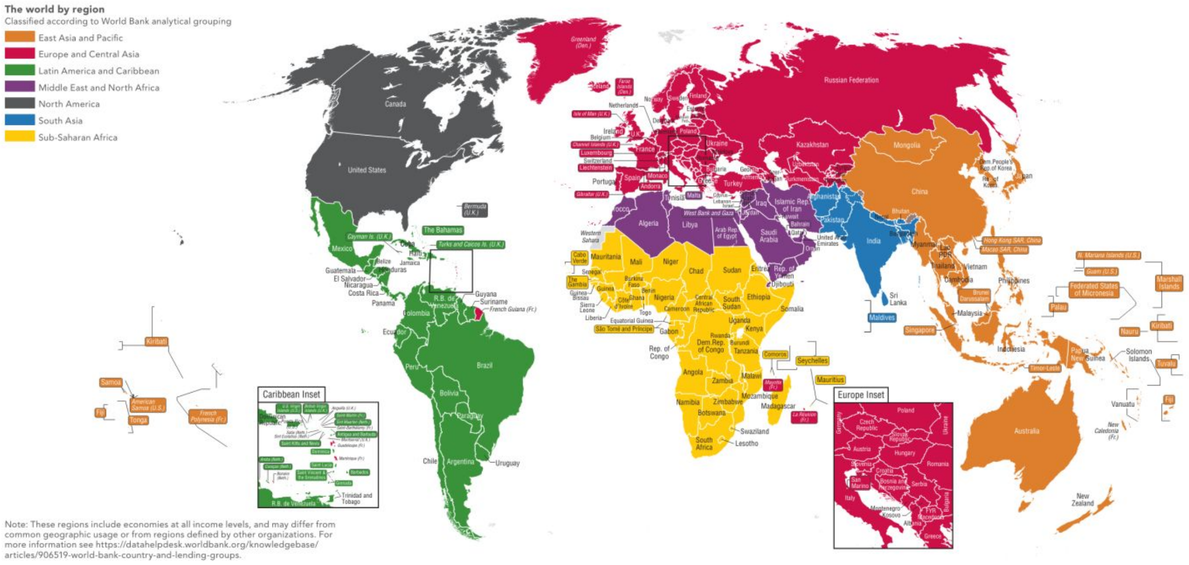

Figure 12: World regions used for the importing areas.

(3) Transportation within the importing region: transport distance assumptions are based on default data provided by Borken-Kleefeld (2012) through the ecoinvent center. These assumptions cover average transport distances per commodity group (in this case: agricultural products) for both the United States and the EU27. The provided data serve as recommended defaults for freight transport when specific transport details are missing (Borken-Kleefeld, 2012). The analysis includes not only the weight of the spice itself but also the weight of the packaging. Although the spices are dried and therefore less lowers the risk of microbial growth, the spices are transported in a cooled environment which also protects its aroma. For the spices, the category 06 of milled grained products is used where 89.7% of the weight is assumed to be transported by lorry for 541 km and 11.6 % of the weight by rail for 1552 km. Following datasets are used for transport in importing countries:

- *by lorry: Transport, freight, lorry with reefer, cooling {GLO}| transport, freight, lorry with reefer, cooling*
- *by train: transport, freight, train with reefer, cooling {glo}| market for transport, freight, train with reefer, cooling*
- *by sea freight: transport, freight, sea, container ship with reefer, cooling {glo}| market for transport, freight, sea, container ship with reefer, cooling*

14. Irrigation

Water use is modeled following the life cycle inventory guidelines for agricultural products (Nemecek et al., 2023). This includes water withdrawal, water emitted to air, surface and groundwater, and wastewater sent to treatment. For crop production, only irrigation water (excluding green water) is considered due to its environmental impact. The irrigation water withdrawal is calculated as:

$$I_{withdrawal} = \frac{ET_{Irr}}{EF_{Irr}}\left[\frac{m^3}{t}\right]; with\ ET_{Irr} = Evapotranspiration\ from\ irrigation\ \left[\frac{m^3}{t}\right]\ and\ EF_{Irr} = Irrigation\ efficiency\ fator$$

Default irrigation efficiency values are 0.45 for surface irrigation, 0.75 for sprinklers, and 0.9 for drip systems. Values for evapotranspiration from irrigation (blue water footprint) per crop in a specific country are obtained from Pfister et al. (2011) by using the arithmetic mean of the ideal water irrigation amount (upper boundary) and the water deficit (lower boundary). An example calculation of ginger in India is displayed. Further calculations are in the supporting material SI3.

Table 26: example calculation of irrigation amounts for the spice ginger from India.

**Spice**	**Evapotranspiration from irrigation [m3/t]**	**Irrigation efficiency factor**	**Water withdrawal [m3/t]**	**m3 Water/ha**
Ginger, India	(238.91+285.52)/2 → 262.215	Drip irrigation, 0.9	262.215/0.9 = 291.35	Yield: 10.7t/ha<br>291.35*10.7 =<br>**3117.445 m3/ha**

For easier comparison, Table 27 shows the irrigation amount per spice and ha.

Table 27: irrigation amount per in m³ per ha and per kg fresh yield.

**spice**	**irrigation water [m3/ha cultivation]**	**irrigation water [m3/kg yield]**	**type of irrigation**
capsicum, IN		2.81	mixture
cardamom IN	5334.38	5.33	sprinkler
cinnamon MG	1046.08	0.58	surface
cinnamon IN	290.58	0.58	
cloves IN	8190.45	1.28	surface
cloves MG	3285.42	1.28	
cumin etc. IN	1026.14	1.58	drip
ginger MG	3641.88	0.29	drip
ginger IN	3117.45	0.29	
nutmeg & mace IN	2844.82	1.35	mixture
pepper VN	444.02	0.18	sprinkler
saffron IR	808.90	2.95	surface
turmeric MG	29135.00	0.29	
turmerig IN	7907.24	0.29	drip
vanilla MX	185.22	1.41	
vanilla MG	1053.64	0.88	surface

15. Biodiversity assessment by the method Scherer et al. (2023)

For the assessment, the cultivation of all case studies had to be classified into the cultivation categories by Scherer et al. (2023). Spices grown in agroforestry or intercropping systems are assigned to cropland under minimal intensity. Monoculture spices with moderate fertilizer and pesticide inputs are associated with light-intensity cropland. Tree-grown spices, such as cloves, are classified under plantations with minimal or light intensity (further details in SI5).

Land use for seeds, wood, infrastructure, and other production inputs were excluded. The potential species loss was calculated based on the older version of Chaudhary et al. (2015) to estimate the uncertainty due to excluding these factors. The results from the Chaudhary and LUIF methods show similar trends (SI1 – Chapter 15). According to Chaudhary's method (2015), 98% of the biodiversity impact can be attributed to land use in farming, leaving a 2% margin of uncertainty from land use related to other inputs. The divergence between the two methods stems also from differences in characterization factors and geographical resolution: Chaudhary's method operates at the country level (at least if applied in Simapro), while LUIF applies an ecoregion.

As the assessment in the study, conducted with the Scherer et al. (2023) method only includes land use from cultivation and not from other inputs an analysis of all land use inputs by Chaudhary et al (2015) is conducted. The results are displayed in Table 28.

Table 28: Comparison of the results of a biodiversity assessment of Chaudhary et al. (2015) and Scherer et al. (2023). The orange marked numbers show the higher values.

Spice	Chaudhary et al. (2015)	Scherer et al. (2023)
capsicum conventional	8.3228E-15	2.93E-16
cardamom	1.316456E-13	6.49E-14
cinnamon conventional	3.1937259E-14	6.80E-14
cinnamon organic	2.4773036E-14	3.28E-13
cloves con	1.403325E-14	2.63E-14
cloves organic	1.7657299E-14	2.31E-13
cumin	1.1594589E-13	4.26E-15
ginger con	2.8553259E-15	1.22E-15
ginger organic	9.4288898E-14	4.96E-13
mace	2.9571668E-14	4.46E-13
nutmeg	1.6951035E-14	4.35E-14
pepper	4.4954436E-14	1.88E-13
saffron	3.7329956E-12	7.60E-13
turmeric con	1.5371255E-15	6.11E-16
turmeric organic	8.197736E-14	4.43E-13
vanilla con	9.7401574E-13	2.42E-12
vanilla organic	4.9649832E-13	2.95E-12

16. Visualisations EF3.1

16.1 Environmental impacts of spices (EF3.1) - normalized

Figure 13 shows the normalized results of the EF3.1 midpoint categories for all 12 spices.

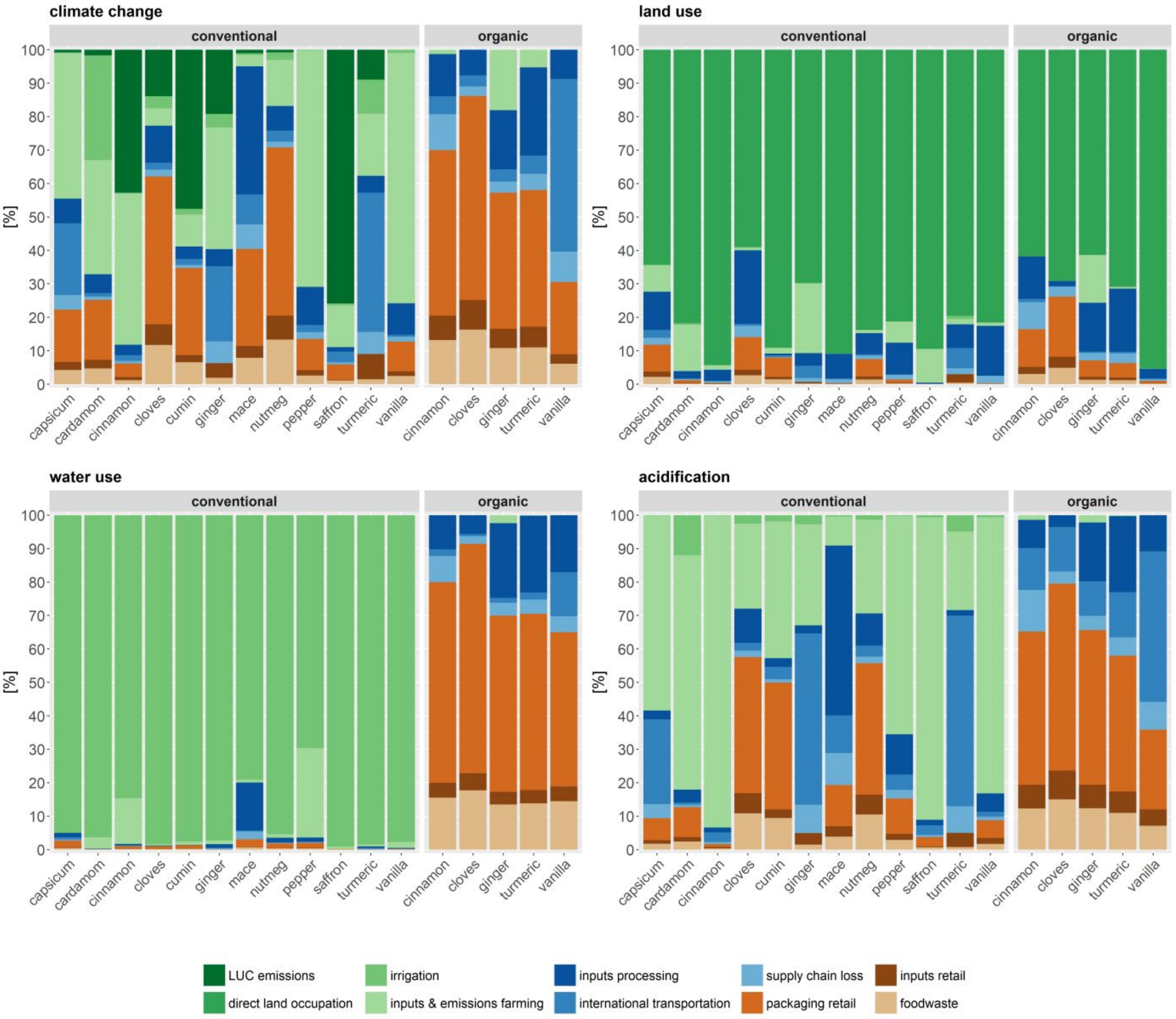

Figure 13:Normalized values of four midpoint impact categories of EF3.1 of 17 spices based on specific case studies. The process contributions are shown based on three stages: (1) cultivation: land use change emissions, occupation, irrigation, inputs & emissions farming (fertilizer, pesticides, seeds, cultivation methods); (2) processing: inputs processing (infrastructure, processes like drying), international transportation, supply chain loss; (3) retail: packaging retail, inputs retail (infrastructure, etc.), foodwaste. Conventional turmeric and ginger are modelled as fresh rhizomes at point of sale.

16.2 Environmental impacts of spices (EF3.1) – absolute values including Monte Carlo Simulation

Figure 14 shows the absolute values including the 95% confidence interval based on the Monte Carlo simulation. Negative values result from methodological inconsistencies in the implementation of material and emission flows in SimaPro and ecoinvent, which can lead to artificially negative outcomes.

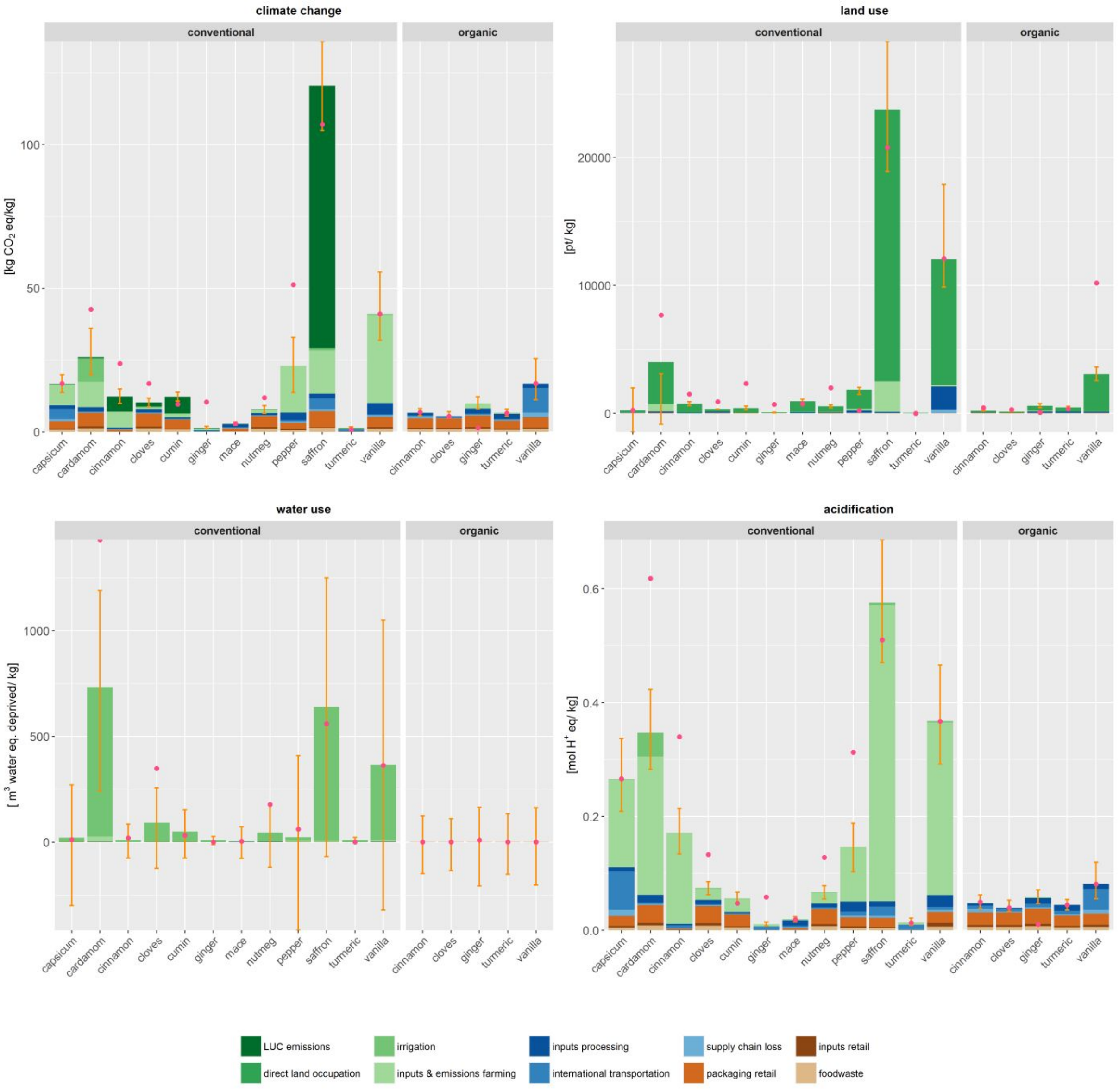

Figure 14: Absolute values of four midpoint impact categories of EF3.1 of 17 spices based on specific case studies (bars) and converted with global median yield (red dots. Error bars show the 95% confidence interval based on the Monte Carlo simulation. The process contributions are shown based on three stages: (1) cultivation: land use change emissions, occupation, irrigation, inputs & emissions farming (fertilizer, pesticides, seeds, cultivation methods); (2) processing: inputs processing (infrastructure, processes like drying), international transportation, supply chain loss; (3) retail: packaging retail, inputs retail (infrastructure, etc.), foodwaste. Conventional turmeric and ginger are modelled as fresh rhizomes at point of sale.

17.Results Biodiversity impact LUIF and LC-Impact & Monte Carlo Simulation

Figure 15 shows the absolute (left) and relative contribution (right) of LUIF and LC-Impact.

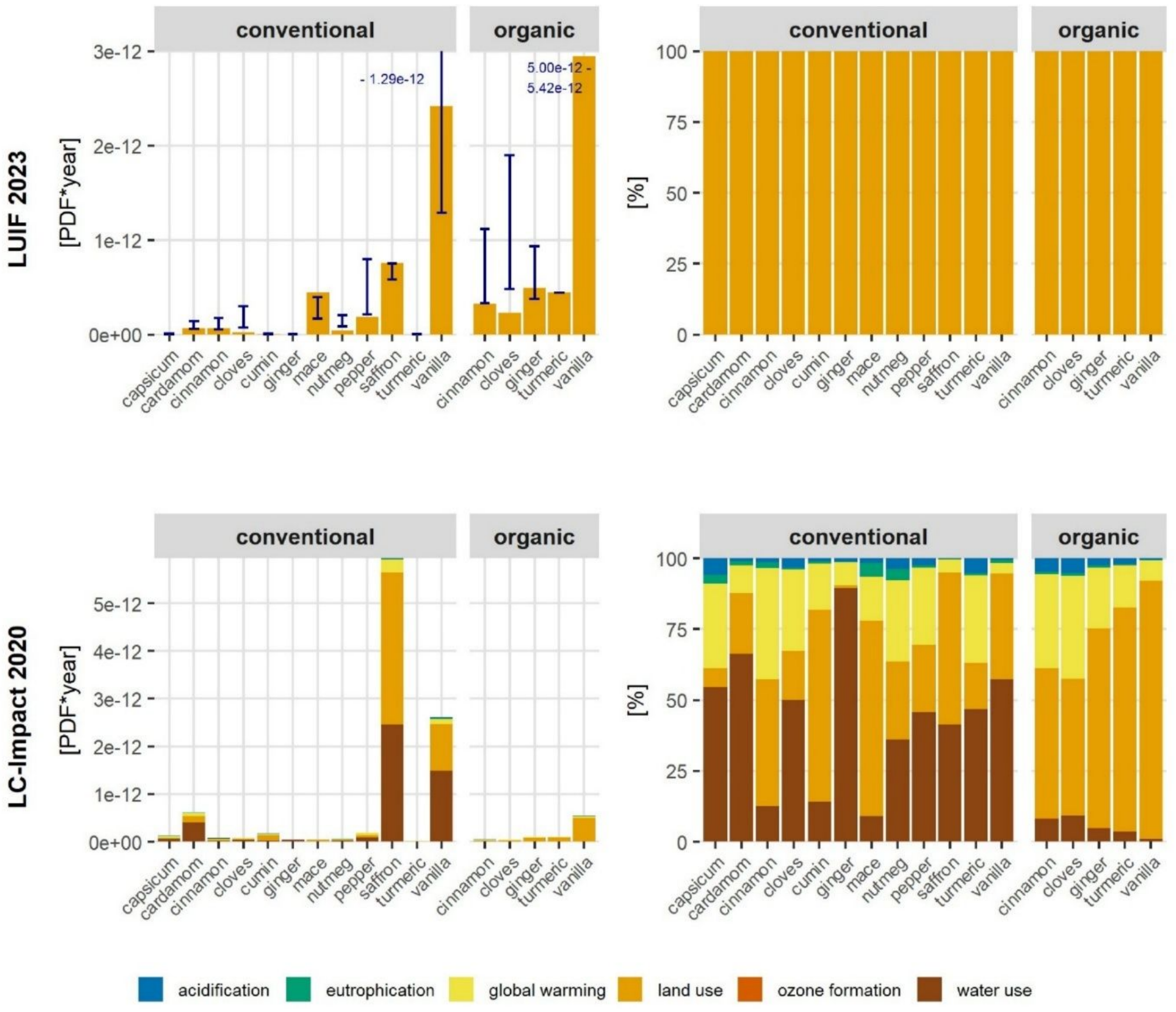

*Figure 15:Biodiversity impact in PDF*year per kg of spic in absolute values (left) and relative contributions of the pressures (right) calculated with LUIF and LC-Impact.*

Additionally, Figure 16 presents the absolute values of the LC-Impact assessment together with 95% confidence intervals derived from Monte Carlo simulations. Negative values result from methodological inconsistencies in the implementation of material and emission flows in SimaPro and ecoinvent, which can lead to artificially negative outcomes.

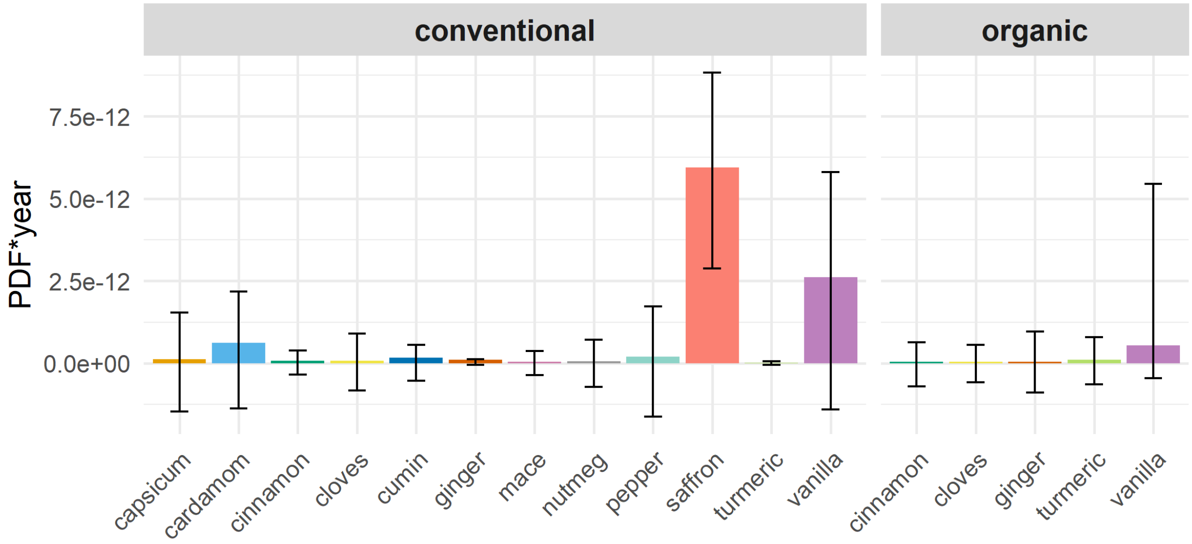

Figure 16: Absolute values of the potentially disappeared fraction of species (PDF·year) per kilogram of spice, calculated using LC-Impact. Error bars represent the 95% confidence interval based on Monte Carlo simulation.

18. Production and Economic Share

Twelve spices were analyzed (Figure 17): capsicum, cardamom, cinnamon, cloves, cumin, ginger, nutmeg, mace, pepper, saffron, turmeric, and vanilla. This selection is based on the share of the fresh production mass, the share of the economic value (FAOstat, 2022) and the classification by the HS Convention (World Customs Organisation, 2022).

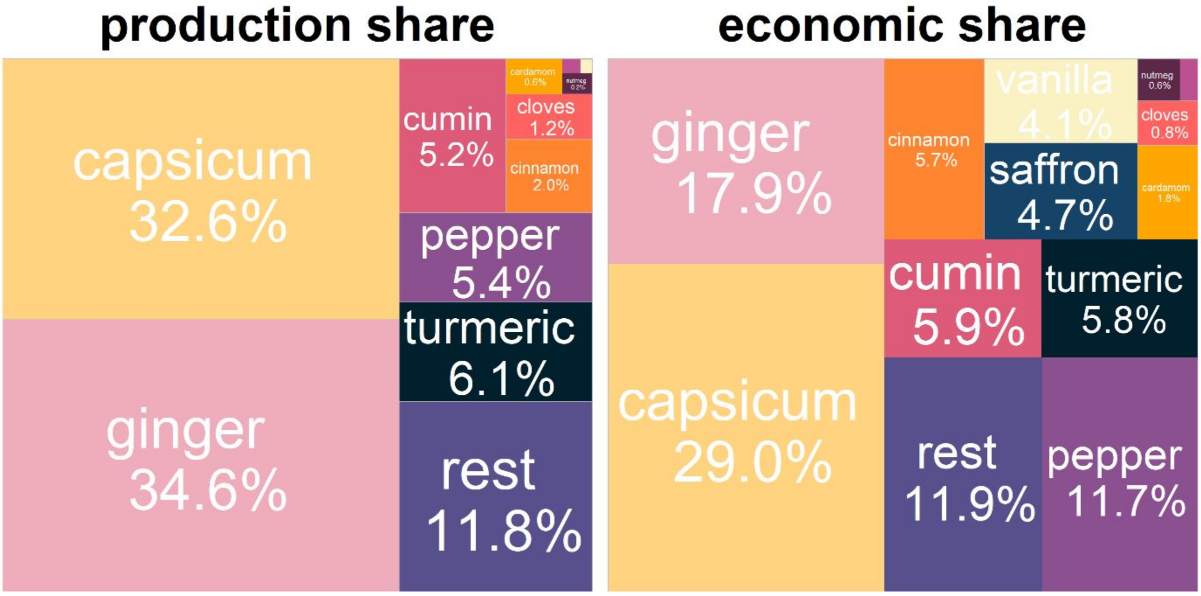

Figure 17: Production and economic share of the 12 most relevant spices (FAOstat, 2022; World Customs Organisation, 2022).

19. Sensitivity Analysis

LUC emissions

Since emissions associated with LUC are one of the main contributors to GHG emissions, a sensitivity analysis is included. LUC emissions can account for land use changes occurring over a 20-year or 50-year time horizon. UNEP-SETAC guidelines recommend using a time horizon of 20 years (Koellner et al., 2013). Longer time horizons (50 years) reduce GWP per year but may also encompass land conversion events that occurred within that period, which would not be considered in shorter time horizons (20 years). This would increase the total LUC emissions captured in the assessment. For comparison, the LUC emissions of a 50-year horizon are presented in Figure 18.

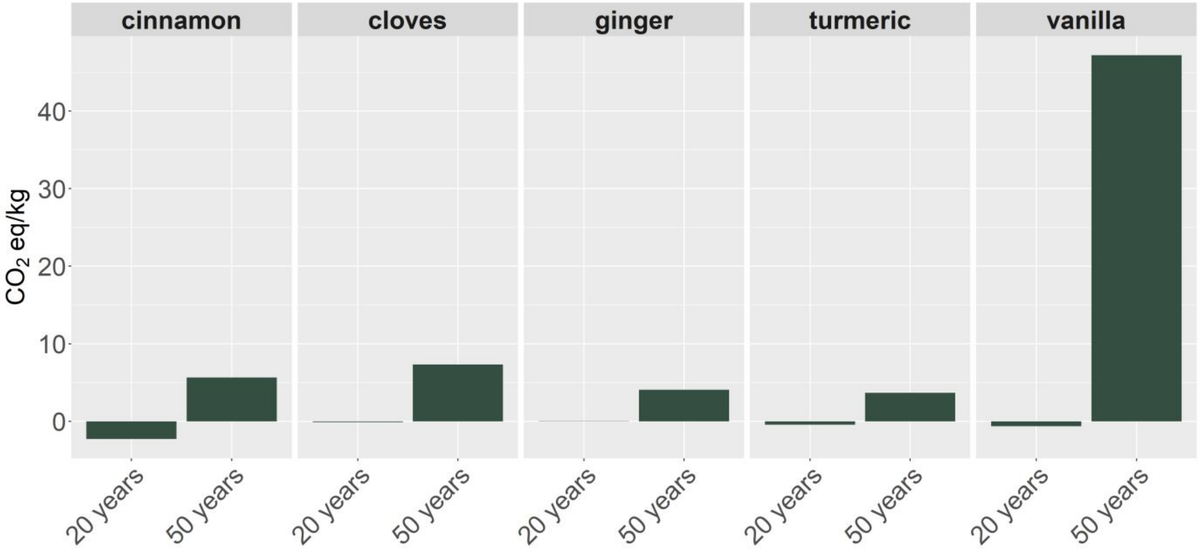

Figure 18:Sensitivity analysis of land use change emissions contributions of the five organic spices over two different time horizons: 20 and 50 years.

Over a 20-year horizon, the organic spice provider's organic spices show slight positive LUC effects, as cropland existed at these sites 20 years ago. For cinnamon, a perennial crop, former land use involved annual crops, leading to greater CO_2 storage today, reflected in positive CO_2-eq values. Table 29 shows the carbon storage for cinnamon, ginger, turmeric, vanilla, and clove, which has remained stable over the last 20 years. However, over a 50-year horizon, LUC emissions rise, particularly for vanilla and cloves, where cultivation replaced original rainforest areas.

*Table 29: Land use change emissions [t CO2-eq/ha*year] of the five organic spices over two time horizons: 20 and 50 years.*

spice	cultivation today	previous vegetation type (20 years ago)	previous vegetation type (50 years ago)	tCO2-eq/ha*y 20 y	tCO2-eq/ha*y 50 y
cinnamon	perennial cropland	annual cropland	tropical moist	-4.298	10.6
cloves	perennial cropland	perennial cropland, tropical moist	tropical rainforest	-0.190	14.1
ginger	annual cropland	annual cropland	tropical moist	-0.086	12.3
turmeric	annual cropland	annual cropland	tropical moist	-0.086	12.3
vanilla	perennial cropland	perennial cropland, tropical moist	tropical rainforest	-0.190	14.1

Packaging

Given the significant influence of retail glass packaging, a sensitivity analysis for whole nutmeg is conducted, comparing a plastic bag to a glass bottle. The plastic bag, weighing 3 g for 40 g of nutmeg, replaced a 100 g glass bottle for 38 g of nutmeg. Across all impact categories (Figure 19), the environmental impact decreased between 83.9% and 96.8%. For the single score of nutmeg, this means a decrease from 1.22 mPt (0.414 mPt from glass) to 0.792 mPt (0.067 mPt from plastic). Therefore, switching from glass packaging to plastic packaging is recommended.

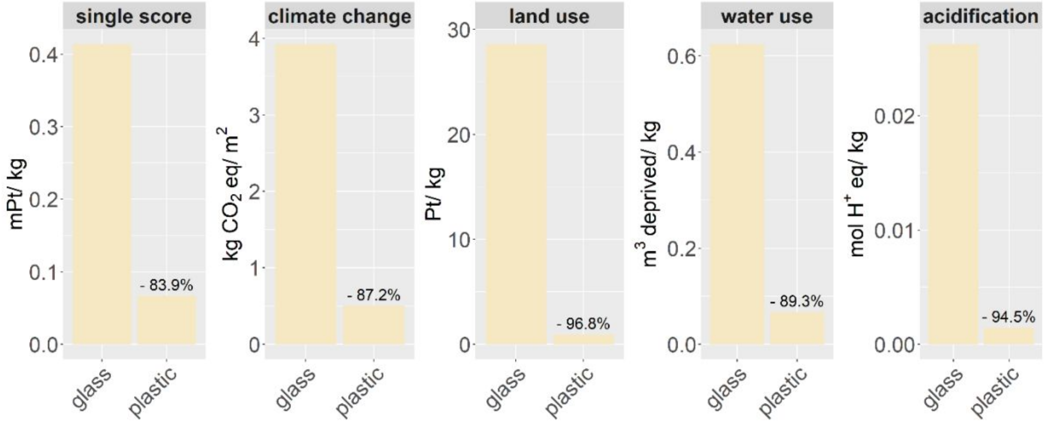

Figure 19: Sensitivity analysis of the retail packaging of the spice nutmeg with glass bottle packaging (left bar) and plastic bag packaging (right bar). The environmental impacts of four EF 3.1 midpoint impact categories and the single score EF 3.1 are shown.

Pillars

For pepper, the infrastructural part of pillars shows a significant influence on the result, which is why a sensitivity analysis is performed relative to two alternatives (Figure 20). The existing "concrete" system comprises two-thirds concrete pillars and one-third shade plants. The alternative "plants" system consists entirely of shade plants, while the "trellis" alternative is made up of two-thirds wood-based trellis and one-third plants. Both alternatives substantially reduce the impact of the farming stage. Within the climate change category, trellis and plant systems contribute just 0.42% and 0.56%, respectively, leading to a 59.8% reduction in climate change impact compared to concrete pillars. Concerning overall environmental impact (single score), concrete pillars account for 40.8%, while trellis and plant systems contribute 0.72% and 0.43%, respectively.

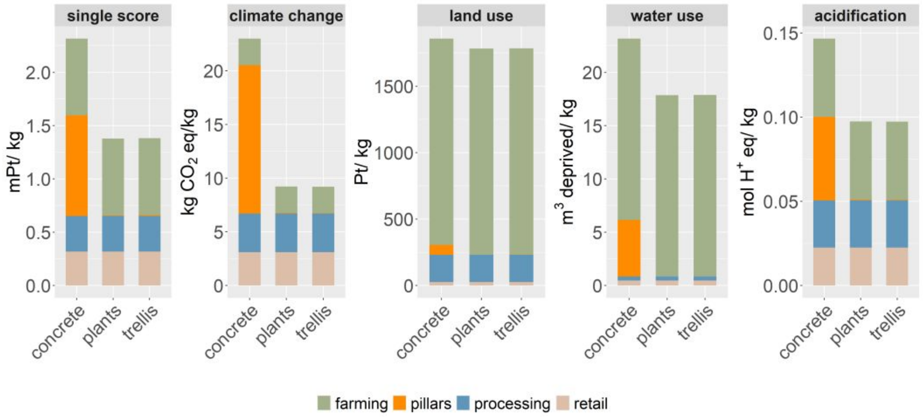

Figure 20: Sensitivity analysis of pillars in the pepper cultivation system. Scenario "concrete" refers to concrete pillars for 2/3 of the pepper plants and 1/3 plants. Scenario "plants" only uses plants, and scenario "trellis" uses 2/3 wooden trellis and 1/3 plants. Pillars' contribution is marked in orange but still belongs to the farming stage.

20. Yield of all Spices

Table 30 shows the fresh yield, the weight loss, and the fresh yield required for producing 1 kg dried spice.

Table 30: The spice's fresh yield, weight loss, required fresh yield for 1 kg spice at the point of sale and the dried yield.

spice	fresh yield [kg/ha]	weight loss [%]	fresh yield required [kg]	dry yield [kg/ha]
capsicum, conventional	50'706	75	4	12'676
cardamom, conventional	1'000	78	4.5	222
cinnamon organic	1'800	25	1.3	900
cinnamon, conventional	1'375	25	1.3	687.5
cloves, organic	2'560	25	1.3	1'920
cloves, conventional	3'546	25	1.3	2'659.5
cumin, conventional	649	5	1.05	617
ginger, organic	12'500	86	7	1'785.7
ginger, conventional	10'700			
mace, conventional	393.7	60	2.5	157.5
nutmeg, conventional	2'100	25	1.3	1'575
pepper, conventional	2'500	53	2.1	1'182.5
saffron, conventional	17.5	80	5	3.5
turmeric, organic	10'000	80	5	2'000
turmeric, conventional	17'700			
vanilla, conventional	350	83	5.9	59.5
vanilla, organic	1'200	75	4	300

21. Biodiversity method comparison

Using LC-Impact, direct impacts on biodiversity (land use) and indirect impacts are considered. The share of which is dominating is shown in Table 31 and shows a green background.

Table 31: Share of direct and indirect impact on species loss per spice in %. Green indicates whether direct or indirect impacts are dominating.

Spice	Direct impact, land use [%]	Indirect impact [%]
Capsicum	0.2	99.8
Cardamom	11.9	88.1
Cinnamon conventional	63.1	36.9
Cinnamon organic	93.7	6.26
Cloves conventional	28.0	72.0
Cloves organic	92.4	7.63
Cumin	7.15	92.9
Ginger conventional	2.8	97.2
Ginger organic	94.0	6.0
Mace	97.1	2.9
Nutmeg	49	51.0
Pepper	56.0	44.0
Saffron	21.6	78.4
Turmeric conventional	7.2	92.8
Turmeric organic	95.4	4.6
Vanilla conventional	59.8	40.2
Vanilla organic	98.4	1.6

Literature

Agri Farming. (2015, March 24). *Clove Cultivation, Planting, Care, Harvesting Guide | Agri Farming*. https://www.agrifarming.in/clove-cultivation-information

Aluthgamage, H., Fonseka, D. L. C., & Nakandalage, N. (2023). Enhancement of high-quality cinnamon quill production through agronomic approaches: A review. *Academia Biology*. https://doi.org/10.20935/AcadBiol6025

Aluthgamage, H. N., Fonseka, D. L. C. K., & Benaragama, C. K. (2021). Study the cinnamon (Cinnamomum verum J. Presl) yield indices under modified planting systems. *Tropical Agricultural Research and Extension*, *24*(2), 116. https://doi.org/10.4038/tare.v24i2.5503

Andabjadid, S. S., Eslam, B. P., Sadeghi Bakhtavari, A. R., & Mohammadi, H. (2015). Effects of corm size and plant density on Saffron (Crocus sativus L.) yield and its components. *6*, *3*, 20–26.

Ankegowda, S., Biju, C., Jayashree, E., Prasath, D., Praveena, R., & Senthil Kumar, C. (2015). *cardamom. ICAR - Indian Institute of Spices Research.* https://krishi.icar.gov.in/jspui/bitstream/123456789/26076/1/cardamom.pdf

Apnikheti. (2024). *Capsicum Farming in Punjab | Farming Guide | Apni Kheti*. https://www.apnikheti.com/en/pn/agriculture/horticulture/vegetable-crops/capsicum

Aravind, S., Biju, C. N., Krishnamurthy, K. S., Radha, E., Nissar V.A., M., Aarthi, S., & Eapen, S. J. (2020). *ICAR - AICRPS Technologies. ICAR-ALL India coordinated research project on spices.* http://spices.res.in/sites/default/files/TECHNOLOGY%202021.pdf

Arenas, M. A. S., & Dressler, R. L. (2009). A revision of the Mexican and Central American species of *Vanilla* plumier ex miller with a characterization of their its region of the nuclear ribosomal DNA. *Lankesteriana: International Journal on Orchidology*. https://doi.org/10.15517/lank.v0i0.12065

Asadi, G. A., Khorramdel, S., & Hatefi Farajian, M. H. (2016). The Effects of Row Intercropping Ratios of Chickpea and Saffron on Their Quantitative Characteristics and Yield. *Saffron Agronomy and Technology*, *4*(2), 93–103. https://doi.org/10.22048/jsat.2016.17360

Azam-Ali, S. (2007). *Production and processing of nutmeg and mace. Practical Act. Technology challenging poverty.*

https://ftpmirror.your.org/pub/misc/cd3wd/1002/_ag_proc_fp_KnO_100266_nutmeg_pa_en_115640_.pdf

Borken-Kleefeld, J. (2012). *Default transport data per commodity group for the US and EU27 – Methodology and notes on data for ecoinvent, ecoinvent Centre*.

Britannica. (2024, February 27). *Cinnamon | Plant, Spice, History, & Uses | Britannica*. https://www.britannica.com/plant/cinnamon

Business Insider (Director). (2021, March 13). *Why Ceylon Cinnamon Is So Expensive | So Expensive* [Video recording]. https://www.youtube.com/watch?v=mn6650M3M-U

Cagliani, L. R., Culeddu, N., Chessa, M., & Consonni, R. (2015). NMR investigations for a quality assessment of Italian PDO saffron (Crocus sativus L.). *Food Control*, *50*, 342–348. https://doi.org/10.1016/j.foodcont.2014.09.017

Cardone, L., Castronuovo, D., Perniola, M., Cicco, N., & Candido, V. (2020). Saffron (Crocus sativus L.), the king of spices: An overview. *Scientia Horticulturae*, *272*, 109560. https://doi.org/10.1016/j.scienta.2020.109560

Carvalho, D. F. de, Teles, G. C., Cruz, E. S. da, Valença, D. da C., & Medici, L. O. (2023). Yield response factor (Ky) and initial growth in black pepper in a tropical environment. *Scientia Agricola*, *80*, e20220171. https://doi.org/10.1590/1678-992X-2022-0171

Chambers, A. H., Moon, P., Edmond, V., & Bassil, E. (2019). Vanilla Cultivation in Southern Florida: HS1348, 11/2019. *EDIS*, *2019*(6), Article 6. https://doi.org/10.32473/edis-hs1348-2019

Chaudhary, A., Verones, F., de Baan, L., & Hellweg, S. (2015). Quantifying Land Use Impacts on Biodiversity: Combining Species–Area Models and Vulnerability Indicators. *Environmental Science & Technology*, *49*(16), 9987–9995. https://doi.org/10.1021/acs.est.5b02507

Chen, D., Xing, B., Yi, H., Li, Y., Zheng, B., Wang, Y., & Shao, Q. (2020). Effects of different drying methods on appearance, microstructure, bioactive compounds and aroma compounds of saffron (*Crocus sativus* L.). *LWT*, *120*, 108913. https://doi.org/10.1016/j.lwt.2019.108913

Costello, M. J., Bouchet, P., Boxshall, G., Fauchald, K., Gordon, D., Hoeksema, B. W., Poore, G. C. B., Soest, R. W. M. van, Stöhr, S., Walter, T. C., Vanhoorne, B., Decock, W., & Appeltans, W. (2013). Global Coordination and Standardisation in Marine Biodiversity through the World

Register of Marine Species (WoRMS) and Related Databases. *PLOS ONE*, *8*(1), e51629. https://doi.org/10.1371/journal.pone.0051629

Dar, M., Groach, R., Razvi, S. M., & Singh, N. (2017). *Saffron Crop (Golden Crop) in Modern Sustainable Agricultural Systems*. *5*(6). https://www.researchgate.net/publication/322488066_Saffron_Crop_Golden_Crop_in_Modern_Sustainable_Agricultural_Systems

Darekar, N., Paslawar, A., & Watane, A. (2021). *Agrotechniques for organic turmeric production and processing*. *10*(12), 256–266.

De Silva, A., & Esham, M. (2020). *Ceylon Cinnamon Production and Markets* (pp. 63–84). https://doi.org/10.1007/978-3-030-54426-3_3

Devasahayam, S., John, Z. T., Jayashree, E., Kandiannan, K., Prasath, D., Santhosh, J. E., Srinivasan, V., & Suseela, B. R. (2015). *black pepper. ICAR - Indian Institute of Spices Research.*

Díaz, S., Settele, J., Brondízio, E. S., Ngo, H. T., Guèze, M., Agard, J., Arneth, P., Balvanera, P., Brauman, K. A., Butchart, S. H. M., Chan, K. M. A., Garibaldi, L. A., Ichii, K., Liu, J., Subramanian, S. M., Midgley, G. F., Miloslavich, P., Molnár, Z., Obura, D., … Zayas, C. N. (2019). *Summary for Policymakers of the global assessment report on biodiversity and ecosystem services of the Intergovernmental Science-Policy Platform on Biodiversity and Ecosystem Services (IPBES).* (SUMMARY FOR POLICYMAKERS). https://files.ipbes.net/ipbes-web-prod-public-files/inline/files/ipbes_global_assessment_report_summary_for_policymakers.pdf

Duwe, K. (2022). *Pfeffer (Piper nigrum) | Pflanzen-lexikon.com*. https://www.pflanzen-lexikon.com/Box/Piper_nigrum.html

ecoinvent v3.10. (2024). *Ecoinvent v3.10*. https://ecoinvent.org/the-ecoinvent-database/data-releases/ecoinvent-v3-10/

EDB, E. D. B. S. L. (2021). *The Difference between Cassia and True Ceylon Cinnamon*. https://www.srilankabusiness.com/blog/difference-between-cassia-and-ceylon-cinnamon.html

FAO. (2022). *Agroecology Knowledge Hub—Food and Agriculture Organization of the United Nations*. http://www.fao.org/agroecology/home/en/

FAO, F. and A. O. of the U. N. (2018). *Detailed Information | GIAHS - 全球重要农业文化遗产 | 联合国粮食及 农业组织 | GIAHS | Food and Agriculture Organization of the United Nations*. https://www.fao.org/giahs/giahsaroundtheworld/designated-sites/asia-and-the-pacific/qanat-based-saffron-farming-system-in-gonabad/detailed-information/zh/

FAO, F. and A. O. of the U. N. (2021). *JOINT FAO/WHO FOOD STANDARDS PROGRAMME - CODEX COMMITTEE ON SPICES AND CULINARY HERBS. PROPOSAL FOR NEW WORK ON A CODEX STANDARD FOR TURMERIC.*

FAO, F. and A. O. of the U. N. (2024). *Pepper—Crop Description and Climate.* https://www.fao.org/land-water/databases-and-software/crop-information/pepper/fr/

FAOstat. (2022). *Food and Agriculture Organization of theUnited Nations—Data*. https://www.fao.org/faostat/en/#data/QCL

Fluent Cargo. (2024). *Routing—Fluent Cargo*. https://www.fluentcargo.com/search?origin=indonesia&destination=india

Ghorbani, R., & Koocheki, A. (2017). *Sustainable Cultivation of Saffron in Iran* (pp. 169–203). https://doi.org/10.1007/978-3-319-58679-3_6

GoComet. (2024, February 22). *Top 12 Major Ports In North America 2024: Leading The Maritime Economy*. https://www.gocomet.com/blog/top-ports-in-north-america/

Hajyzadeh, M., Asil, H., Yildirim, M. U., Sarihan, E. O., Ayanoglu, F., & Khawar, K. M. (2017). Evaluating effects of corm circumference and storage temperatures on yield and yield components of saffron at different elevations. *Acta Horticulturae*, *1184*, 39–46. https://doi.org/10.17660/ActaHortic.2017.1184.6

Hernández, J. H. (2019). Mexican Vanilla Production. In *Handbook of Vanilla Science and Technology* (2nd ed.). West Sussex: Blackwell Publishing Ltd. https://doi.org/10.1002/9781444329353

HSBC. (2024). *Growing Chinese Businesses in Singapore*. https://www.business.hsbc.com.sg/en-sg/campaigns/china-sg-corridor

Husaini, A., bullet, Hasan, B., Muzaffar, bullet, Ghani, Y., Silva, J., Nayar, bullet, & Kirmani, N. (2010). *Saffron (Crocus sativus Kashmirianus) Cultivation in Kashmir: Practices and*

Problems. https://www.researchgate.net/publication/232607443_Saffron_Crocus_sativus_Kashmirianus_Cultivation_in_Kashmir_Practices_and_Problems

IFEAT. (2022). *Pepper—The King of Spices. IFEAT Socio-economic report.* https://ifeat.org/wp-content/uploads/2022/05/Pepper-by-Geemon-Korah.pdf

India Agro. (2024). *Clove Crop Cultivation Guide | Clove Cultivation | Clove Farming | IndiaAgroNet*. https://indiaagronet.com/Horticulture/CONTENTS/clove.htm

ITC. (2022). *Trade Map—List of products at 6 digits level exported in 2022*. World Trade Centre. https://www.trademap.org/Product_SelProductCountry.aspx?nvpm=1%7c%7c%7c%7c%7c0908%7c%7c%7c6%7c1%7c1%7c2%7c1%7c1%7c1%7c1%7c1%7c1

K Agriculture. (2022a, January 5). *Cinnamon Production And Things You Should Know | K-Agriculture*. https://k-agriculture.com/cinnamon-production/

K Agriculture. (2022b, September 15). *1 new message*. https://k-agriculture.com/secrets-that-make-vietnam-pepper-cultivation-special-you-have-to-know/

Kafi, M., Kamili, A., Husaini, A., Ozturk, M., & Altay, V. (2018). An Expensive Spice Saffron (Crocus sativus L.): A Case Study from Kashmir, Iran, and Turkey. In *Global Perspectives on Underutilized Crops* (pp. 109–149). https://doi.org/10.1007/978-3-319-77776-4_4

Kaufman. (2016). *Zingiber officinale (ginger) | CABI Compendium. CABI Compendium*. https://doi.org/10.1079/cabicompendium.5753

Kembauw, E., Mahulette, A. S., Senewe, R. E., Wattimena, A. Y., Kastanya, A., Lailossa, A. A., Wokanubun, A. I., & Samal, R. (2023). Cultivation System and Marketing Chain of Nutmeg in East Seram District, Maluku Province, Indonesia. *International Journal of Multidisciplinary Sciences and Arts*, *2*(1), Article 1. https://doi.org/10.47709/ijmdsa.v1i2.2015

Koellner, T., de Baan, L., Beck, T., Brandão, M., Civit, B., Margni, M., i Canals, L. M., Saad, R., de Souza, D. M., & Müller-Wenk, R. (2013). UNEP-SETAC guideline on global land use impact assessment on biodiversity and ecosystem services in LCA. *The International Journal of Life Cycle Assessment*, *18*(6), 1188–1202. https://doi.org/10.1007/s11367-013-0579-z

Koocheki, A., Rezvani Moghaddam, P., & Seyyedi, S. M. (2019). Depending on mother corm size, the removal of extra lateral buds regulates sprouting mechanism and improves phosphorus acquisition efficiency in saffron (*Crocus sativus* L.). *Industrial Crops and Products*, *141*, 111779. https://doi.org/10.1016/j.indcrop.2019.111779

Kothari, D., Thakur, R., & Kumar, R. (2021). Saffron (Crocus sativus L.): Gold of the spices—a comprehensive review. *Horticulture, Environment, and Biotechnology*, *62*. https://doi.org/10.1007/s13580-021-00349-8

Kumar, R., Singh, V., Devi, K., Sharma, M., Singh, M. K., & Ahuja, P. S. (2008). State of Art of Saffron (Crocus sativus L.) Agronomy: A Comprehensive Review. *Food Reviews International*, *25*(1), 44–85. https://doi.org/10.1080/87559120802458503

Leone, S., Recinella, L., Chiavaroli, A., Orlando, G., Ferrante, C., Leporini, L., Brunetti, L., & Menghini, L. (2018). Phytotherapic use of the Crocus sativus L. (Saffron) and its potential applications: A brief overview. *Phytotherapy Research*, *32*(12), 2364–2375. https://doi.org/10.1002/ptr.6181

Locey, K. J., & Lennon, J. T. (2016). *Scaling laws predict global microbial diversity—PubMed*. *21*(113), 5970–5975. https://doi.org/doi: 10.1073/pnas.1521291113.

Lubinsky, P., Van Dam, M., & Van Dam, A. (2006). Pollination of Vanilla and evolution in Orchidaceae. *Lindleyana*, *75*, 926–929.

Lupi, M. (2023). *Sozio-ökonomische Situation der Vanillebauern in Mexiko. Fallstudie in Veracruz und Puebla. Masterarbeit in der Forschungsgruppe Lebensmittel-Chemie der ZHAW.*

Mariyam, A. (2023, December 29). *Vietnam Model Black Pepper Cultivation Practice | PepperHub*. https://www.pepperhub.in/vietnam-model-black-pepper-cultivation/

Meena, R. S., Anwer, M. M., Lal, C., Kant, K., & Mehta, R. S. (2021). *Advance Production Technology of Cumin. ICAR - National Research Centre on Seed Spices, Tabiji, Ajmer-305206 (Rajasthan)*. https://nrcss.icar.gov.in/Upload/637876998055478246.pdf

Menia, M., Iqbal, S., R, Z., S, T., Rh, K., Aa, S., & Hussian, A. (2018). Production technology of saffron for enhancing productivity. *Journal of Pharmacognosy and Phytochemistry*, *7*(1), 1033–1039.

Mohammad. (2012). Respond of saffron (Crocus sativus L.) to animal manure application. *Journal of Medicinal Plants Research*, *6*(7). https://doi.org/10.5897/JMPR11.1442

Mollafilabi, A., Davari, K., & Dehaghi, M. A. (2021). Saffron yield and quality as influenced by different irrigation methods. *Scientia Agricola*, *78*(1), e20190084. https://doi.org/10.1590/1678-992x-2019-0084

Mora, C., Tittensor, D. P., Adl, S., Simpson, A. G. B., & Worm, B. (2011). How Many Species Are There on Earth and in the Ocean? *PLOS Biology*, *9*(8), e1001127. https://doi.org/10.1371/journal.pbio.1001127

Moratalla-López, N., Parizad, S., Habibi, M. K., Winter, S., Kalantari, S., Bera, S., Lorenzo, C., García-Rodríguez, M. V., Dizadji, A., & Alonso, G. L. (2021). Impact of two different dehydration methods on saffron quality, concerning the prevalence of S*affron latent virus* (SaLV) in Iran. *Food Chemistry*, *337*, 127786. https://doi.org/10.1016/j.foodchem.2020.127786

Murugan, M., Ashokkumar, K., Alagupalamuthirsolai, M., Anandhi, A., Ravi, R., Dhanya, M. K., & Sathyan, T. (2022). Understanding the Effects of Cardamom Cultivation on Its Local Environment Using Novel Systems Thinking Approach-the Case of Indian Cardamom Hills. *Frontiers in Sustainable Food Systems*, *6*. https://www.frontiersin.org/articles/10.3389/fsufs.2022.728651

Nehvi, F. A., Dhar, J. K., & Wani, S. A. (2014). *National Agricultural Innovation Project (Indian Council of Agricultural Research)—A VALUE CHAIN ON KASHMIR SAFFRON.*

Nemecek, T., Bengoa, X., Lansche, J., Roesch, A., Faist-Emmenegger, M., Rossi, V., Humbert, S., Brunner, Guignard, C., & Burg, A. (2023). *Methodological Guidelines for the Life Cycle Inventory of Agricultural Products. World Food LCA Database (WFLDB). Version 3.9. Quantis and Agroscope.*

Noyce, P. A., & Crevello, G. L. (2016). *STRUCTURE magazine | Durability of Reinforced Concrete*. https://www.structuremag.org/?p=9459

Oliveira, W., Beneduzzi, H., Alves, F., Coelho, S., Mendonça, A., Silva, R., & Da. (2021). Production Chain of Black Pepper in Brazil. *Nucleus*, *18*, 101–116. https://doi.org/10.3738/1982.2278.3826

Pack, S. (2020, August 25). Steps of sending and transporting export-grade (export-quality) saffron. *SadafPack*. https://sadafpack.com/blog/steps-of-sending-and-transporting-export-grade-export-quality-saffron/

Persistance Market Research. (2018). *Global Market Study on Cinnamon: Rising Demand for Healthy and Flavorful Spices Augmenting Market Growth*. Persistence Market Research. https://www.persistencemarketresearch.com/market-research/cinnamon-market.asp

Pfister, S., Bayer, P., Koehler, A., & Hellweg, S. (2011). Environmental Impacts of Water Use in Global Crop Production: Hotspots and Trade-Offs with Land Use. *Environmental Science & Technology*, *45*(13), 5761–5768. https://doi.org/10.1021/es1041755

Piyasiri, K. H. K. L., & Wijkeratne, M. (2016). *Comparison of the Cultivated Area and the Production Trends of Ceylon Cinnamon with the Main Competitors in the Worlds' Total Cinnamon Market*. *6*(1).

Ramos-Castellá, A., Iglesias Andreu, L., Bello-Bello, J., & Lee, H. (2014). Improved propagation of vanilla (Vanilla planifolia Jacks. Ex Andrews) using a temporary immersion system. *In Vitro Cellular & Developmental Biology - Plant*, *50*. https://doi.org/10.1007/s11627-014-9602-8

Rawat, I., Verma, N., & Joshi, K. (2020). Cinnamon (Cinnamomum zeylanicum). In *Medicinal Plants in India: Importance and Cultivation* (pp. 164–177). Jaya Publishing House.

Rema Shree, A., Dhanapal, K., Pradip Kumar, K., Ansar Ali, A., & John Jo, V. (2021). *Cultivation practices for small Cardamom. Indian Cardamom Research Institute. Spices Board India, Kochi.* https://www.indianspices.com/sites/default/files/plant_protection_small.pdf

Robertson, S. M., Schmid, R. B., & Lundgren, J. G. (2023). Estimating plant biomass in agroecosystems using a drop-plate meter. *PeerJ*, *11*, e15740. https://doi.org/10.7717/peerj.15740

Roslan, F., & M. Yudin, A. (2020). Drying process of black pepper in a swirling fluidized bed dryer using experimental method. *IOP Conference Series: Materials Science and Engineering*, *863*, 012047. https://doi.org/10.1088/1757-899X/863/1/012047

Rymbai, H., Das, A., Mohapatra, K., Talang, H., Nongbri, B., & Law, I. (2021). Ginger (Zingiber officinale) based intercropping systems for enhancing productivity and income -a farmers'

participatory approach. *Indian Journal of Agricultural Sciences*, *91*, 956–960. https://doi.org/10.56093/ijas.v91i7.115024

Salwee, Y., & Nehvi, F. A. (2013). *Saffron as a valuable spice: A comprehensive review*. *8*(3), 234–242. https://doi.org/10.5897/AJAR12.1955

Saravanakumar, D. (2021). *A guide to good agricultural practices for commercial production of ginger under field conditions in Jamaica. FAO.* https://doi.org/10.4060/cb3365en

SeaRates. (2024). *Cargo Calculator | Sea Distance Calculator for Shipping*. SeaRates. https://www.searates.com/services/distances-time

Sepaskhah, A. R., & Kamgar, H. (2009). *Saffron Irrigation Regime*. 1–16.

Shamina, M. (2022). Improved Farming Practices for the Cultivation of Spice, Clove (Syzygium aromaticum) in India. *Bhartiya Krishi Anusandhan Patrika*, *37*(1). https://arccjournals.com/journal/bhartiya-krishi-anusandhan-patrika/BKAP387

Sharma, R., Joshi, V., & Kaushal, M. (2015). Effect of pre-treatments and drying methods on quality attributes of sweet bell-pepper (Capsicum annum) powder. *Journal of Food Science and Technology*, *52*(6), 3433–3439. https://doi.org/10.1007/s13197-014-1374-y

Spence, C. (2024). Nutmeg and mace: The sweet and savoury spices. *International Journal of Gastronomy and Food Science*, *36*, 100936. https://doi.org/10.1016/j.ijgfs.2024.100936

Stafford, P. (2021, February 10). Amsterdam ousts London as Europe's top share trading hub. *Financial Times*. https://www.ft.com/content/3dad4ef3-59e8-437e-8f63-f629a5b7d0aa

Stamer, S. (2022, November 29). *Gesrpäch zum Anbau von Schwarzem Pfeffer. Durchgeführt von Roger Vogel.* [Personal communication].

Statistia. (2019). *Saffron: Leading producers worldwide 2019.* Statista. https://www.statista.com/statistics/1135621/leading-saffron-producers-worldwide/

Sumbula, V., & Mathew, S. K. (2015). *Management of Phytophthora leaf fall disease of nutmeg (Myristica fragrans houtt.)*. *53*, 180–186.

Suriyagoda, L., Mohotti, A. J., Vidanarachchi, J. K., Kodithuwakku, S. P., Chathurika, M., Bandaranayake, P. C. G., Hetherington, A. M., & Beneragama, C. K. (2021). "Ceylon

cinnamon”: Much more than just a spice. *PLANTS, PEOPLE, PLANET*, *3*(4), 319–336. https://doi.org/10.1002/ppp3.10192

Syngenta. (2018, June 25). *Gesaprim Granules Herbicide | Grass & Broadleaf Weed Control*. Syngenta Australia. https://www.syngenta.com.au/product/crop-protection/gesaprim-granules

Talattad, M., Keller, R., & Trachsel, S. (2021). *Environmental and social hotspots of vanilla production in São Tomé and Principe.* Institute of Natural Resource Sciences, Zurich University of Applied Sciences.

TIS, ransport I. S. (2024). *Saffron – Transport Informations Service*. https://www.tis-gdv.de/tis_e/ware/gewuerze/safran/safran-htm/

TNAU. (2022). *Horticulture: Cardamom*. https://agritech.tnau.ac.in/horticulture/horti_spice%20crops_cardamom.html

TNAU Agritech. (2013). *Horticulture. Spice Crops: Nutmeg*. https://agritech.tnau.ac.in/horticulture/horti_spice%20crops_nutmeg.html

TNAU Agritech. (2022). *Horticulture—Spice Crops—Pepper*. https://agritech.tnau.ac.in/horticulture/horti_spice%20crops_pepper.html

Tripodi, P., & Kumar, S. (2019). *The Capsicum Crop: An Introduction* (pp. 1–8). https://doi.org/10.1007/978-3-319-97217-6_1

UGOOD. (n.d.). *Cumin Pepper Fennel Spices Threshing Cleaning Machine*. Retrieved 13 August 2024, from https://www.corn-thresher.com/products/Crops-Threshing-Machine/Cumin-Pepper-Threshing-Machine.html

Vogel, R. (2023). *Auswirkungen des Klimawandels auf den Anbau von Pfeffer. Projektarbeit in der Forschungsgruppe Geography of Food an der ZHAW.* ZHAW.

World Bank. (2018). *World Bank Country and Lending Groups – World Bank Data Help Desk*. https://datahelpdesk.worldbank.org/knowledgebase/articles/906519-world-bank-country-and-lending-groups
